# Antiplatelet drugs for secondary prevention in patients with ischemic stroke or transient ischemic attack: a systematic review and network meta-analysis

**DOI:** 10.1186/s12883-021-02341-2

**Published:** 2021-08-16

**Authors:** Cinzia Del Giovane, Giorgio B. Boncoraglio, Lorenza Bertù, Rita Banzi, Irene Tramacere

**Affiliations:** 1 Institute of Primary Health Care (BIHAM), University of Bern, Bern, Switzerland; 2 Population Health Laboratory, University of Fribourg, Fribourg, Switzerland; 3 Department of Cerebrovascular Disease, Fondazione IRCCS Istituto Neurologico Carlo Besta, Milan, Italy; 4 Department of Research and Clinical Development, Fondazione IRCCS Istituto Neurologico Carlo Besta, Milan, Italy; 5 Center for Health Regulatory Policies, Istituto di Ricerche Farmacologiche Mario Negri IRCCS, Milan, Italy

**Keywords:** Systematic review, Network meta-analysis, RCT, Stroke, Secondary prevention, Antiplatelet drugs

## Abstract

**Background:**

Antiplatelet drugs may prevent recurrent ischemic events after ischemic stroke but their relative effectiveness and harms still need to be clarified. Within this network meta-analysis we aimed to summarize the current evidence for using antiplatelet drugs for secondary stroke prevention.

**Methods:**

We searched MEDLINE, EMBASE and CENTRAL up to September 2020. Randomized controlled trials (RCTs) assessing antiplatelet drugs for secondary stroke prevention were included. We did pairwise meta-analyses and network meta-analyses using random-effects models. Primary outcomes were all strokes (ischemic or hemorrhagic) and all-cause mortality.

**Results:**

The review included 57 RCTs, 50 (*n* = 165,533 participants) provided data for the meta-analyses. Compared to placebo/no treatment, moderate to high-confidence evidence indicated that cilostazol, clopidogrel, dipyridamole + aspirin, ticagrelor, ticlopidine, and aspirin ≤ 150 mg/day significantly reduced the risk of all strokes (odds ratios, ORs and absolute risk difference, ARD): cilostazol 0.51 (95 % confidence interval, CI, 0.37 to 0.71; 3.6 % fewer), clopidogrel 0.63 (95 % CI, 0.49 to 0.79; 2.7 % fewer), dipyridamole + aspirin 0.65 (95 % CI, 0.55 to 0.78; 2.5 % fewer), ticagrelor 0.68 (95 % CI, 0.50 to 0.93; 2.3 % fewer), ticlopidine 0.74 (95﻿ % CI 0.59 to 0.93; 1.9 % fewer), aspirin ≤ 150 mg/day 0.79 (95 % CI, 0.66 to 0.95; 1.5 % fewer). Aspirin > 150 mg/day and the combinations clopidogrel/aspirin, ticagrelor/aspirin, also decrease all strokes but increase the risk of hemorrhagic events. Only aspirin > 150 mg/day significantly reduced all-cause mortality (OR 0.86, 95 % CI 0.76 to 0.97; ARD 0.9 %, 95 %CI 1.5–0.2 % fewer, moderate confidence). Compared to aspirin ≤ 150 mg/day, clopidogrel significantly reduced the risk of all strokes, cardiovascular events, and intracranial hemorrhage outcomes. Cilostazol also appeared to provide advantages but data are limited to the Asian population.

**Conclusions:**

Considering the benefits and harms ratio, cilostazol, clopidogrel, dipyridamole + aspirin, ticagrelor, ticlopidine, and aspirin ≤ 150 mg/day appear to be the best choices as antiplatelet drugs for secondary prevention of patients with ischemic stroke or TIA.

**Systematic review registration:**

PROSPERO CRD42020159896.

**Supplementary Information:**

The online version contains supplementary material available at 10.1186/s12883-021-02341-2.

## Background

Despite a pronounced decrease in age-standardized mortality rates, stroke burden remains high. People living with stroke in the European Union is estimated to increase by one third between 2017 and 2047. This shift in stroke burden from mortality to morbidity depends on demographics change over time, implementation of primary prevention strategies, and availability of better care and treatment both in the acute and long-term stages after stroke [1].

The annual risk for future ischemic stroke after a stroke or transient ischemic attack (TIA) is 3–5 %, accounting for 25–30 % of all strokes. These patients are also at higher risk for subsequent myocardial infarction and death from vascular causes [2–5].

The risk of stroke is highest in the early period after the acute event. Prompt initiation of tailored prevention strategies, e.g., referral to acute stroke units, immediate antithrombotic drugs, early carotid revascularisation, is essential. In the longer term, optimal medical therapy, including antiplatelet and statin therapy, and risk factor modification, are recommended [4, 6]. The initiation or resumption of antihypertensive therapy, accompanied by lifestyle modifications, may be beneficial for patients with known hypertension [6].

Appropriate antithrombotic medication should be carefully chosen considering the ischemic injury physio-pathological mechanism: antiplatelet drugs are preferred for lesions characterized by atherosclerosis and vascular injury, whereas anticoagulant medications are indicated for cardioembolisms and thrombophilic conditions [7].

Antiplatelet drugs are widely recommended for non-cardioembolic stroke. Different drugs are currently used as monotherapies or combination therapies for prevention of vascular events among patients with stroke or TIA, given their effect in reducing the risk of stroke, myocardial infarction and death [6, 8]. Historically, the role of aspirin in preventing any type of stroke among patients with a recent stroke or TIA has been demonstrated, with a similar magnitude of benefit for doses ranging from 75 to 1500 mg but a marked difference in toxicity. Platelet Adenine di-Phosphate receptor antagonist, as ticlopidine and clopidogrel, and dual-therapies, as the combination of aspirin and dipyridamole were shown to be effective for secondary stroke prevention [4]. Newer agents, such as ticagrelor, may offer pharmacokinetics advantages over similar drugs. However, the relative effectiveness of these approaches is unclear, and the different safety profile, costs, patient characteristics, and preferences may affect the selection among agents for long-term secondary prevention.

Network meta-analysis (NMA) includes multiple interventions within a single analysis and allows researchers to estimate the relative treatment effect between each two treatments, also those that have never been compared in a trial, by using direct and indirect evidence [9]. NMA also allows ranking drugs by benefits and harms [10], and thus are used in clinical guidelines to support recommendations [11]. Current NMA on antiplatelet drugs for secondary prevention of stroke focused only on a limited number of treatments and did not include a thorough assessment of the confidence in the estimates [12–17]. Therefore, we conducted a NMA to summarize the current evidence for using antiplatelet drugs for secondary prevention in adult patients with ischemic stroke or TIA by estimating their relative efficacy and safety and providing a clinically useful ranking to help clinicians in their daily practice as well as informed clinical guidelines’ development.

## Methods

### Protocol and registration

The systematic review protocol, registered in PROSPERO (CRD42020159896) [18] was developed following the Preferred Reporting Items for Systematic Review and Meta-analysis Protocols (PRISMA-P) statement [19]. We used the PRISMA-network meta-analysis extension to report the results [20].

### Search strategy and selection criteria

We searched MEDLINE, EMBASE, and the Cochrane Central Register of Controlled Trials (CENTRAL) electronic databases from inception date to September 2020, with no language restrictions. The full search strategies is available on PROSPERO [18]. We searched ClinicalTrials.gov for ongoing studies (January 2016-December 2020).

### Eligibility criteria

We included randomized controlled trials (RCTs) comparing any antiplatelet drug at any dose, as monotherapy or combination therapy, with either a control (placebo/no treatment) or another antiplatelet drug at any dose, as monotherapy or combination therapy, for secondary prevention in adults (≥ 18 years old, both sexes) with ischemic stroke or TIA in which hemorrhage had been ruled out. We included all settings of care (e.g., acute or nursing homes, hospitals or ambulatory, etc.), as well as both acute and delayed treatments. We excluded RCTs comparing different doses of the same drug if no other eligible comparator was included, and those not providing outcome data on a specific antiplatelet drug. We included reports published in English or Italian. Two authors independently performed study selection and data extraction; any discrepancy was resolved by consensus and arbitration by a third author. We excluded from the statistical analyses studies with a total sample size < 100 participants because they are likely to produce biased estimated and overestimate the treatment effect [21]. This is a variation of the protocol [18].

### Outcomes

Primary outcomes were all-cause mortality and the proportion of patients who developed a stroke, irrespective of its type (ischemic or hemorrhagic) and severity. Secondary outcomes included proportion of patients who developed: ischemic stroke; ischemic stroke or TIA irrespective of severity; cardiovascular events; hemorrhagic strokes; intracranial hemorrhages; major bleeding. Supplementary 1 reports secondary outcomes definitions. We extracted outcome data at the longest available follow-up. Network plots were used to describe the network geometry [22].

### Risk of bias assessment and confidence in the evidence

One reviewer evaluated the risk of bias for included study using the criteria of The Cochrane Collaboration [23]. This appraisal was independently checked by a second reviewer. We judged the confidence in the evidence derived from NMA for primary and secondary outcomes using the web application CINeMA (http://cinema.ispm.ch/) [24]. Supplementary 1 reports the methodology for the risk of bias and confidence in the evidence assessment.

### Statistical analyses

We measured treatment effects using the odds ratio (OR) with 95 % confidence intervals (95 % CIs). For all outcomes with at least two studies, we performed standard pairwise meta-analyses of any antiplatelet drug versus placebo/no treatment with a random-effects model. We compared different antiplatelets through NMA performed under a frequentist framework using a random-effects model. Results of NMAs were presented in league tables and forest plots. For each outcome, we calculated the probability of each treatment to be the best among all treatments by using the surface under the cumulative ranking curve area (SUCRA) [10]. We presented the results from pairwise meta-analyses and NMAs as summary relative effect sizes. We reported absolute risk difference (ARD) estimates, calculated using as baseline the proportion of patients with an event in the control arm (i.e., placebo/no treatment) of the included studies, and applying the OR estimated in the NMA to compute the absolute difference between the intervention and control arms within GRADEPro [25].

We estimated heterogeneity variances for each pairwise comparison in standard pairwise meta-analyses and assess the presence of statistical heterogeneity by visually inspecting the forest plots and calculating the I-squared statistic [26]. In NMA, we assumed a common estimate for heterogeneity variance across comparisons and based our assessment of statistical heterogeneity in the whole network on the magnitude of the common heterogeneity parameter [27]. We evaluated statistical disagreement between direct and indirect effect sizes (incoherence) in local and global approaches [28]. Locally, we used the node-splitting approach [29]; we used the ‘design-by-treatment’ Q-statistic in the entire network [28]. When we found moderate heterogeneity or incoherence, we explored the impact of potential effect modifiers at study and patient-level with subgroup analyses.

We considered the following potential effect modifiers: age, gender, stroke subtypes at inclusion based on TOAST classification (cardioembolic and non-cardioembolic versus non-cardioembolic only) [30], time from the first ischemic event to randomization (< 7 days versus ≥ 7 days), and treatment duration (< 1 month versus ≥ 1 month). We performed sensitivity analyses of any antiplatelet drug versus placebo/no treatment for each primary outcome, including only trials that were classified as having a low risk of bias, and only trials in which neuroimaging was used to exclude hemorrhagic stroke at inclusion.

## Results

**Fig. 1:**
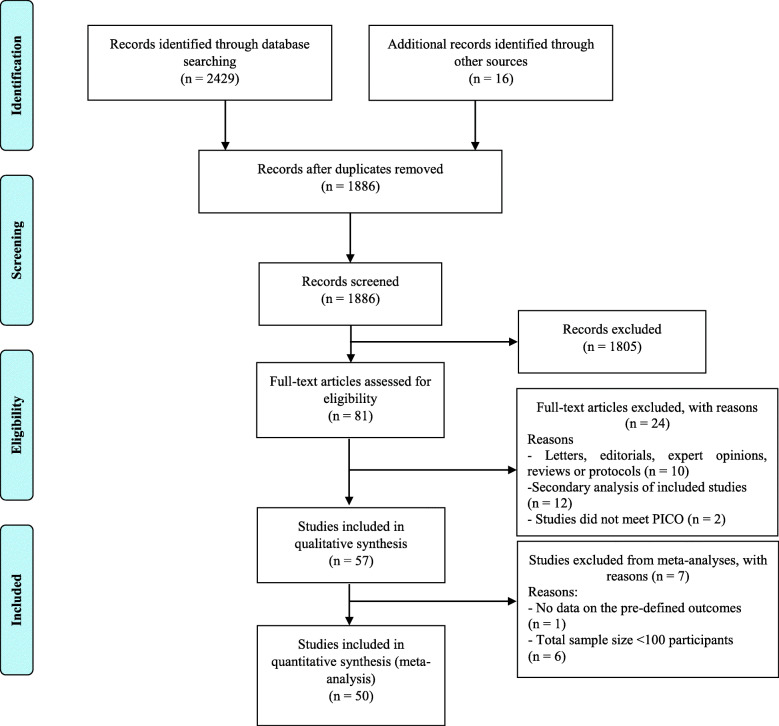
Study selection

A total of 1,886 citations were identified by the search, and 81 potentially eligible articles were retrieved in full text (Fig. 1). Overall, 57 trials were included in the review (Supplementary 2). We excluded from the statistical analysis one study that did not report any data on the pre-defined selected outcomes [31], and six studies [32–37] with a sample size < 100 participants. The remaining 50 studies (*n* = 165,533 patients) were included in our quantitative analysis. Supplementary 2 summarizes the characteristics of the 57 included trials, published between 1978 and 2020. Twenty-eight of 57 (49 %) studies randomized patients with ischemic stroke or TIA as index event, 25 (44 %) with ischemic stroke, and four (7 %) with TIA only. Thirty-nine (68 %) trials included only patients with non-cardioembolic strokes. The included studies assessed the effect of 21 different antiplatelet drugs or combinations. The median follow-up period was 18 months (range, 7 days – 4 years). Overall, 30 (53 %) trials were rated as low risk of bias, 18 (32 %) as moderate, and 9 (16 %) as high risk (Supplementary 3). We also identified four ongoing studies (Supplementary 4).

**Fig. 2:**
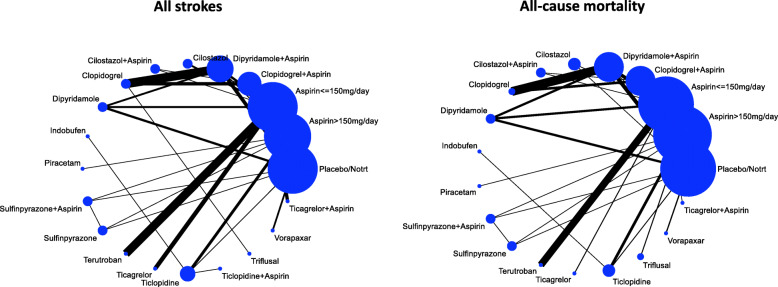
Network plots of evidence for primary outcomes: each line links the treatments that have been directly compared in studies. The thickness of the line is proportional to the precision of each direct estimate, and the width of each circle is proportional to the number of studies included in the treatment

**Fig. 3:**
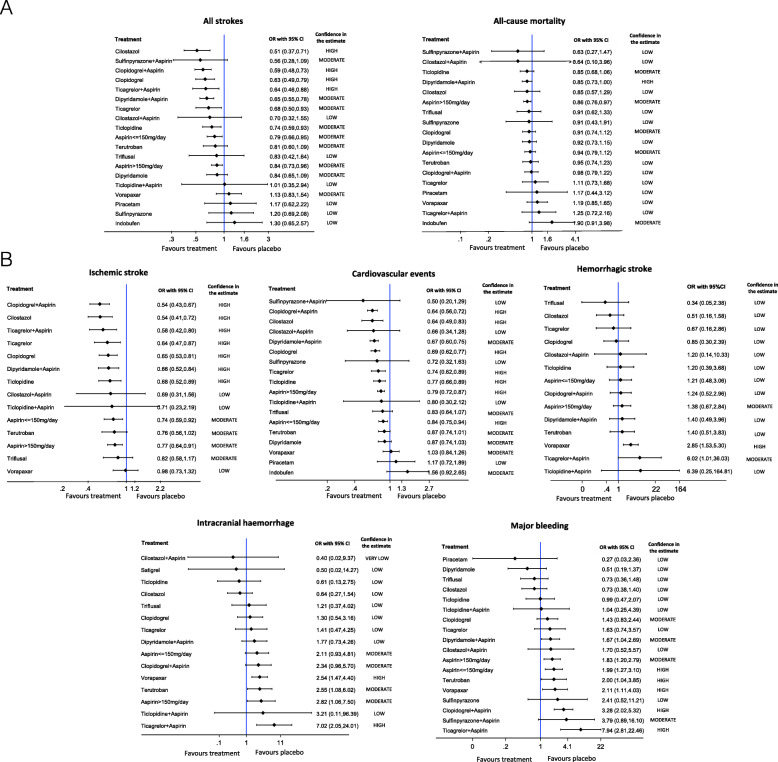
Forest plot of network estimates of primary (**A**) and secondary (**B**) outcomes of each antiplatelet/combination of antiplatelet drugs against placebo/no treatment from the network meta-analysis, with the corresponding confidence in the evidence

Figure 2 and Supplementary 5 shows the network geometry for each primary and secondary outcome, respectively. Results from pairwise meta-analysis for each primary and secondary outcome are reported in Supplementary 6. Figure 3 shows the estimates of primary and secondary outcomes of each antiplatelet/combination of antiplatelet drugs against placebo/no treatment from the NMA, with the corresponding confidence in the evidence. The league tables including results from the NMA for each treatment compared to other treatments are reported in the Supplementary excel file.

Thirty-seven RCTs (147,742 participants) addressed all stroke outcome comparing an antiplatelet drug or combination versus placebo/no treatment or another antiplatelet drug/combination. Moderate to high-confidence in evidence indicated that cilostazol, clopidogrel alone and in combination with aspirin, ticagrelor alone and in combination with aspirin, dipyridamole in combination with aspirin, ticlopidine and aspirin both ≤ 150 and > 150 mg/day were significantly associated with the greatest benefits when compared with placebo/no treatment (Fig. 3). OR estimates ranged from 0.51 (cilostazol) to 0.84 (aspirin > 150 mg/day), corresponding to ARD from 3.6 to 1.1 % fewer strokes. When compared with aspirin ≤ 150 mg/day, only clopidogrel (with high confidence), clopidogrel plus aspirin and cilostazol (with moderate confidence) significantly reduced the risk of all strokes (OR range from 0.65 to 0.79, corresponding to an ARD from 2.7 to 1.6 % fewer strokes (Supplementary 7). The common standard deviation heterogeneity estimate was 0.11.

Forty-two RCTs (154,016 participants) addressed all-cause mortality. Only aspirin > 150 mg/day significantly reduced all-cause mortality compared to placebo/no treatment (OR 0.86, 95 % CI 0.76 to 0.97; ARD 0.9 %, 95 % CI 1.5–0.2 % fewer, moderate confidence). Moderate to high-confidence in evidence indicated that ticlopidine and dipyridamole in combination with aspirin could reduce all-cause mortality, with OR estimates below 0.9 versus placebo/no treatment, although not significant (Fig. 3). No drug was significantly more effective than aspirin ≤ 150 mg/day. The common standard deviation heterogeneity estimate was 0.07.

Thirty-nine RCTs (148,414 participants) addressed ischemic stroke. Moderate to high-confidence in evidence indicated that clopidogrel alone and in combination with aspirin, cilostazol, ticagrelor alone and in combination with aspirin, dipyridamole in combination with aspirin, ticlopidine and aspirin both ≤ 150 and > 150 mg/day were significantly associated with the greatest benefits compared with placebo/no treatment (Fig. 3). OR estimates ranged from 0.54 (clopidogrel in combination with aspirin) to 0.77 (aspirin > 150 mg/day), corresponding to ARD from 2.0 to 1.0 % fewer ischemic stroke. In comparison with aspirin ≤ 150 mg/day, we found high confidence in the benefit for clopidogrel in combination with aspirin and moderate confidence for cilostazol and ticagrelor in combination with aspirin (OR range from 0.73 to 0.78, corresponding to ARD from 1.9 to 1.5 % fewer ischemic stroke) (Supplementary 7). The common standard deviation heterogeneity estimate was 0.09.

Thirty-nine RCTs (146,202 participants) addressed cardiovascular events. Moderate to high-confidence in evidence indicated that clopidogrel alone and in combination with aspirin, cilostazol, dipyridamole in combination with aspirin, ticagrelor, ticlopidine and aspirin both ≤ 150 and > 150 mg/day were significantly associated with the greatest benefits versus placebo/no treatment (Fig. 3). OR estimates ranged from 0.64 (clopidogrel in combination with aspirin) to 0.84 (aspirin ≤ 150 mg/day), corresponding to ARD from 3.3 to 1.4 % fewer cardiovascular events. In comparison with aspirin ≤ 150 mg/day, clopidogrel and dipyridamole in combination with aspirin (with high confidence), and cilostazol (with moderate confidence) were significantly more beneficial (OR range from 0.76 to 0.82, corresponding to ARD from 2.3 to 1.7 % fewer cardiovascular event), while indobufen was more harmful (OR 1.86, 95 % CI 1.09 to 3.17, ARD 7.4 %, 95 % CI 0.8–16.5 % more cardiovascular events; high confidence) (Supplementary 7). The common standard deviation heterogeneity estimate was 0.03.

Thirty-one RCTs (130,274 participants) addressed hemorrhagic stroke. No drug was significantly more effective than placebo/no treatment (Fig. 3). High-confidence in evidence indicated that cilostazol significantly reduced hemorrhagic stroke in comparison with aspirin ≤ 150 mg/day (OR 0.42 95 % CI 0.19 to 0.92, ARD 0.2 %, 95 % CI 0.3–0 % fewer hemorrhagic strokes) (Supplementary 7). Conversely, vorapaxar (with high confidence) and ticagrelor in combination with aspirin (with moderate confidence) were significantly associated with the greatest harms when compared with placebo/no treatment. OR estimates ranged from 2.85 (vorapaxar vs. placebo added to standard antiplatelet therapy) to 6.02 (ticagrelor in combination with aspirin), corresponding to ARD from 1.4 to 3.7 % more hemorrhagic strokes) (Fig. 3). When compared with aspirin ≤ 150 mg/day, ticagrelor in combination with aspirin was significantly associated with the greatest harms (OR 4.98, 95 % CI 1.08 to 23.01, ARD 1.6 %, 95 % CI 0–8.4 % more hemorrhagic strokes, with moderate confidence) (Supplementary 7). The common standard deviation heterogeneity estimate was 0.10.

Twenty-seven RCTs (106,309 participants) addressed intracranial hemorrhage. No drug was more beneficial than placebo/no treatment (Fig. 3), while cilostazol (OR 0.30 95 % CI 0.16 to 0.59, ARD 0.5 % 95 % CI 0.7–0.3 % fewer intracranial hemorrhage, high confidence) and clopidogrel (OR 0.62 95 % CI 0.39 to 0.96, ARD 0.3 % 95 % CI 0.5–0 % fewer intracranial hemorrhages, moderate confidence) were significantly more beneficial than aspirin ≤ 150 mg/day (Supplementary 7). Moderate to high-confidence in evidence indicated that vorapaxar, terutroban, aspirin > 150 mg/day and ticagrelor in combination with aspirin were significantly associated with the greatest harms versus placebo/no treatment (Fig. 3). OR estimates ranged between 2.54 (vorapaxar vs. placebo added to standard antiplatelet therapy) to 7.02 (ticagrelor in combination with aspirin), corresponding to ARD from 1.1 to 4.2 % more intracranial hemorrhages. In comparison with aspirin ≤ 150 mg/day, only ticagrelor in combination with aspirin was significantly more harmful (OR 3.32 95 % CI 1.33 to 8.28, ARD 1.8 % 95 % CI 0.3–5.3 % more intracranial hemorrhages; high confidence) (Supplementary 7). The common standard deviation heterogeneity estimate was almost null.

Forty RCTs (146,826 participants) addressed major bleeding. No drug significantly reduced the risk of major bleeding when compared with placebo/no treatment (Fig. 3). High-confidence in evidence indicated that dipyridamole, cilostazol and triflusal significantly reduced the risk of the outcome in comparison with aspirin ≤ 150 mg/day. OR estimates ranged between 0.25 (dipyridamole) to 0.37 (cilostazol and triflusal), corresponding to ARD from 1.0 to 0.9 % fewer events of major bleeding (Supplementary 7). Moderate to high-confidence in evidence indicated that dipyridamole in combination with aspirin, aspirin both ≤ 150 and > 150 mg/day, terutroban, vorapaxar, clopidogrel in combination with aspirin and ticagrelor in combination with aspirin were significantly associated with the greatest harms versus placebo/no treatment. OR estimates ranged from 1.67 (clopidogrel in combination with aspirin) to 7.94 (ticagrelor in combination with aspirin), corresponding to ARD from 0.8 to 7.5 % more events of major bleeding (Fig. 3). In comparison with aspirin ≤ 150 mg/day, clopidogrel in combination with aspirin (OR 1.65 95 % CI 1.15 to 2.37, ARD 0.9 % more 95 % CI 0.2–1.8 % more; high confidence) and ticagrelor in combination with aspirin (OR 3.99 95 % CI 1.56 to 10.22, ARD 3.9 % more 95 % CI 0.8–11.2 % more events of major bleeding; high confidence) were significantly associated with the greatest harms (Supplementary 7). The common standard deviation heterogeneity estimate was 0.23.

We did not perform NMA for ischemic stroke or TIA outcome as it was assessed only by 11 RCTs (17,342 participants) and only two comparisons had at least two studies. This resulted in a disconnected network (see the network plot in Supplementary 5). Results from standard pairwise meta-analysis are available in Supplementary 6.

SUCRA, absolute probability to be the best treatment, and the mean rank are reported in Supplementary 8. Results from the assessment of incoherence are showed in Supplementary 9.

We compared the magnitude of the heterogeneity estimates with the empirical distributions of heterogeneity values derived by Turner [27] for objective (all-cause mortality only) and semi-objective outcomes, for comparisons of treatments versus placebo. Our heterogeneity estimates, except for major bleeding, were below the median of the empirical distributions of heterogeneity values. Therefore, given the small heterogeneity and little incoherence for all outcomes except those for major bleeding, subgroup analyses (stroke subtypes; time from first ischemic event to randomization; and treatment duration) were performed for the major bleeding outcome only (see results in Supplementary excel file). Subgroup analyses for age and gender were not performed because of scarce available data. Results from the sensitivity analyses for primary outcomes are also reported in Supplementary excel file. The comparison-adjusted funnel plots for each outcome are reported in Supplementary 10.

## Discussion

In this systematic review and NMA we found that cilostazol, clopidogrel alone and in combination with aspirin, ticagrelor alone and in combination with aspirin, dipyridamole in combination with aspirin, ticlopidine, and aspirin both ≤ 150 and > 150 mg/day are significantly associated with the greatest benefits in terms of recurrent stroke (both ischemic and hemorrhagic considered together, and ischemic alone) and cardiovascular event risk reduction compared to placebo/no treatment. The absolute risk reductions range between 1.1 and 3.6 % for recurrent strokes and between 1.4 and 3.3 % for cardiovascular events. However, ticagrelor in combination with aspirin is significantly associated with the greatest harms in terms of increased risk of hemorrhagic stroke (absolute risk increase of 3.7 %), intracranial hemorrhage (absolute risk increase of 4.2 %), and major bleeding (absolute risk increase of 7.5 %) compared to placebo/no treatment. Furthermore, clopidogrel in combination with aspirin significantly increases the risk of major bleeding, with an absolute risk increase of 0.8 %. Aspirin > 150 mg/day compared to aspirin ≤ 150 mg/day and combination therapies with aspirin vs. corresponding monotherapies, do not show consistent increased benefits while possible increased harms. Thus, considering the benefits and harms ratio, cilostazol, clopidogrel, ticagrelor and ticlopidine alone, aspirin ≤ 150 mg/day, and dipyridamole in combination with aspirin, appear to be the best antiplatelet drugs for secondary prevention of patients with ischemic stroke or TIA. Low-dose aspirin is the most widely used first-line antiplatelet drug for secondary prevention of ischemic stroke, given the large body of evidence supporting its efficacy in reducing the risk of stroke, cardiovascular events, and certain cancers [38, 8]. Thus, we also explored the relative effect of antiplatelet drugs compared to aspirin ≤ 150 mg/day. This NMA suggests that clopidogrel significantly reduces the risk of all strokes, cardiovascular event and intracranial haemorrhage outcomes, and cilostazol provide advantages on all outcomes, except for all-cause mortality, over aspirin ≤ 150 mg/day. However, all the studies that evaluated cilostazol involved Asian population, thus limiting the generalizability of the results to the Caucasic population. Indeed, the clinical use of cilostazol for stroke prevention is limited to the Asia-Pacific countries, while in the US and Europe it is approved for intermittent claudication only. In our NMA, antiplatelet drugs do not seem to modify all cause-mortality. This effect may be due to a genuine result or to a lack of sufficient precision of the estimates, as suggested by the GRADE assessment. A possible exception could be aspirin > 150 mg/day that is associated with a small decrease of all-cause mortality. However, this effect was not significant when only studies at low risk of bias are considered. Nevertheless, if any, the effect of aspirin on mortality can be due to mechanisms other than its specific antiplatelet function, for instance its effect on cancer mortality [38].

### Comparison with other studies

To our knowledge, the first NMA on antiplatelet drugs used after TIA or stroke was published in 2008 and included 24 studies, involving more than 42,000 participants [39]. The combination of aspirin and dipyridamole emerged as the most effective regimen in preventing vascular events. Six further NMAs were published by Chinese authors between 2015 and 2019 [12–17]. Although minor differences in terms of included studies, five reviews agreed on suggesting that cilostazol is the most effective drug. One NMA concluded in favor of clopidogrel and aspirin and their combination [16].

Overall, our NMA confirms these findings, providing a relevant update up to 2020. While Xiang et al. concluded that further studies are needed to confirm their findings, [14] we found high confidence on the efficacy of cilostazol and clopidogrel. According to the GRADE methodology, this means that it is unlikely that further research will change the direction and magnitude of these estimates.

Our NMA includes 50 RCTs providing data useful for the NMA (over 165,000 participants) compared to the previous systematic reviews that included from 24 [15] to 45 [14] studies.

Moreover, it assessed the effect on several outcomes of promising interventions such as ticagrelor, that has been recently approved by the FDA to reduce the risk of stroke in patients with acute ischemic stroke or high-risk TIA [40]. A recent NMA – published only as abstract – also assessed ticagrelor suggesting that as monotherapy it has a similar efficacy and safety profile than clopidogrel [41]. We included a thorough assessment of the confidence of estimates, i.e., that the measured effects are correct or adequate to support a particular decision or recommendation [42], according to the GRADE approach for NMA. This framework includes the assessment of the classical five dimensions (within-study bias, reporting bias, indirectness, imprecision, and heterogeneity) considering the complexity of the network. Moreover, it includes the assessment of the incoherence, that is the statistical manifestation of intransitivity, the measure of the disagreement from direct and indirect evidence [43]. We also found small heterogeneity for all outcomes, except for major bleeding for which subgroup analyses showed robust results compared to the overall.

### Limitations of this review

Our study has some limitations. First: many direct treatment comparisons in our network were based on one study only. Second: most of the treatments included in our network were compared with placebo/no treatment or aspirin only and the number of comparisons with an active drug versus another active drug was quite small; this means that our evidence was often a result of an indirect evidence only. Third, our NMA, principally based on trials including only patients with non-cardioembolic ischemic strokes, cannot inform the use of antiplatelet drugs in different stroke subtypes that are associated to variable pattern of stroke recurrence [44]. Fourth: subgroup data were not adequate in terms of number of studies and treatment included to assess treatment effects in the acute phase. Current guidelines recommend dual antiplatelet therapy in the first 90 days after ischemic stroke or TIA [6] however, in our NMA, only 12 studies limit the duration of follow-up to the first 3 months after the acute event. Therefore, our conclusions should be limited to long-term secondary prevention only.

## Conclusions

In conclusion, cilostazol, clopidogrel, ticagrelor and ticlopidine alone, aspirin ≤ 150 mg/day, and dipyridamole in combination with aspirin, appear to be the best antiplatelet drugs for secondary prevention of patients with ischemic stroke or TIA. The choice of drug will take into account the characteristics of the individual patient, for example ethnicity (data on cilostazol are limited to the Asian population), allergies and comorbidities (thienopyridines are not indicated in patients with severe liver disease). This up-to-date, comprehensive and accurate assessment of the effectiveness and safety of a broad spectrum of treatments may be useful to support appropriate choices, both at individual and public health level, informing drug prescribing and procurement.

## Supplementary Information

**Additional file 1: Supplementary 1.** Methodology details: secondary outcomes definition, study risk of bias and confidence in the evidence assessments. **Supplementary 2.** Characteristics of included studies table and references. **Supplementary 3.** Risk of bias summary table. **Supplementary 4.** Table of ongoing studies (last update December 2020). **Supplementary 5.** Network plots of evidence for secondary outcomes. **Supplementary 6.** Results of the pairwise meta-analyses for primary and secondary outcomes. **Supplementary 7.** Assessment of the confidence in the network estimates of each drug versus placebo/no treatment and versus aspirin ≤150 mg/day by outcome. **Supplementary 8.** SUCRA, probability to be the best and mean rank by outcome. **Supplementary 9.** Results from the assessment of incoherence by using global and local (side-split method) approaches for primary and secondary outcomes. **Supplementary 10.** Comparison-adjusted funnel plot for a network of interventions by outcome.

**Additional file 2.** League tables from the main analysis for primary and secondary outcomes, from the subgroup analysis for major bleeding and from the sensitivity analysis for primary outcomes. Treatments are reported in the diagonal. Results are reported as odds ratio and relative 95% confidence interval. Comparisons must be read from left to right. On the triangle under the diagonal, an estimate less than 1 favor the column-defining treatment. On the triangle upper the diagonal, an estimate more than 1 favor the row-defining treatment. Significant results are in bold (only in the lower triangle).

## Data Availability

The full dataset and statistical code will be available upon reasonable request.

## Abbreviations

ARD: Absolute risk difference
CENTRAL: Cochrane Central Register of Controlled Trials
CI: Confidence interval
GRADE: Grades of Recommendation, Assessment, Development and Evaluation
NMA: Network meta-analysis
OR: Odds ratio
PRISMA: Preferred reporting items for systematic review and meta-analysis
RCT: Randomized controlled trial
SUCRA: Surface under the cumulative ranking curve area
TIA: Transient ischemic attack
