# Supplementary material for: Antiplatelet drugs for secondary prevention in patients with ischemic stroke or transient ischemic attack: a systematic review and network meta-analysis
Source: BMC Neurol. 2021 Aug 16;21:319. doi: 10.1186/s12883-021-02341-2 (PMC8365925; doi:10.1186/s12883-021-02341-2)
Supplement: Supplementary file 1 — Additional file 1: Supplementary 1. Methodology details: secondary outcomes definition, study risk of bias and confidence in the evidence assessments. Supplementary 2. Characteristics of included studies table and references. Supplementary 3. Risk of bias summary table. Supplementary 4. Table of ongoing studies (last update December 2020). Supplementary 5. Network plots of evidence for secondary outcomes. Supplementary 6. Results of the pairwise meta-analyses for primary and secondary outcomes. Supplementary 7. Assessment of the confidence in the network estimates of each drug versus placebo/no treatment and versus aspirin ≤150 mg/day by outcome. Supplementary 8. SUCRA, probability to be the best and mean rank by outcome. Supplementary 9. Results from the assessment of incoherence by using global and local (side-split method) approaches for primary and secondary outcomes. Supplementary 10. Comparison-adjusted funnel plot for a network of interventions by outcome. [file 12883_2021_2341_MOESM1_ESM.docx]

**SUPPLEMENTAL MATERIAL**

[**Supplementary 1. Methodology details: secondary outcomes definition, study risk of bias and confidence in the evidence assessments** 4](#_Toc66111256)

[*Secondary outcome definitions* 4](#_Toc66111257)

[*Study risk of bias assessment* 4](#_Toc66111258)

[*Confidence in the evidence from network meta-analysis assessments* 4](#_Toc66111259)

[*Within-study bias* 4](#_Toc66111260)

[*Indirectness* 5](#_Toc66111261)

[*Imprecision, heterogeneity, and incoherence* 5](#_Toc66111262)

[**Supplementary 2. Characteristics of included studies table (N=57)** 7](#_Toc66111263)

[*References* 14](#_Toc66111264)

[**Supplementary 3. Risk of bias summary table** 17](#_Toc66111265)

[*References* 35](#_Toc66111266)

[**Supplementary 4. Table of ongoing studies (last update December 2020)** 36](#_Toc66111267)

[**Supplementary 5. Network plots of evidence for secondary outcomes** 37](#_Toc66111268)

[*Ischemic stroke* 37](#_Toc66111269)

[*Ischemic stroke or transient ischemic attack* 38](#_Toc66111270)

[*Cardiovascular event* 39](#_Toc66111271)

[*Hemorrhagic stroke* 40](#_Toc66111272)

[*Intracranial haemorrhage* 41](#_Toc66111273)

[*Major bleeding* 42](#_Toc66111274)

[**Supplementary 6. Results of the pairwise meta-analyses for primary and secondary outcomes** 43](#_Toc66111275)

[*All strokes* 43](#_Toc66111276)

[*All-cause mortality* 49](#_Toc66111277)

[*Ischemic stroke* 55](#_Toc66111278)

[*Ischemic stroke or transient ischemic attack* 60](#_Toc66111279)

[*Cardiovascular event* 62](#_Toc66111280)

[*Hemorrhagic stroke* 68](#_Toc66111281)

[*Intracranial haemorrhage* 72](#_Toc66111282)

[*Major bleeding* 76](#_Toc66111283)

[**Supplementary 7. Assessment of the confidence in the network estimates by outcome** 83](#_Toc66111284)

[*All strokes* 83](#_Toc66111285)

[*All-cause mortality* 87](#_Toc66111286)

[*Ischemic stroke* 90](#_Toc66111287)

[*Cardiovascular event* 93](#_Toc66111288)

[*Hemorrhagic stroke* 96](#_Toc66111289)

[*Intracranial haemorrhage* 99](#_Toc66111290)

[*Major bleeding* 102](#_Toc66111291)

[**Supplementary 8. SUCRA, probability to be the best and mean rank by outcome** 105](#_Toc66111292)

[*All strokes* 105](#_Toc66111293)

[*All-cause mortality* 105](#_Toc66111294)

[*Ischemic stroke* 106](#_Toc66111295)

[*Cardiovascular event* 107](#_Toc66111296)

[*Hemorrhagic stroke* 107](#_Toc66111297)

[*Intracranial haemorrhage* 108](#_Toc66111298)

[*Major bleeding* 108](#_Toc66111299)

[**Supplementary 9. Results from the assessment of incoherence by using global and local (side-split method) approaches for primary and secondary outcomes** 110](#_Toc66111300)

[*All strokes* 110](#_Toc66111301)

[*All-cause mortality* 111](#_Toc66111302)

[*Ischemic stroke* 112](#_Toc66111303)

[*Cardiovascular event* 113](#_Toc66111304)

[*Hemorrhagic stroke* 114](#_Toc66111305)

[*Intracranial haemorrhage* 115](#_Toc66111306)

[*Major bleeding* 116](#_Toc66111307)

[**Supplementary 10. Comparison-adjusted funnel plot for a network of interventions by outcome** 118](#_Toc66111308)

[*All strokes* 118](#_Toc66111309)

[*All-cause mortality* 119](#_Toc66111310)

[*Ischemic stroke* 120](#_Toc66111311)

[*Cardiovascular event* 121](#_Toc66111312)

[*Hemorrhagic stroke* 122](#_Toc66111313)

[*Intracranial haemorrhage* 123](#_Toc66111314)

[*Major bleeding* 124](#_Toc66111315)

[*Reference* 124](#_Toc66111316)

# **Supplementary 1. Methodology details: secondary outcomes definition, study risk of bias and confidence in the evidence assessments**

# *Secondary outcome definitions*

- cardiovascular event: any sudden death, fatal or non-fatal acute coronary syndrome, stroke, intracranial hemorrhage, or pulmonary embolism
- hemorrhagic stroke: an acute extravasation of blood into and around the brain parenchyma (intracerebral hemorrhage and subarachnoid hemorrhage, excluding subdural hematoma and epidural hematoma)
- intracranial hemorrhage: includes hemorrhagic stroke, subdural hematoma and epidural hematoma
- major bleeding: including all symptomatic intracranial hemorrhage, any fatal bleeding, or any bleeding requiring hospital admission (i.e. for transfusion of unit of red-blood cells) or leaving significantly disability (with persistent sequelae).

# *Study risk of bias assessment*

The following domains of bias were considered: selection (random sequence generation, allocation concealment), performance (blinding of participants and personnel), detection (blinding of outcome assessment), attrition (incomplete outcome data), and selective outcome reporting. We explicitly judged the risk of bias in each criterion as ’low’, ’high’, or ’unclear’. We evaluated incomplete outcome data as having a low risk of bias when the numbers and reasons for dropouts were balanced (i.e., in the absence of a significant difference) between arms. Our assessment of methodological quality included published trial protocols when available. Finally, for each study, we explicitly also judged the overall risk of bias as follows: we considered allocation concealment, blinding of participants and personnel, and incomplete outcome data to classify each study as having low risk of bias when we judged all of the selected criteria as having low risk of bias; high risk of bias when we judged at least one criterion among those selected as having high risk of bias; and moderate risk of bias in the remaining cases.

# *Confidence in the evidence from network meta-analysis assessments*

We assessed the following items: within-study bias, indirectness, imprecision, heterogeneity, incoherence and reporting bias. For each item, we judged whether we had no concerns, some concerns, or major concerns. By considering the judgments from all items, we derived an overall confidence rating for each comparison. We assessed the confidence in the network estimates as at very low, low, moderate, or high confidence. We used the web application CINeMA (https://cinema.ispm.unibe.ch/) and derived the judgment for each item and the overall confidence rating for each network estimate as described by Papakonstantinou and colleagues.^1^

# *Within-study bias*

The assessment of the within-study bias is showed in the risk of bias bar chart. The chart is automatically generated in CINeMA by using the overall study risk of bias (see Supplementary 3) and the study contribution matrix (i.e., how much each direct evidence contributes to each the network estimate) also calculated in CINeMA. Each bar shows how much studies at low risk of bias (green), moderate (yellow) or high risk of bias (red) contribute in percentage to the network estimate. We used the “majority risk of bias” to summarize risk of bias across contributions for each network estimate and derive the judgment for within-study bias.

# *Indirectness*

We used the “majority” criterion to assess the indirectness and summarized it with bar charts. We assigned low risk for indirectness to all included studies and hence we judged all comparisons as with “no concerns” for indirectness (indirectness bar chart not reported).

# *Imprecision, heterogeneity, and incoherence*

For the assessment of imprecision, heterogeneity, and incoherence we considered an odds ratio of 0.90 as clinically important for all-cause mortality and of 0.80 for other outcomes and followed the CINeMA criteria.^1^ We assessed the level of concern as reported in the figure below^2^:


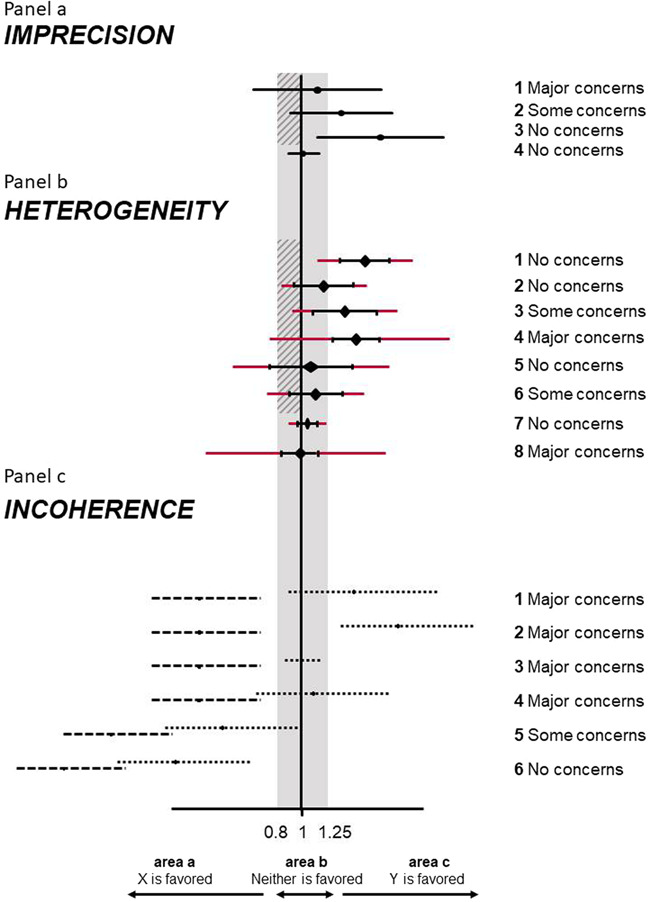


Legend: Illustration of rules to assess imprecision (a), heterogeneity (b), and incoherence (c) in CINeMA. We assume several fictional scenarios for the odds ratio from NMA comparing interventions X and Y. The clinically important effects were set at 0.8 and 1.25 ( = 1/0.8). The gray areas represent values that favor neither of the competing interventions. The shaded interval represents the interval between the null effect and clinically important size of effect. Black horizontal lines indicate confidence intervals and red extensions indicate prediction intervals of NMA relative treatment effects. Dotted lines represent direct and dashed lines represent indirect confidence intervals. Judgments are the same for cases symmetrical to those illustrated. NMA, network meta-analysis

For judging the confidence in the estimates for imprecision and for heterogeneity, odds ratio with relative 95% confidence interval and prediction intervals are reported in the forest plots below.

The judgments of the confidence in the estimates for incoherence are based on the results from the design-by-treatment interaction test and side-splitting test (see Supplementary 9).

*Reporting bias*

The comparison-adjusted funnel plots by outcome are reported in Supplementary 9. For each outcome, we judged all estimates as undetected.

*Confidence rating*

We rated:

- high confidence if no concerns in all the above-mentioned domains
- moderate confidence if at least a “some concerns” but no “major concerns”
- low confidence if one major concerns
- very low confidence if more than one major concerns

*References*

^1^Papakonstantinou T, Nikolakopoulou A, Higgins JPT, Egger M, Salanti G. CINeMA: Software for semiautomated assessment of the confidence in the results of network meta-analysis. Campbell Systematic Reviews. 2020;16:e1080.

^2^Campbell Systematic Reviews, Volume: 16, Issue: 1, First published: 11 March 2020, DOI: (10.1002/cl2.1080)

# **Supplementary 2. Characteristics of included studies table (N=57)**

| **Study** | **Year, Country** | **Index event (n pts random)** | **Follow up** | **NI at entry** | **Non-CE only** | **TR** | **Age (mean, y)** | **Males (%)** | **Intervention(s)** | **Control** |
| --- | --- | --- | --- | --- | --- | --- | --- | --- | --- | --- |
| THALES* ^1^ | 2020, global | Ischemic stroke or TIA (11016) | 2 mo | Yes | No | ≤24 h | 65 | 39 | Ticagrelor (180 mg loading dose followed by 90 mg twice daily) + aspirin (300 to 325 mg on day 1 followed by 75 to 100 mg daily) | Aspirin (300 to 325 mg on day 1 followed by 75 to 100 mg daily) |
| Khazaei^§2^ | 2019, Iran | Ischemic stroke or TIA (54) | 3 mo | Optional | Yes | NR | 67 | 63 | Aspirin 80 mg/day + Clopidogrel 75 mg/day | Aspirin 80 mg/day |
| PICASSO study^3^ | 2018, Asia | Ischemic stroke or TIA (1534) | Median 1.9 y | Yes | Yes | <180 days | 66 | 62 | Cilostazol 100 mg twice daily | Aspirin 100 mg daily |
| POINT trial^4^ | 2018, global | Ischemic stroke or TIA (4881) | 3 mo | Yes | Yes | ≤12 h | NR (median 65) | 55 | Clopidogrel 75 mg daily + Aspirin ranged from 50 mg to 325 mg daily - Median 81 mg | Aspirin (50 to 325 mg daily) - Median 81 mg |
| MAESTRO Study^5^ | 2017, Korea | Ischemic stroke (795) | Median 2.7 y | Yes | Yes | ≤1 mo | 61 | 68 | Triflusal 300 mg twice per day | Clopidogrel 75 mg once daily |
| COMPRESS*^6^ | 2016, Korea | Ischemic stroke (358) | 1 mo | Yes | Yes | ≤48 h | 66 | 64 | Clopidogrel 75 mg once daily + Aspirin 100 mg | Aspirin 100 mg once daily |
| SOCRATES Study^7^ | 2016, global | Ischemic stroke or TIA (13199) | 3 mo | Yes | No | ≤24 h | 66 | 59 | Ticagrelor 90 mg twice daily | Aspirin 100 mg daily |
| CATHARSIS^8^ | 2015, Japan | Ischemic stroke (165) | mean  2 y | Yes | Yes | 2 wk - 6 mo | 68 | 66 | Cilostazol 200 mg daily + Aspirin 100 mg daily | Aspirin 100 mg daily |
| Wang^9^ | 2015, China | Ischemic stroke (570) | 1 mo | Yes | Yes | <48 h | 70 | 55 | Clopidogrel 75 mg + Aspirin 200 mg daily | Aspirin 200 mg daily |
| Yi*^10^ | 2014, China | Ischemic stroke (574) | 1 mo | Yes | Yes | ≤48 h | 70 | 55 | 200 mg aspirin and 75 mg clopidogrel daily | Aspirin 200 mg/day for 30 days |
| CHANCE trial^11^ | 2013, China | Ischemic stroke or TIA (5170) | 3 mo | Yes | Yes | ≤24 h | NR (median 62) | 66 | Clopidogrel 300 mg day 1 followed by 75 mg daily for 90 days + Aspirin 75 mg to 300 mg day 1, followed by 75 mg daily for the first 21 days | Aspirin 75 mg to 300 mg day 1, followed by 75 mg daily |
| ECLIPse Study^12^ | 2013, Korea | Ischemic stroke (203) | 3 mo | Yes | Yes | ≤1 wk | 65 | 75 | Cilostazol 100 mg twice daily + Aspirin 100 mg daily | Aspirin 100 mg daily |
| TIMI 50 trial^13^ | 2013, global | Ischemic stroke (4883) | 3 y | NR | Yes | 2 wk - 12 mo | 65 | 67 | Vorapaxar 2.5 mg daily added to standard antiplatelet therapy | Placebo added to standard antiplatelet therapy |
| Nakamura^§14^ | 2012, Japan | Ischemic stroke (76) | 6 mo | Yes | Yes | ≤48 h | 67 | 74 | Cilostazol 100 mg twice + Aspirin 100 mg daily | Aspirin 100 mg daily |
| Shinohara^§15^ | 2012, Japan | Ischemic stroke (90) | 2 mo | Yes | Yes | 2 wk - 1 year | 65 | 77 | Vorapaxar 1 mg daily+ Aspirin 75 or 150 mg per day; Vorapaxar 2.5 mg daily+ Aspirin 7 ranging between 75 and 150 mg per day; | Aspirin (75 - 150 mg daily |
| SPS3 (Secondary Prevention of Small Subcortical Strokes)^16^ | 2012, global | Ischemic stroke or TIA (3020) | Mean 3.4 y | Yes | Yes | ≤6 mo | 63 | 63 | Clopidogrel 75 mg + Aspirin 325 mg daily | Aspirin 325 mg daily |
| CAIST trial^17^ | 2011, Korea | Ischemic stroke (458) | 3 mo | Yes | Yes | ≤48 hours | 63 | 61 | Cilostazol 200 mg daily | Aspirin 300 mg daily |
| CHARISMA Study^18^ | 2011, global | Ischemic stroke or TIA (4320) | median 25 mo | NR | No | ≤5 years | 65 | 63 | Clopidogrel 75 mg/day+Aspirin 100 mg daily | Aspirin 100 mg daily |
| JASAP Study^19^ | 2011, Japan | Ischemic stroke (1295) | 24 mo | Yes | Yes | 1wk - 6 mo | 66 | 72 | Dypiridamole 200 mg + Aspirin 25 mg twice daily | Aspirin 81 mg once daily |
| PERFORM study^20^ | 2011, global | Ischemic stroke or TIA (19120) | Mean 28.3 mo | Yes | Yes | 48 h – 3 mo (stroke); 8 days (TIA) | 67 | 63 | Terutroban 30 mg daily | Aspirin 100 mg daily |
| TOSS-2^21^ | 2011, Asia | Ischemic stroke (457) | 7 mo | Yes | Yes | ≤2 wk | 65 | 51 | cilostazol 100 mg twice a day + aspirin 75-150 mg once daily | clopidogrel 75 mg daily + aspirin 75-150 mg once daily |
| CSPS-2^22^ | 2010, Japan | Ischemic stroke or TIA (2757) | Mean 29 mo | Yes | Yes | ≤26 wk | 63 | 72 | Cilostazol 100 mg twice daily | Aspirin 81 mg once daily |
| Guo^§23^ | 2009, China | Ischemic stroke (68) | 12 mo | Yes | Yes | 1 - 6 mo | 61 | 35 | Cilostazol 100 mg twice daily | Aspirin 100 mg daily |
| Uchiyama^24^ | 2009, Japan | Ischemic stroke (1869) | 12 mo | Yes | No | >8 days | 64 | 71 | Clopidogrel 75 mg once daily | Ticlopidine 200 mg once daily |
| CASISP study^25^ | 2008, China | Ischemic stroke (720) | 12-18 mo | Yes | Yes | 1 - 6 mo | 60 | 69 | Cilostazol 200 mg/day | Aspirin 100 mg daily |
| Fukuuchi^26^ | 2008, Japan | Ischemic stroke (1151) | 12 mo | Yes | Yes | >8 days | 65 | 73 | Clopidogrel 75 mg once daily | Ticlopidine 200 mg once daily |
| PRoFESS^27^ | 2008, global | Ischemic stroke or TIA (20332) | Mean 2.5 y | Yes | Yes | ≤4 mo | 66 | 64 | Aspirin 25 mg + Dipyridamole 200 mg twice daily | Clopidogrel 75 mg daily |
| ESPRIT^28^ | 2006, global | Ischemic stroke or TIA (2739) | Mean 3.5 y | Yes | Yes | ≤6 mo | 63 | 65 | Aspirin (30-325 mg daily) +dipyridamole 200mg daily | Aspirin (30-325 mg daily) - Median dose 75 mg |
| CARESS trial*^29^ | 2005, Europe | Ischemic stroke or TIA (107) | 7 days | Yes | Yes | ≤3 mo | 65 | 69 | Day 1: Clopidogrel 300 mg daily; From day 2 to day 7 Clopidogrel 75 mg + Aspirin 75 mg daily | From day 1 to day 7Aspirin 75 mg once a day |
| Chairangsarit^§30^ | 2005, Thailand | Ischemic stroke (38) | 6 mo | Yes | Yes | ≤48 h | 64 | 53 | Aspirin 300 mg daily+ dipyridamole 75 mg three times per day | Aspirin 300 mg daily |
| MATCH^31^ | 2004, global | Ischemic stroke or TIA (7599) | 18 mo | Yes | Yes | ≤3 mo | 66 | 63 | 75 mg Aspirin once daily+ 75 mg Clopidogrel once daily | 75 mg Clopidogrel once daily |
| TAPIRSS Study^32^ | 2004, Argentina | Ischemic stroke or TIA (431) | Mean 1.6 y | Yes | Yes | < 6 mo | 65 | 68 | Triflusal 600 mg daily | Aspirin 325 mg daily |
| AAASPS (Aspirin and Ticlopidine for prevention of recurrent stroke in black pts)^33^ | 2003, USA | Ischemic stroke (1809) | Mean 1.5 y | Yes | Yes | 1 wk - 3 mo | 61 | 46 | Ticlopidine 250 mg twice a day | Aspirin 325 mg twice a day |
| TACIP study^34^ | 2003, Spain  Portugal | Ischemic stroke or TIA (2113) | Mean 30.1 mo | Yes | Yes | ≤6 mo | 65 | 66 | Triflusal 600 mg daily | Aspirin 325 mg daily |
| TOPALS study^35^ | 2003, Japan | Ischemic stroke or TIA (276) | Mean 1.59 y | NR | Yes | 1 - 6 mo (3 mo for TIA) | 67 | 65 | Ticlopidine 200 mg daily | Ticlopidine 100 mg + Aspirin 81 mg daily |
| Gotoh^36^ | 2000, Japan | Ischemic stroke (1095) | At least 12 mo | Yes | Yes | 1 - 6 mo | 65 | 66 | Cilostazol 100 mg twice daily | Placebo |
| Grotemeyer^37^ | 2000, Germany | Ischemic stroke (563) | 24 mo | Yes | Yes | 8 - 16 wk | 58 | 68 | Piracetam 1600 mg three times daily | Aspirin 200 mg three times daily |
| CAST* (Chinese Acute Stroke Trial)^38^ | 1997, China | Ischemic stroke (21106) | 1 mo | Yes | No | ≤48 h | 63 | 63 | Aspirin 160 mg once daily | Placebo |
| TISS (Ticlopidine Indobufen Stroke Study)^39^ | 1997, Italy | Ischemic stroke or TIA (1632) | 12 mo | Yes | No | ≤1 mo | 65 | 63 | Ticlopidine 250 mg once or twice daily | Indobufen 200 mg once or twice daily |
| CAPRIE^40^ | 1996, global | Ischemic stroke or TIA (6431) | Mean 1.9 mo | Yes | Yes | 1 wk - 6 mo | 65 | 64 | Clopidogrel 75mg once daily | Aspirin 325 mg once daily |
| European Stroke Prevention Study 2^41^ | 1996, Europe | Ischemic stroke or TIA (6602) | 2 y | Optional | No | ≤3 mo | 67 | 58 | Aspirin 25 mg twice a day; Dipyidamole 200 mg twice a day; Aspirin 25 mg+Dipyridamole 200mg twice a day | Placebo |
| JETS-1^42^ | 1995, Japan | TIA (227) | 3-6 mo | Yes | No | ≤12 wk | 61 | 69 | Satigrel 1 mg twice daily; Satigrel 2 mg twice daily | Aspirin 81 mg four time daily |
| MAST-I*^43^ | 1995, Europe | Ischemic stroke (309) | 10 days | Yes | No | ≤6 h | NR | 53 | Aspirin 300 mg daily | No treatment |
| Smirne^#44^ | 1994, Italy | TIA (183) | 6 mo | Yes | Yes | ≤3 mo | 66 | 58 | Triflusal 600 mg daily | Aspirin 300 mg daily |
| EAFT Study^45^ | 1993, Europe/  Israel | Ischemic stroke or TIA (782) | Mean 2.3 y | Yes | No | < 3 mo | 73 | 56 | Aspirin 300 mg daily | Placebo |
| De Falco*^§46^ | 1991, Italy | Ischemic stroke or TIA (87) | 15 mo | Optional | No | 1 - 4 wk | 64 | 67 | Picodamide 600 mg die | Aspirin 300 mg die |
| SALT Study^47^ | 1991, Sweden | Ischemic stroke or TIA (1360) | Median 32 mo | Optional (recommended for minor stroke) | Yes | <3 mo | 67 | NR | Aspirin 75 mg daily | Placebo |
| UK-TIA Study^48^ | 1991, UK | Ischemic stroke or TIA (2436) | At least 12 mo, mean 4 y | Optional | Yes | NR | 60 | 73 | Aspirin 600 mg twice daily; Aspirin 300 mg once daily | Placebo |
| CATS (Canadian American Ticlopidine study)^49^ | 1989, North America | Ischemic stroke or TIA  (1053) | 3 y (mean 2 y) | Yes | Yes | 1 wk - 4 mo | 65 | 62 | Ticlopidine 125 mg twice a day | Placebo |
| Ticlopidine Aspirin Stroke Study^50^ | 1989, North America | Ischemic stroke or TIA (3069) | 2-6 y | No | No | ≤3 mo | 63 | 65 | Ticlopidine 250 mg twice a day | Aspirin 650 mg twice a day |
| ESPS Study^51^ | 1987, Europe | Ischemic stroke or TIA (2500) | 24 mo | Optional | No | ≤3 mo | 63 | 58 | Dypiridamole 75 mg + Aspirin 330 mg 3 times daily | Placebo |
| Swedish Cooperative Study^52^ | 1987, Sweden | Ischemic stroke (505) | 2 y | Yes | No | 1-3 wk | 68 | 62 | Aspirin 1.5 g/day | Placebo |
| Persantine Aspirin Trial^53^ | 1985, North America | Ischemic stroke (890) | Median 25 mo | NR | No | ≤3 mo | 63 | 67 | Aspirin 325 mg+ Dypiridamole 75 mg 4 times daily | Aspirin 325 mg 4 times daily |
| Tohgi^54^ | 1984, Japan | TIA (340) | 12 mo | NR | No | ≤3 mo | NR | NR | Ticlpidine 100 mg twice | Aspirin 500 mg once |
| AICLA study^55^ | 1983, France | Ischemic stroke or TIA (604) | 3 y | No | Yes | ≤1 year | 63 | 70 | Aspirin 330 mg 3 times daily; Dypiridamole 75 mg + Aspirin 330 mg 3 times daily | Placebo |
| Danish Cooperative study^56^ | 1983, Denmark | TIA (203) | Mean 25 mo | No | No | ≤1 mo | 59 | 73 | Aspirin 500 mg twice daily | Placebo |
| The Canadian Cooperative Study^57^ | 1978, Canada | Ischemic stroke (585) | Mean 26 mo | Optional | No | ≤3 mo | NR | 69 | Sulfinpirazone 200 mg, Aspirin 325 mg, Sulfinpirazone 200 mg+Aspirin 325 mg 4 times daily | Placebo |

CE, cardio-embolic, h: hours; mo: months; NI: neuroimaging; NR: not reported; pts: patients;TIA, transient ischemic attack; TR: Time from first ischemic event to randomization; y: year; wk: weeks;

*All studies had a treatment duration of 1 month or longer but CAST, CARESS, COMPRESS, MAST-I, Yi 2014, THALES Study (Treatment duration <1month); De Falco (NR)

^§^total sample size <100 participants, excluded from the statistical analysis

^#^no data on the pre-defined selected outcomes, excluded from the statistical analysis

# *References*

1. Johnston SC, Amarenco P, Denison H, et al. Ticagrelor and aspirin or aspirin alone in acute ischemic stroke or TIA. *N Engl J Med* 2020;383:207-17. Doi: 10.1056/NEJMoa1916870.
2. Khazaei M, Ghasemian F, Mazdeh M, Taheri M, Ghafouri-Fard S. Comparison of administration of clopidogrel with aspirin versus aspirin alone in prevention of secondary stroke after transient ischemic attack. *Clin Transl Med*. 2019;8(1):6. doi: 10.1186/s40169-019-0223-z.
3. Kim BJ, Lee EJ, Kwon SU, et al.; PICASSO investigators. Prevention of cardiovascular events in Asian patients with ischaemic stroke at high risk of cerebral haemorrhage (PICASSO): a multicentre, randomised controlled trial. *Lancet Neurol*. 2018;17(6):509-518. doi: 10.1016/S1474-4422(18)30128-5.
4. Johnston SC, Easton JD, Farrant M, et al; Clinical Research Collaboration, Neurological Emergencies Treatment Trials Network, and the POINT Investigators. Clopidogrel and Aspirin in Acute Ischemic Stroke and High-Risk TIA. *N Engl J Med*. 2018;379(3):215-225. doi: 10.1056/NEJMoa1800410.
5. Lee JH, Hwang YH, Lee KO, et al. Effects of Triflusal and Clopidogrel on the Secondary Prevention of Stroke Based on Cytochrome P450 2C19 Genotyping. *J Stroke*. 2017;19(3):356-364. doi: 10.5853/jos.2017.01249.
6. Hong KS, Lee SH, Kim EG, et al; COMPRESS Investigators. Recurrent Ischemic Lesions After Acute Atherothrombotic Stroke: Clopidogrel Plus Aspirin Versus Aspirin Alone. *Stroke*. 2016;47(9):2323-30. doi: 10.1161/STROKEAHA.115.012293.
7. Johnston SC, Amarenco P, Albers GW, et al.; SOCRATES Steering Committee and Investigators. Ticagrelor versus Aspirin in Acute Stroke or Transient Ischemic Attack. *N Engl J Med*. 2016;375(1):35-43. doi: 10.1056/NEJMoa1603060.
8. Uchiyama S, Sakai N, Toi S, et al.; CATHARSIS Study Group. Final Results of Cilostazol-Aspirin Therapy against Recurrent Stroke with Intracranial Artery Stenosis (CATHARSIS). *Cerebrovasc Dis Extra*. 2015;5(1):1-13. doi: 10.1159/000369610.
9. Wang C, Yi X, Zhang B, Liao D, Lin J, Chi L. Clopidogrel plus aspirin prevents early neurologic deterioration and improves 6-month outcome in patients with acute large artery atherosclerosis stroke. *Clin Appl Thromb Hemost*. 2015;21(5):453-61.
10. Yi X, Lin J, Wang C, Zhang B, Chi W. A comparative study of dual versus monoantiplatelet therapy in patients with acute large-artery atherosclerosis stroke. *J Stroke Cerebrovasc Dis*. 2014;23(7):1975-81. doi: 10.1016/j.jstrokecerebrovasdis.2014.01.022.
11. Wang Y, Wang Y, Zhao X, et al.; for the CHANCE Investigators. Clopidogrel with Aspirin in Acute Minor Stroke or Transient Ischemic Attack. *N Engl J Med* 2013;369:11-19. DOI: 10.1056/NEJMoa1215340.
12. Han SW, Lee SS, Kim SH, et al. Effect of cilostazol in acute lacunar infarction based on pulsatility index of transcranial Doppler (ECLIPse): a multicenter, randomized, double-blind, placebo-controlled trial*. Eur Neurol.* 2013;69(1):33-40. doi: 10.1159/000338247.
13. Morrow DA, Alberts MJ, Mohr JP, et al.; Thrombin Receptor Antagonist in Secondary Prevention of Atherothrombotic Ischemic Events–TIMI 50 Steering Committee and Investigators. Efficacy and safety of vorapaxar in patients with prior ischemic stroke. *Stroke*. 2013;44(3):691-8. doi: 10.1161/STROKEAHA.111.000433.
14. Nakamura T, Tsuruta S, Uchiyama S. Cilostazol combined with aspirin prevents early neurological deterioration in patients with acute ischemic stroke: a pilot study. *J Neurol Sci*. 2012;313(1-2):22-6. doi: 10.1016/j.jns.2011.09.038.
15. Shinohara Y, Goto S, Doi M, Jensen P. Safety of the novel protease-activated receptor-1 antagonist vorapaxar in Japanese patients with a history of ischemic stroke. *J Stroke Cerebrovasc Dis*. 2012;21(4):318-24. doi: 10.1016/j.jstrokecerebrovasdis.2010.09.005.
16. SPS3 Investigators, Benavente OR, Hart RG, McClure LA, Szychowski JM, Coffey CS, Pearce LA. Effects of clopidogrel added to aspirin in patients with recent lacunar stroke. *N Engl J Med*. 2012;367(9):817-25. doi: 10.1056/NEJMoa1204133.
17. Lee YS, Bae HJ, Kang DW, et al. Cilostazol in Acute Ischemic Stroke Treatment (CAIST Trial): a randomized double-blind non-inferiority trial. *Cerebrovasc Dis.* 2011;32(1):65-71. doi: 10.1159/000327036.
18. Hankey GJ, Johnston SC, Easton JD, et al.; CHARISMA trial investigators. Effect of clopidogrel plus ASA vs. ASA early after TIA and ischaemic stroke: a substudy of the CHARISMA trial. *Int J Stroke*. 2011;6(1):3-9. doi: 10.1111/j.1747-4949.2010.00535.x.
19. Uchiyama S, Ikeda Y, Urano Y, Horie Y, Yamaguchi T. The Japanese aggrenox (extended-release dipyridamole plus aspirin) stroke prevention versus aspirin programme (JASAP) study: a randomized, double-blind, controlled trial. *Cerebrovasc Dis*. 2011;31(6):601-13. doi: 10.1159/000327035.
20. Bousser MG, Amarenco P, Chamorro A, et al.; PERFORM Study Investigators. Terutroban versus aspirin in patients with cerebral ischaemic events (PERFORM): a randomised, double-blind, parallel-group trial. *Lancet*. 2011;377(9782):2013-22. doi: 10.1016/S0140-6736(11)60600-4.
21. Kwon SU, Hong KS, Kang DW, et al. Efficacy and safety of combination antiplatelet therapies in patients with symptomatic intracranial atherosclerotic stenosis. *Stroke*. 2011;42(10):2883-90. doi: 10.1161/STROKEAHA.110.609370.
22. Shinohara Y, Katayama Y, Uchiyama S, et al.; for the CSPS 2 group. Cilostazol for prevention of secondary stroke (CSPS 2): an aspirin-controlled, double-blind, randomised non-inferiority trial *Lancet Neurol* 2010; 9: 959–68.
23. Guo JJ, Xu E, Lin QY, Zeng GL, Xie HF. Effect of cilostazol on cerebral arteries in secondary prevention of ischemic stroke. *Neurosci Bull*. 2009;25(6):383-90. doi: 10.1007/s12264-009-6192-2.
24. Uchiyama S, Fukuuchi Y, Yamaguchi T. The safety and efficacy of clopidogrel versus ticlopidine in Japanese stroke patients: combined results of two Phase III, multicenter, randomized clinical trials. *J Neurol*. 2009;256(6):888-97. doi: 10.1007/s00415-009-5035-4.
25. Huang Y, Cheng Y, Wu J, et al. Cilostazol versus Aspirin for Secondary Ischaemic Stroke Prevention cooperation investigators. Cilostazol as an alternative to aspirin after ischaemic stroke: a randomised, double-blind, pilot study. *Lancet Neurol*. 2008;7(6):494-9. doi: 10.1016/S1474-4422(08)70094-2.
26. Fukuuchi Y, Tohgi H, Okudera T, Ikeda Y, Miyanaga Y, Uchiyama S, Hirano M, Shinohara Y, Matsumoto M, Yamaguchi T. A randomized, double-blind study comparing the safety and efficacy of clopidogrel versus ticlopidine in Japanese patients with noncardioembolic cerebral infarction. Cerebrovasc Dis. 2008;25(1-2):40-9.
27. Sacco RL, Diener HC, Yusuf S, et al.; for the PRoFESS Study Group. Aspirin and Extended-Release Dipyridamole versus Clopidogrel for Recurrent Stroke *N Engl J Med* 2008;359:1238-51.
28. ESPRIT Study Group, Halkes PH, van Gijn J, Kappelle LJ, Koudstaal PJ, Algra A. Aspirin plus dipyridamole versus aspirin alone after cerebral ischaemia of arterial origin (ESPRIT): randomised controlled trial. *Lancet*. 2006;367(9523):1665-73. doi: 10.1016/S0140-6736(06)68734-5.
29. Markus HS, Droste DW, Kaps M, et al. Dual antiplatelet therapy with clopidogrel and aspirin in symptomatic carotid stenosis evaluated using doppler embolic signal detection: the Clopidogrel and Aspirin for Reduction of Emboli in Symptomatic Carotid Stenosis (CARESS) trial. *Circulation*. 2005;111(17):2233-40. doi: 10.1161/01.CIR.0000163561.90680.1C.
30. Chairangsarit P, Sithinamsuwan P, Niyasom S, Udommongkol C, Nidhinandana S, Suwantamee J. Comparison between aspirin combined with dipyridamole versus aspirin alone within 48 hours after ischemic stroke event for prevention of recurrent stroke and improvement of neurological function: a preliminary study. *J Med Assoc Thai.* 2005;88 Suppl 3:S148-54.
31. Diener HC, Bogousslavsky J, Brass LM, Cimminiello C, Csiba L, Kaste M, Leys D, Matias-Guiu J, Rupprecht HJ; MATCH investigators. Aspirin and clopidogrel compared with clopidogrel alone after recent ischaemic stroke or transient ischaemic attack in high-risk patients (MATCH): randomised, double-blind, placebo-controlled trial. *Lancet*. 2004 Jul 24-30;364(9431):331-7. doi: 10.1016/S0140-6736(04)16721-4.
32. Culebras A, Rotta-Escalante R, Vila J, Domínguez R, Abiusi G, Famulari A, Rey R, Bauso-Tosselli L, Gori H, Ferrari J, Reich E; TAPIRSS investigators. Triflusal vs aspirin for prevention of cerebral infarction: a randomized stroke study. *Neurology*. 2004;62(7):1073-80. doi: 10.1212/01.wnl.0000113757.34662.aa.
33. Gorelick PB, Richardson D, Kelly M, et al.; African American Antiplatelet Stroke Prevention Study Investigators. Aspirin and ticlopidine for prevention of recurrent stroke in black patients: a randomized trial. *JAMA*. 2003;289(22):2947-57. doi: 10.1001/jama.289.22.2947.
34. Matías-Guiu J, Ferro JM, Alvarez-Sabín J, et al.; TACIP Investigators. Comparison of triflusal and aspirin for prevention of vascular events in patients after cerebral infarction: the TACIP Study: a randomized, double-blind, multicenter trial. *Stroke*. 2003;34(4):840-8. doi: 10.1161/01.STR.0000063141.24491.50.
35. Ito E, Takahashi A, Yamamoto H, Kuzuhara S, Uchiyama S, Nakajima M; Tokai Panaldine Aspirin Long-Term Study (TOPALS). Ticlopidine alone versus ticlopidine plus aspirin for preventing recurrent stroke. *Intern Med*. 2003;42(9):793-9. doi: 10.2169/internalmedicine.42.793.
36. Gotoh F, Tohgi H, Hirai S, et al. Cilostazol stroke prevention study: A placebo-controlled double-blind trial for secondary prevention of cerebral infarction. *J Stroke Cerebrovasc Dis*. 2000;9(4):147-57. doi: 10.1053/jscd.2000.7216.
37. Grotemeyer KH, Evers S, Fischer M, Husstedt IW. Piracetam versus acetylsalicylic acid in secondary stroke prophylaxis. A double-blind, randomized, parallel group, 2 year follow-up study. *J Neurol Sci*. 2000;181(1-2):65-72. doi: 10.1016/s0022-510x(00)00410-x.
38. CAST: randomised placebo-controlled trial of early aspirin use in 20,000 patients with acute ischaemic stroke. CAST (Chinese Acute Stroke Trial) Collaborative Group. *Lancet*. 1997;349(9066):1641-9.
39. Bergamasco B, Benna P, Carolei A, Rasura M, Rudelli G, Fieschi C. A randomized trial comparing ticlopidine hydrochloride with indobufen for the prevention of stroke in high-risk patients (TISS Study). Ticlopidine Indobufen Stroke Study. *Funct Neurol*. 1997;12(1):33-43.
40. CAPRIE Steering Committee. A randomised, blinded, trial of clopidogrel versus aspirin in patients at risk of ischaemic events (CAPRIE). CAPRIE Steering Committee. *Lancet*. 1996;348(9038):1329-39. doi: 10.1016/s0140-6736(96)09457-3.
41. Diener HC, Cunha L, Forbes C, Sivenius J, Smets P, Lowenthal A. European Stroke Prevention Study. 2. Dipyridamole and acetylsalicylic acid in the secondary prevention of stroke. *J Neurol Sci*. 1996;143(1-2):1-13. doi: 10.1016/s0022-510x(96)00308-5.
42. Maruyama S, Uchiyama S, Tohgi H, et al. A randomized trial of E5510 versus aspirin in patients with transient ischemic attacks. The Japanese E5510 TIA study-1 (JETS-1) Group. *Angiology*. 1995;46(11):999-1008. doi: 10.1177/000331979504601104.
43. Randomised controlled trial of streptokinase, aspirin, and combination of both in treatment of acute ischaemic stroke. Multicentre Acute Stroke Trial--Italy (MAST-I) Group. *Lancet*. 1995;346(8989):1509-14.
44. Smirne S. Triflusal in the prevention of cerebrovascular attacks [Il triflusal nella prevenzione degli accidenti cerebrovasculari]. Giornale di Gerontologia 1995;43(10): 563–9. and Smirne S, Ferini-Strambi L, Cicinotta D, Zamboni M, Ambrosoli L, Poli A. Triflusal and prevention of cerebrovascular attacks: double blind clinical study vs asa. *Journal of Neurology* 1994;241(1):Abstract 53.
45. Secondary prevention in non-rheumatic atrial fibrillation after transient ischaemic attack or minor stroke. EAFT (European Atrial Fibrillation Trial) Study Group. *Lancet*. 1993;342(8882):1255-62.
46. de Falco FA, Montariello A, Mastroroberto G, Visconti OS. Efficacia clinica della picotamide [Clinical efficacy of picotamide]. *Clin Ter.* 1991;138(1):21-6.
47. Swedish Aspirin Low-Dose Trial (SALT) of 75 mg aspirin as secondary prophylaxis after cerebrovascular ischaemic events. The SALT Collaborative Group. *Lancet*. 1991;338(8779):1345-9.
48. Farrell B, Godwin J, Richards S, Warlow C. The United Kingdom transient ischaemic attack (UK-TIA) aspirin trial: final results. J Neurol Neurosurg Psychiatry. 1991;54(12):1044-54. doi: 10.1136/jnnp.54.12.1044.
49. Gent M, Blakely JA, Easton JD, et al. The Canadian American Ticlopidine Study (CATS) in thromboembolic stroke. *Lancet*. 1989;1(8649):1215-20. doi: 10.1016/s0140-6736(89)92327-1.
50. Hass WK, Easton JD, Adams HP Jr, et al. A randomized trial comparing ticlopidine hydrochloride with aspirin for the prevention of stroke in high-risk patients. Ticlopidine Aspirin Stroke Study Group. *N Engl J Med*. 1989;321(8):501-7. doi: 10.1056/NEJM198908243210804.
51. The European Stroke Prevention Study (ESPS). Principal end-points. The ESPS Group. *Lancet*. 1987;2(8572):1351-4.
52. High-dose acetylsalicylic acid after cerebral infarction. A Swedish Cooperative Study. *Stroke.* 1987;18(2):325-34. doi: 10.1161/01.str.18.2.325.
53. Persantine Aspirin Trial in cerebral ischemia. Part II: Endpoint results. The American-Canadian Co-Operative Study group. *Stroke.* 1985;16(3):406-15. doi: 10.1161/01.str.16.3.406.
54. Tohgi H, Murakami M. The effect of ticlopidine on TIA compared with aspirin: a double-blind, twelve-month follow up study. *Agents and Action Supplements* 1984;15:279-82.
55. Bousser MG, Eschwege E, Haguenau M, et al. "AICLA" controlled trial of aspirin and dipyridamole in the secondary prevention of athero-thrombotic cerebral ischemia. *Stroke*. 1983;14(1):5-14. doi: 10.1161/01.str.14.1.5.
56. Sorensen PS, Pedersen H, Marquardsen J, et al. Acetylsalicylic acid in the prevention of stroke in patients with reversible cerebral ischemic attacks. A Danish cooperative study. *Stroke*. 1983;14(1):15-22.
57. Canadian Cooperative Study Group. A randomized trial of aspirin and sulfinpyrazone in threatened stroke. *N Engl J Med*. 1978;299(2):53-9. doi: 10.1056/NEJM197807132990201.

# **Supplementary 3. Risk of bias summary table**

| **Study** | **Random sequence generation** | **Allocation concealment** | **Blinding of participants and personnel** | **Blinding of outcome assessment** | **Incomplete outcome data** | **Selective outcome reporting** | **RoB Overall** |
| --- | --- | --- | --- | --- | --- | --- | --- |
| THALES*^1^ | low risk - computer-generated | low risk - Interactive Web Response System | low risk - ticagrelor and placebo tablets identical in size, colour, smell, and taste. Each bottle labelled with a unique kit ID number. No member of the extended study delivery team at AZ, personnel at centres, or any contract research organisation handling study data will have access to the randomisation scheme | low risk - double blind | low risk - N= 11,016 (5523 ticagrelor–aspirin group and 5493 aspirin group), ITT time-to-event analysis, One patient lost to FU. Overall, 0.2% of the patients had incomplete FU for primary outcome, Data on disability missing in 2.7%. | low risk - protocol and design publication available, no discrepancies | LOW |
| Khazaei^§2^ | unclear - no information | unclear - no information | unclear risk - stated as double-blind but no further info | low risk - stated as double blind but no further information - objective outcome | low risk - N=54 apparently no drop out, no information on the type of analysis | unclear risk - no protocol | MODERATE |
| PICASSO study^3^ | low risk -computerised - blocks (block size four) stratified by centre | low risk - central randomisation service, interactive web response system | low risk - cilostazol versus aspirin (antiplatelet arm) double-blinded. double dummy—were provided every 3 months in boxes within sealed opaque envelopes | low risk - blinded trial, outcome assessors masked to treatment assignment | low risk - ITT, time to event analysis. N: 1534; ten patients did not take any study medication and 12 patients were lost to follow up. Full analysis set N=1512: 755 cilostazol and 757 aspirin completed follow up. | low risk - trial register, design publication | LOW |
| POINT trial^4^ | low risk - computerised centralised web-based system | low risk - computerised centralised web-based system | low risk - identical in size, shape, color, appearance, and taste | low risk - double blind trial and independent clinical-event committee who were unaware of group assignments adjudicated primary and secondary efficacy outcomes and major and minor bleeding events | low risk - N=4881 (clopidogrel and aspirin: 2432; placebo and aspirin: 2449); discontinuation: clopidogrel and aspirin: 29.6%; placebo and aspirin: 27.5%); withdrawal + loss to follow up: clopidogrel and aspirin: 6.4%; placebo and aspirin: 6.8% - ITT analysis | low risk - protocol available no discrepancies | LOW |
| MAESTRO Study^5^ | low risk - secure web-based registration system, telephone back up | low risk - secure web-based registration system, telephone back up | high risk -open label, few info on procedures and co-interventions | low risk - open label but all suspected outcomes were adjudicated by the Central Independent Adjudication Committee, masked to treatment allocation | low risk - N=795 (397 triflusal; 398 clopidogrel); exclusion: 6 (1.5%) triflusal; 5 (1.3%) clopidrogrel. Withdrawan: 106 (26.7%) triflusal; 81 (20.3%) clopidrogrel. Efficacy analyses performed on mITT (N=784) and the per-protocol (N=597) | low risk - design publication | HIGH |
| COMPRESS*^6^ | low risk - computer-generated randomization | low risk - central randomisation service | low risk - double blind, clopidogrel plus aspirin or placebo (matched to clopidogrel for taste, color, and size) | low risk -independent imaging review core laboratory independently assessed the MRI data blinded to the treatment allocation and clinical data. | low risk - N=358 (9 pts excluded), N=349 (174 clop+aspirin, 175 aspirin alone). 334 (95%) completed the follow up (167 for each arm) - mITT | low risk -trial registered outcome ok | LOW |
| SOCRATES Study^7^ | low risk - interactive telephone and Web-based system | low risk - interactive telephone and Web-based system | low risk - all patients will take either ticagrelor/matching placebo or ASA/matching placebo in a double-dummy fashion. Each pack will be labelled with a unique kit ID number that will be used to assign the treatment to the patient but will not indicate treatment allocation to the investigator or patient | low risk - double blind (double dummy) and independent clinical-event adjudication committee for primary and secondary efficacy end points and all bleeding events that were not reported as minimal, unaware of the treatment assignments | low risk - N=13199 (ticagrelor: 6589; aspirin: 6610); 2 lost to follow up + no info on vital status for 7 participants; discontinuation: ticagrelor: 17.5%; aspirin: 14.7%) - ITT analysis | low risk - protocol available no discrepancies | LOW |
| CATHARSIS^8^ | unclear risk - no information | unclear risk - no information | high risk - open label few info on co-interventions and adherence | unclear risk - open label but objective outcomes | low risk - N=165, cilostazol +aspirin: 83, aspirin: 82. end of follow up: cilostazol +aspirin: 77 (93%), aspirin: 75 (91%). ITT nalysis | unclear risk - no protocol or registration | HIGH |
| Wang^9^ | unclear risk - no information | low risk - randomization office of our hospital by means of sealed envelopes or allocation via the Internet | high risk - open label, few info on study procedures | low risk - open label but all reported efficacy and safety outcomes were confirmed by our central adjudication committee that was unaware of the study group assignments. | low risk - withdrawn by the local investigators: 0.7% in both groups N=570 (284 dual therapy; 286 monotherapy). Lost to follow up: 0.7% dual therapy; 0.3%monotherapy group. Discontinuation: 10 (3.5%) dual therapy; 9 (3.2%) monotherapy group. | unclear risk - no protocol or registration | HIGH |
| Yi*^10^ | low risk - no details but likely to be computerised | low risk - Randomization done through the randomization office in our hospital by means of sealed envelopes or allocation via the Internet. | unclear risk - open label but procedures apparently were well defined | low risk - open label but objective outcome | low risk - N=574; 286 clopidogrel+aspirin, 288 aspirin. 4 patients withdrawn (2 per group) | unclear risk - no protocol or registration | MODERATE |
| CHANCE trial^11^ | low risk - centralized treatment allocation system (IVRS) | low risk - centralized treatment allocation system (IVRS) | low risk - study tablets (75 mg active clopidogrel and placebo) are indistinguishable, identical in size, shape, color, appearance, and taste | low risk - blinded trial | low risk - N=5170, aspirin: 2586, clopidogrel-aspirin: 2584. A total of 197 patients were lost to follow-up at 1 year: aspirin 111 (4.3%), clopidogrel-aspirin 86 (3.3%) ITT analysis | low risk - design and protocol publication | LOW |
| ECLIPse Study^12^ | low risk - a blocked randomization procedure generated by a statistician | low risk - a blocked randomization procedure generated by a statistician was used by the central trial pharmacist to produce sequentially numbered identical study kits | low risk - blinded trial. The placebo tablets were identical in number and appearance to cilostazol tablets. Treatment assignment was masked from all investigators, study personnel, and patients throughout the trial. | low risk - blinded trial | low risk - N=203, cilostazol: 100, placebo: 103. Patients excluded before any follow-up data collection: cilostazol: 11, placebo: 10. Patients included in the intention-to-treat analysis: cilostazol: 89 (89%), placebo: 93 (90%) | low risk - trial registered | LOW |
| TIMI 50 trial^13^ | low risk - central computerized system | low risk - central computerized system through an IVRS | low risk - blinded study (matching placebo identical in apperance, label and packaging) | low risk - blinded study and all elements of the composite efficacy end points, and bleeding were adjudicated by a Clinical Events Committee blinded to treatment allocation | low risk - no details in the subgroup analysis but from the main publication: of 26,449 subjects enrolled, 32 (0.1%) lost to follow-up, and 532 (2.0%) withdrew consent for follow-up at a median of 9 months from randomization. Of the remaining 25,885 subjects who completed follow-up according to the protocol, 98.2% had ascertainment for end point events through their final study contact | low risk - registered and protocol available | LOW |
| Nakamura^§14^ | unclear risk - no information | unclear risk - no information | unclear risk - open label, pilot study procedures apparently were well defined | low risk - open label primary endpoint neurological deterioration or stroke recurrence of increased NIHSS scores; secondary endpoint neurological deterioration or stroke recurrence within 6 months. | high risk - N=76, aspirin: 38, aspirin+cilostazol: 38; day 14 primary endpoint: aspirin: 36, aspirin+cilostazol: 35; month 6 secondary endpoint: aspirin: 20 (53%), aspirin+cilostazol: 28 (74%) - on treatment analysis | unclear risk - no protocol or registration, unclear outcome definition | HIGH |
| Shinohara^§15^ | unclear risk - no information | unclear risk - no information | unclear - stated as double blind but no further information | low risk- stated as double blind but no further information objective outcome | low risk - N=90, vorapaxar (two dosages): 62; placebo 28. Completed treatment phase: vorapaxar (two dosages): 55 (89%) placebo: 27 (96%). Completed follow-up phase: vorapaxar (two dosages): 61 (100); placebo: 28 (100) | unclear risk - no protocol/registration - primary outcome safety, efficacy outcomes secondary and exploratory | MODERATE |
| SPS3 (Secondary Prevention of Small Subcortical Strokes)^16^ | low risk – computerized, stratified generated using a permuted-block design (variable block size) stored in each clinical centre's electronic data entry system | low risk -centralized, unique study identification number for the patient and systolic blood pressure target group are assigned | low risk - clopidogrel 75 mg or matching placebo (double-blind) | low risk - blinded design (clopidogrel vs placebo) + clinical stroke events (including suspected TIAs and syncopal spells of unclear cause) will be referred for blinded assessment by a board-certified neurologist | low risk - 2% were lost to follow-up, 7% withdrew consent, 5% left because of site closure, 1% withdrew at the physician’s request, and 1% withdrew for other reasons - ITT analysis | low risk - protocol available as appendix all outcome ok | LOW |
| CAIST trial^17^ | low risk - unclear reporting but "Block randomization, stratified according to the participating centers" likely to be random | low risk - unclear reporting but likely to be centralised | low risk - patients, investigators, and the sponsor personnel who monitored the study were blinded to the treatment assignments | low risk - blinded trial | low risk - ITT and PP analyses (non inferiority trial) N=458, cilostazol 231, aspirin 227; primary endpoint obtained in 447 patients. PP population 370 patients | low risk - trial registered no discrepancies | LOW |
| CHARISMA Study^18^ | low risk - likely to be computerised | low risk - Study-drug assignment was performed centrally by an interactive voice-response system | unclear risk -double blind but no further info.  Discontinuation: 20.4% clopidogrel+aspirin; 18.2 aspirin+aspirin. | low risk - All primary trial end points were adjudicated by the clinical events committee, whose members were unaware of patients’ treatment assignments | low risk - ITT/time to event analysis. Lost to follow-up (primary efficacy endpoint): 0.5% clopidogrel+aspirin, 0.4% placebo +aspirin. | low risk - design pubblication all outcome ok | MODERATE |
| JASAP Study^19^ | low risk - likely to be computerised | low risk - external enrollment center | unclear - stated as double blind but no further information | low risk - All vascular events, sudden death or bleeding events were reviewed and adjudicated by a blinded Event Assessment Committee | low risk - (full set analysis) withdrawal 32% early DP+aspirin; 28% aspirin. Lost to FU: 0% early DP+aspirin; 0.5% aspirin | low risk - trial registered | MODERATE |
| PERFORM study^20^ | low risk - the allocation sequence generated via in-house application software (balanced, non-adaptive, and stratified) | low risk - central interactive response system (telephone or internet) | low risk - patients and investigators were masked to treatment allocation, and the study treatments (terutroban and aspirin) had identical appearance. | low risk - blinded trial | low risk - N=19120 (9562; 9558) efficacy analysis 19100 patients (9556 terutroban, 9544 aspirin). 20 patients (1 centre) excluded for misconduction. 58 (<1%) patients lost to follow-up 58 and 382 (2%) withdrew consent censored at their last contact time. ITT analysis - time to event analysis. | low risk - design publication | LOW |
| TOSS-2^21^ | unclear risk - "randomised" no other details | unclear risk - "randomised" no other details | unclear risk -double blind non other info | low risk - analysis of MRI and angiographic data performed by blind investigators (outcome primary outcome: intracranial arterial stenosis via MRI; secondary outcomes: recurrent stroke and cv events open label but objective) | low risk - N=457 (randomizzati cilastazol: 232; clopidogrel: 255); MRI not performed: cilastazol: 30 (12.9%), clopidogrel: 18 (8%); full analysis datataset: cilastazol: 202; clopidogrel: 207) - 3 subjects in the cilostazol group were excluded after randomization; 1 lost to follow up (clopidrogrel) - ITT analysis for clinical events and safety | low risk - trial registered no discrepancies | MODERATE |
| CSPS-2^22^ | low risk - randomisation list done with SAS, random allocation done with a dynamic balancing method with stratification | low risk - randomisation number pre-assigned to every drug pack. Patients were assigned a treatment number matched to a numbered drug pack by the personnel responsible for drug allocation | low risk - Placebo tablets were identical in appearance. All patients, study personnel, investigators, and the sponsor were masked to treatment allocation throughout the study. | low risk - The evaluation committee, whose members were unaware of patients’ treatment assignment, adjudicated all trial endpoints | low risk- time to event analysis  discontinued drugs: 34% in the cilostazol, 25% in the aspirin group; two patients (<1%) in each group were lost to follow-up after treatment completion. | low risk - design publication and trial registration, no discrepancies | LOW |
| Guo^§23^ | low risk - computerised random number generator | unclear risk - no information | high risk - open label | unclear risk - 2 researchers who were blind to the clinical information reviewed the data; two estimators blindly reviewed the grades of arterial stenosis | unclear risk - discontinuation: 7 (20.5%) cilostazol; 6 (17.6%) aspirin group; unclear if ischemic events were assessed on the complete sample, no reasons for discontinuation - if worst case scenario (all for AE-differences are statistically significant) | unclear risk - no protocol or registration unclear reporting | HIGH |
| Uchiyama^24^ | unclear risk - no information | unclear risk - no information | low risk - double blind, double dummy (active drug and indistinguishable placebo) | low risk - blinded trial | unclear risk - N=749, 9 did not receive the study treatment, 26 were found to be ineligible, 3 failed to meet the inclusion criteria. Efficacy-evaluable population: 714 patients (clopidogrel, 366; ticlopidine, 345). Lost to follow up unclear (pooled: loss to follow-up clopidogrel: 13; ticlopidine: 14) | unclear risk - no protocol or registration | MODERATE |
| CASISP study^25^ | low risk - computer-based stratified block randomisation | unclear risk - patients were assigned to a treatment group at each centre - no other info | low risk - aspirin and cilostazol pills were made to look the same | low risk - Eight radiologists blinded to the clinical data, manually evaluated the DICOM-formatted MRIs (82%) or films (18%) independently | low risk - 1 pts excluded (asp); lost to FU: 1.6% (cil) vs 0.8% (asp); discon: 13% (cil) vs 9.7% (asp); ITT analysis | low risk - registration record, all outcomes ok | MODERATE |
| Fukuuchi^26^ | unclear risk - no information | unclear risk - no information | low risk - double blind, double dummy (active drug and indistinguishable placebo) | low risk - blinded trial | unclear risk - N=1172, 17 withdrew prior to trial initiation; 3 did not meet inclusion criteria, 1 incorreclty randomised. Full set analysis: 1151 (clopidogrel, 573; ticlopidine, 578). apparently no losts to follow-up. Discontinuation: 27% clopidogrel; 40% ticlopidine. Discontinuation due to safety endpoints 17% clopidogrel, 27% ticlopidine | unclear risk - no protocol or registration | MODERATE |
| PRoFESS^27^ | low risk - no details but likely to be computerised (more than 20,333 patients enrolled) | low risk - central telephone randomization system | low risk - double blind (double dummy, identically appearing medication kits) | low risk - double blind; adjudication and assessment committee verifies primary and secondary outcomes blinded to treatment allocation | low risk - Time to event analysis. Premature discontinuation of the study drug was significantly more frequent among patients receiving aspirin plus extended-release dipyridamole (2961 patients [29.1%]) than among those receiving clopidogrel (2290 [22.6%]) | low risk - design publication and trial registration, all outcomes ok | LOW |
| ESPRIT^28^ | low risk - computer generated randomisation codes stratified by hospital | low risk - The randomisation codes and randomisation programme were generated by a clinical epidemiologist  who was not otherwise involved in the trial; allocation by telephone call, fax, or email to the central trial office | unclear risk - open label, well-defined protocol, possible bias discussed | low risk - open label but outcome events were reported to the central trial office, clinical report of the outcome event was prepared by the trial coordinator, who removed all information about the allocated treatment. Reports were presented to three members of the auditing committee for outcome events who independently classified the event | low risk - 0.9% excluded (both groups); lost to FU: 4% (asp+dip) vs 3.5% (asp); discon: 34% (asp+dip) vs 13% (asp); ITT analysis | low risk - design publication, trial registration, all outcome ok | MODERATE |
| CARESS trial*^29^ | low risk - no details but likely to be random (patients were randomized by center in balanced blocks of size 4). | unclear - Patients were randomized by center in balanced blocks of size 4 - no other info | low risk - matching placebo (no other details) | low risk - central blinded reading of transcranial Doppler ultrasound -cerebrovascular events (TIA, ischemic stroke, and cerebral hemorrhage) | low risk - ITT (MES undetected during offline analysis: 3 dual therapy, 3 monotherapy; insufficient quality for offline analysis: 3 dual therapy, 2 monotherapy); one additional patient (dual therapy) withdrew because of myocardial infarction.) | unclear - no protocol or registration | MODERATE |
| Chairangsarit^§30^ | unclear risk - no information | unclear risk - Treatment group allocation was determined by an open label, blind assessed randomization system | high risk open label - few info on procedures and co-interventions | unclear risk - open label, no info on outcome assessment | high risk - N=38 (18 aspirin; 20 aspirin+dipyridamole) - After 6 months follow up, available data was 28 patients (73.7%): 1 patient (2.6%) who developed a severe adverse event that required cessation of treatment and 7 (18.4%) lost to follow up (6 in the aspirin group, 3 aspirin+dipyridamole). | unclear - no protocol or registration, pilot project | HIGH |
| MATCH^31^ | low risk - computer-generated list | low risk - central treatment allocation, with an interactive voiceresponse system (by phone) | low risk - double blind | low risk - double blind | low risk - follow-up data available for 96%; ITT analysis | low risk - design publication all outcome ok | LOW |
| TAPIRSS Study^32^ | low risk - randomization schedule was computer generated | low risk - randomization schedule kept unknown to all study-related personnel until closure of the database. | low risk - both treatment forms were indistinguishable in color, shape, form, and packaging Boxes were identified by a unique number | low risk - both treatment forms were indistinguishable in color, shape, form, and packaging | low risk - N=431 (216 aspirin; 215 triflusal); 1 patient excluded by the ITT analysis; discontinuation rate 33.8% (35.6% aspirin group; 31.9% triflusal group); noncompliance 63 (31.8%) aspirin group; 62 (32.0%) triflusal group - ITT analysis | low risk - no protocol, no registration but outcomes seem ok | LOW |
| AAASPS (Aspirin and Ticlopidine for prevention of recurrent stroke in black pts)^33^ | low risk - randomization algorithm developed by the Chief Study Statistician at the Data monitoring committee | low risk - centralised (phone Automated Phone Registration System). | low risk - all personnel masked to study treatment; Ticlopidine and aspirin are dispensed in tablet form with "dummy" tablets (placebo) of identical physical properties from plastic bottles | low risk - blinded design | low risk - N= 1809 (902 ticlopine, 907 aspirin) - 41% ticlopidine, 44.4% aspirin completed the 24-month - trial interrupted for futility. All patients analysed | low risk - design publication all outcome ok | LOW |
| TACIP study^34^ | low risk - likely to be computerised | low risk - random list prepared by an independent statistical center; treatments allocated to the random codes by personnel unrelated to the study | low risk - double blind (uncoated triflusal or aspirin) | low risk - blinded study and data-blind review committee appointed by the executive committee responsible for validating all reported primary and secondary end points | low risk - N=2113 (triflusal 1058, aspirin 1055), 6 subjects excluded from ITT (2107, 1055 triflusal and 1052 aspirin); discontinuation: 306 (29%) triflusal 284 (26.9%), reasons similar - ITT and PP analyses | low risk - no protocol available but outcomes ok | LOW |
| TOPALS study^35^ | unclear risk - "randomised" no other details | unclear risk - "randomised" no other details | high risk - open label, few info on procedures and co-interventions | unclear risk - open label, unclear outcome definition | low risk - 276 randomised, 270 analysed; excludedi 6 (2.2%) - no info on analysis | unclear risk - no protocol or registration unclear reporting | HIGH |
| Gotoh^36^ | low risk - dynamic balancing method (likely to be computerised) | low risk - randomisation done by the Registration and Analysis Center, an independent organization | low risk - medications supplied in unidentifiable tablets and containers | low risk - patients, investigators, and the study’s sponsor remained blinded to assignments of the treatment throughout the study. Evaluation Committee (blinded members) | low risk N=1095 (544 cilostazol; 548 placebo); Excluded: 11 cilostazol; 14 placebo; primary analysis: evaluable patient population who were eligible and followed-up until termination of the protocol treatment without major violation: 526 (96.7%); 526 (96%) - ITT for sensitivity analyses. | low risk - design publication | LOW |
| Grotemeyer^37^ | unclear risk - "randomised" no other details | unclear risk - "randomised" no other details | unclear - stated as double blind but no further information | low risk - stated as double blind but no further information objective outcome | low risk - N=563 (256 piracetam; 307 aspirin); N=41; 7.4 and 7.2% in the piracetam and the ASA group; in the per protocol analyisis: 52% piracetam; 46% aspirin - per protocol primary analysis | low risk - no protocol, no registration but outcomes seem ok | MODERATE |
| CAST* (Chinese Acute Stroke Trial)^38^ | unclear risk - no information | low risk - prepacked, sequentially numbered trial envelopes, produced centrally; random allocation unknown to the participating physicians | unclear risk - open label pragmatic trial. All other decisions about management of patients were at the discretion of the responsible doctor, except that non-trial aspirin or other antiplatelet drugs were not allowed during the trial period | low risk - objective outcome | low risk - discharge forms available for 20 655 (97.9%) of the randomised patients; balanced in the two groups | low risk - no protocol but all outcomes ok | MODERATE |
| TISS (Ticlopidine Indobufen Stroke Study)^39^ | unclear risk - no information | low risk - centralised, balanced by centre | high risk open label - few info on procedures and co-intervention | low risk- blinded assessment of outcome by Independednt committee | low risk - N=1632 (821 ticlopidine; 811 indobufen), discontinuation: ticlopidine 111 (13.5% ); indobufen 98 (12.1); all discontinued patienst were observed up to the end of the trial. ITT analysis | low risk - no protocol, no registration but outcome ok | HIGH |
| CAPRIE^40^ | low risk - computer-generated balanced blocks | Low risk -  access to random code restricted to the Independent Statistical Centre, the Chairman of the External Safety and Efficacy Monitoring Committee, and companies responsible for study drugs | low risk - Patients were allocated study drugs sequentially from supplies at the clinical centre packaged in a predetermined order; blister packs indistinguishable from one another. | low risk - blinded trial, objective outcomes | low risk - 0.22% lost to follow up; discontinuation 21.3% (clopidogrel 21.1% (aspirin) ITT | low risk - no protocol but all outcomes ok | LOW |
| European Stroke Prevention Study 2^41^ | low risk - computerized randomization system (minimization) | low risk - central allocation (computer based) | low risk - blinded study no details, no discrepancies | low risk -patients, investigators, steering commitee, laboratory analyses blinded to study treatment allocation | low risk - 0.64% lost to follow up; ITT analysis. 438 patients excluded before unblinding due to serious inconsistencies in one centre | low risk no protocol but all outcomes ok | LOW |
| JETS-1^42^ | unclear risk - no information | low risk - drugs allocated by a third party (controller committee), which held the randomization codes at the institution until code break | low risk - drugs administered included E5510 (1 and 2 mg tablets), aspirin (81 mg tablets), and placebo tablets that were indistinguishable from the active drugs. One E5510 tablet and 2 aspirin placebo tablets or 1 E5510 placebo tablet and 2 aspirin tablets were administered | low risk -double blind | low risk - N= 227 (aspirin: 79; E5510 high dose: 71; E5510 low dose: 77); 3 excluded from safety population (N=224), 1 lost to follow up; 37 excluded from treatment analysis (N=187; aspirin: 66 (83.5%); E5510 high dose: 55 (77.5%); E5510 low dose: 66 (85.7%)) - ITT and treatment analysis | unclear risk - no protocol, no registration unclear reporting | LOW |
| MAST-I*^43^ | low risk - computerised | low risk - centralised 24-hour telephone service | unclear risk - open label pragmatic trial but procedures and co-intervention were well-defined in the protocol. | low risk - evaluation performed by a single rater "blind" to treatment allocation. CT scan and clinical record of any patient who died or had a major in hospital event was blindly reviewed by the clinical reviewing committee | low risk - N=622, 4 groups: 157 streptokinase, 153 aspirin, 156 streptokinase plus aspirin, 156 neither. None of the 622 patients was lost to follow-up. | low risk - design and protocol publication | MODERATE |
| Smirne^#44^ | low risk -randomisation list | unclear risk - | unclear risk - double-blind, double-dummy no other info | low risk - blinded trial | low risk N=183 There were 9 withdrawals related to: side effects (3), adverse events (2), treatment refusal (2), lost to follow up (1), and other (1). | unclear risk - no protocol | MODERATE |
| EAFT Study^45^ | low risk - computerised, pre-generated randomisation lists | low risk - treatment codes distributed centrally by phone through a randomisation service; only the trial pharmacist had access to the treatment codes | low risk - aspirin and placebo are used in a double-blinded manner | low risk - blinded study. All outcome events were independently classified by at least 3 members of the Auditing Committee for Outcome Events, unaware of the allocated treatment | low risk - 5 patients inappropriately enrolled in the study (included in the ITT) - lost to follow up not clear but apparently none | low risk - no protocol but design publication all outcomes ok | LOW |
| De Falco*^§46^ | unclear risk - "randomised" no other details | unclear risk - "randomised" no other details | high risk - open label, few info on procedures and co-intervention | unclear risk - open label, few info on outcome evaluation | unclear risk - no information on lost to follow up | high risk - no protocol, no registration, incomplete reporting of data | HIGH |
| SALT Study^47^ | low risk - no information, but stratification and use of block | unclear risk - prescribed randomisation arrangement generated separately for each clinical centre; Each patient received a randomisation code number, according to which the local pharmacy supplied the study drug. A sealed envelope, with information on the treatment allocated, was kept in the clinical file of each patient. | low risk - placebo tablets were .identical in appearance and packaging | low risk - all reported outcome events were reviewed by the relevant adjudication committee, unaware of treatment given or results of platelet function tests. | low risk - ITT analysis. N=1360 (676 aspirin, 684 placebo); 21 ineligible but included in all analyses; 5 patients (3 from the aspirin group) lost to follow-up. Permanent withdrawn before the end of the study (excluding discontinuations due to primary outcome events): 115 (17%) aspirin; 157 (23%) placebo | low risk no protocol but all outcomes ok | MODERATE |
| UK-TIA Study^48^ | low risk -no information but likely to be random (Oxford trial unit) | low risk - telephone | low risk - blinded trial | low risk - blinded trial | low risk - ITT analysis 12 (0.5%) withdrawn (intracranial tumours); 1 patient lost to follow up - At each follow up about 85% of patients reported that they were taking the trial medication | unclear risk - no protocol, change to "fundamental analysis" | LOW |
| CATS (Canadian American Ticlopidine study)^49^ | unclear risk - no details on sequence generation (randomization arrangement generated separately for each clinical center) | low risk - treatment code was known only to the Interim Analysis Group and the Safety Committee | low risk - placebo and ticlopidine tablets identical in appearance and packaging; only the Safety Committee knew the randomization code for any particular clinical center | low risk - reviewing physicians unaware of the study medication given to individual patients | low risk - 19/1072 (1.8%) excluded as ineligible (13 placebo; 6 ticlopidine); 4 patients lost to follow up (3 ticlopidine); discontinuation 40% placebo 52% ticlopidine; primary analysis excluded events occurring more than 28 days after permanent discontinuation of study drug. ITT was also performed: data are consistent with primary analysis magnitide of benefit smaller. | low risk- design publication all outcomes ok | LOW |
| Ticlopidine Aspirin Stroke Study50 | low risk - no details but stratified | low risk - centralised , independent | low risk - drugs supplied in identical capsules and containers, patients investigators, sponsore remained blinded | low risk - blinded design, blinded outcome adjudicators | low risk - 0.4% excluded (3 ticl, 9 asp); lost to FU: 3% (ticl) vs 2% (asp); discon: 52% (ticl) vs 47% (asp); ITT analysis | low risk - design publication, all outcomes ok | LOW |
| ESPS Study^51^ | unclear risk - no information | low risk - no information on allocation concealment but multicentric blinded trial | low risk - blinded matching placebo | low risk - blinded | low risk - Lost to follow up 7/2500. ITT: 639/2500 not eligible after review by blinded coordinating committee are included in the ITT. 1298 completed the follow up; 800 patients dropped out (426 DP-ASA, 34%, and 374 placebo, 30%) | low risk no protocol but all outcome ok | LOW |
| Swedish Cooperative Study^52^ | unclear risk - no information | low risk - patient assigned to a consecutive number corresponding to a numbered bottle; blinded study; regimen seperately randomized for each centre | low risk - bottles identical for active drug and placebo as well as size, shape, weight and color of the tablets | low risk - bottles identical for active drug and placebo as well as size, shape, weight and color of the tablets | low risk - no lost at FU - 24% in ASA 21% in placebo were withdrawn due to poor compliance or concomitant medication but data analysed by ITT | low risk - no protocol but all outcome ok | LOW |
| Persantine Aspirin Trial^53^ | low risk -no details, but central allocation considered adequate | low risk- central allocation | low risk - blinding described and assessed | low risk - blinded trial | low risk - about 4% in each group lost to FU | low risk - no protocol but all outcomes ok | LOW |
| Tohgi^54^ | unclear - no information | low risk - two control officier not involved with the clinical institutions held the treatment code during the study period | unclear risk -double blind non other info | low risk - double blind non other info but objective outcome | unclear risk - N=340, analysed ticlopidine: 165, aspirin: 169. But "at the end of the trial, but before the code was broken, the central committee considered the eligibility of patients for analysis. Excluded from efficacy analysis: questionable diagnosis of TIA: 5 ticlopidin, 1 aspirin; TIA not compatible with outline of cerebrovascular disease 21 ticlopidin, 18 aspirin; medication discontinued: 8 ticlopidine, 6 aspirin." | unclear risk - no protocol or registration | MODERATE |
| AICLA study^55^ | low risk - previously established randomization schedule (list of randomisation) | low risk - previously established randomization schedule in a blinded trial | low risk - active drugs and placebo were identical in size, shape, weight and color | low risk - active drugs and placebo were identical in size, shape, weight and color | low risk - Sixty-six patients (11%) withdrawn from the trial for reasons not strictly related to health problems: 12 moved away, 8 were lost to follow-up and 46 gave poor cooperation. No differences among treatment groups | low risk -protocol in French, primary outcome ok | LOW |
| Danish Cooperative study^56^ | low risk - computerised | unclear risk - no details + "every team consecutive patients in a partecipating department, five were allocated to aspirin and five to placebo" | low risk - ASA and placebo in identically appearing tablets | low risk - blinded trial | low risk - no lost to follow up | low risk no protocol but all outcome ok | MODERATE |
| The Canadian Cooperative Study^57^ | low risk - no details, but central allocation and stratification considered adequate for that period | low risk - central allocation and stratification | low risk - blinding described and assessed | low risk - blinded trial | low risk - 10% patients excluded for protocol violation, some imbalance but then 99% FU measure ok | low risk - no protocol but all outcome ok | LOW |

# *References*

See Supplementary 2

# **Supplementary 4. Table of ongoing studies (last update December 2020)**

| **Study title** | **Intervention** | **Comparator** | **Estimated sample size** | **Primary outcome** | **Registration number** |
| --- | --- | --- | --- | --- | --- |
| Tirofiban for the Prevention of Neurological Deterioration in Acute Ischemic Stroke | Tirofiban in the first 72 hours | Aspirin, clopidogrel or other antiplatelet drugs | 420 | Number of patients with a change in NIHSS by ≥ 4 points compared to enrollment NIHSS | NCT04491695 |
| Ticagrelol Versus Aspirin in Ischemic Stroke | Ticagrelor 180 mg | Aspirin 300 mg | 152 | Hemorrhagic transformation of infarction and peripheral bleeding | NCT03884530 |
| LACunar Intervention (LACI-2) Trial-2: Assessment of Safety and Efficacy of Cilostazol and Isosorbide Mononitrate to Prevent Recurrent Lacunar Stroke and Progression of Cerebral Small Vessel Disease | -Cilostazol  -Isosorbide Mononitrate  -Cilostazol + Isosorbide Mononitrate | Neither Isosorbide Mononitrate nor cilostazol | 400 | Feasibility of Phase III trial | NCT03451591 |
| Antiplatelet Therapy in Acute Mild-Moderate Ischemic Stroke (ATAMIS) | Clopidogrel + aspirin | Aspirin | 3000 | Early neurological deterioration assessed as change of NIHSS | NCT02869009 |

# **Supplementary 5. Network plots of evidence for secondary outcomes**

Each line links the treatments that have been directly compared in studies. The thickness of the line is proportional to the precision of each direct estimate, and the width of each circle is proportional to the number of studies included in the treatment. Abbreviations: Notrt, no treatment.

## *Ischemic stroke*

## *Ischemic stroke or transient ischemic attack*

## *Cardiovascular event*

## *Hemorrhagic stroke*

## *Intracranial haemorrhage*

## *Major bleeding*

# **Supplementary 6. Results of the pairwise meta-analyses** **for primary and secondary outcomes**

## *All strokes*

Legend: 01=Placebo / No treatment; 02=Aspirin>150 mg; 03=Aspirin≤150 mg; 04=Clopidogrel 75 mg + Aspirin; 05=Dipyridamole + Aspirin; 06=Cilostazol 200 mg; 07=Cilostazol 200 mg + Aspirin; 08=Clopidogrel 75 mg; 09=Dipyridamole>200 mg; 10=Indobufen 200 mg; 11=Piracetam 4800 mg; 12=Sulfinpyrazone 800 mg + Aspirin>150 mg; 13=Sulfinpyrazone 800 mg; 14=Terutroban 30 mg; 15=Ticagrelor 180 mg; 16=Ticlopidine≤500 mg; 17=Ticlopidine<500 mg + Aspirin≤150 mg; 18=Triflusal 600 mg; 19=Vorapaxar 2.5 mg; 20=Ticagrelor 180 mg + Aspirin≤150 mg.

Study | Odds Ratio [95% Conf. Interval]

---------------------+---------------------------------------------------

02 - 01

CANADIAN CS 1978 | 1.073 0.557 2.068

SWEDISH CS 1987 | 0.995 0.589 1.682

EAFT 1993 | 0.891 0.638 1.245

CAST 1997 | 0.951 0.817 1.108

AICLA 1983 | 0.550 0.301 1.004

DANISH CS 1983 | 1.674 0.742 3.780

UK-TIA 1991 | 0.827 0.648 1.055

Sub-total |

D+L pooled ES | 0.913 0.813 1.026

---------------------+---------------------------------------------------

12 - 01

CANADIAN CS 1978 | 0.631 0.305 1.305

Sub-total |

D+L pooled ES | 0.631 0.305 1.305

---------------------+---------------------------------------------------

13 - 01

CANADIAN CS 1978 | 1.359 0.729 2.531

Sub-total |

D+L pooled ES | 1.359 0.729 2.531

---------------------+---------------------------------------------------

12 - 02

CANADIAN CS 1978 | 0.588 0.288 1.201

Sub-total |

D+L pooled ES | 0.588 0.288 1.201

---------------------+---------------------------------------------------

13 - 02

CANADIAN CS 1978 | 1.266 0.690 2.324

Sub-total |

D+L pooled ES | 1.266 0.690 2.324

---------------------+---------------------------------------------------

13 - 12

CANADIAN CS 1978 | 2.153 1.088 4.262

Sub-total |

D+L pooled ES | 2.153 1.088 4.262

---------------------+---------------------------------------------------

05 - 02

PERSANTIN ASPIRIN TR | 0.854 0.575 1.268

AICLA 1983 | 0.978 0.501 1.909

Sub-total |

D+L pooled ES | 0.885 0.630 1.243

---------------------+---------------------------------------------------

05 - 01

ESPS 1987 | 0.581 0.454 0.745

AICLA 1983 | 0.538 0.295 0.982

ESPS2 1996 | 0.588 0.476 0.728

Sub-total |

D+L pooled ES | 0.582 0.498 0.680

---------------------+---------------------------------------------------

03 - 01

SALT 1991 | 0.815 0.605 1.098

ESPS2 1996 | 0.799 0.655 0.974

Sub-total |

D+L pooled ES | 0.804 0.681 0.948

---------------------+---------------------------------------------------

04 - 03

COMPRESS 2016 | 0.593 0.139 2.521

POINT 2018 | 0.736 0.575 0.943

CHARISMA 2011 | 0.794 0.610 1.033

CHANCE 2013 | 0.673 0.560 0.810

Sub-total |

D+L pooled ES | 0.717 0.630 0.815

---------------------+---------------------------------------------------

04 - 02

SPS3 2012 | 0.888 0.689 1.144

WANG 2015 | 0.303 0.119 0.771

Sub-total |

D+L pooled ES | 0.572 0.203 1.612

---------------------+---------------------------------------------------

16 - 10

TISS 1997 | 0.570 0.311 1.046

Sub-total |

D+L pooled ES | 0.570 0.311 1.046

---------------------+---------------------------------------------------

14 - 03

PERFORM 2011 | 1.017 0.920 1.125

Sub-total |

D+L pooled ES | 1.017 0.920 1.125

---------------------+---------------------------------------------------

08 - 04

MATCH 2004 | 1.024 0.876 1.199

Sub-total |

D+L pooled ES | 1.024 0.876 1.199

---------------------+---------------------------------------------------

09 - 01

ESPS2 1996 | 0.818 0.672 0.997

Sub-total |

D+L pooled ES | 0.818 0.672 0.997

---------------------+---------------------------------------------------

05 - 03

ESPS2 1996 | 0.737 0.591 0.918

JASAP 2011 | 1.474 0.966 2.249

Sub-total |

D+L pooled ES | 1.017 0.516 2.003

---------------------+---------------------------------------------------

09 - 03

ESPS2 1996 | 1.024 0.834 1.258

Sub-total |

D+L pooled ES | 1.024 0.834 1.258

---------------------+---------------------------------------------------

09 - 05

ESPS2 1996 | 1.391 1.117 1.731

Sub-total |

D+L pooled ES | 1.391 1.117 1.731

---------------------+---------------------------------------------------

16 - 01

CATS 1989 | 0.561 0.390 0.809

Sub-total |

D+L pooled ES | 0.561 0.390 0.809

---------------------+---------------------------------------------------

16 - 02

AAASPS 2003 | 1.274 0.942 1.723

TASS 1989 | 0.794 0.640 0.984

Sub-total |

D+L pooled ES | 0.993 0.625 1.578

---------------------+---------------------------------------------------

11 - 02

GROTEMEYER 2000 | 1.396 0.776 2.511

Sub-total |

D+L pooled ES | 1.396 0.776 2.511

---------------------+---------------------------------------------------

18 - 08

MAESTRO 2017 | 1.328 0.729 2.422

Sub-total |

D+L pooled ES | 1.328 0.729 2.422

---------------------+---------------------------------------------------

06 - 03

CASISP 2008 | 0.584 0.281 1.214

CSPS2 2010 | 0.668 0.499 0.894

PICASSO 2018 | 0.636 0.435 0.929

Sub-total |

D+L pooled ES | 0.649 0.520 0.809

---------------------+---------------------------------------------------

17 - 16

TOPALS 2003 | 1.369 0.495 3.790

Sub-total |

D+L pooled ES | 1.369 0.495 3.790

---------------------+---------------------------------------------------

15 - 03

SOCRATES 2016 | 0.861 0.749 0.991

Sub-total |

D+L pooled ES | 0.861 0.749 0.991

---------------------+---------------------------------------------------

07 - 04

TOSS-2 2011 | 1.817 0.660 4.999

Sub-total |

D+L pooled ES | 1.817 0.660 4.999

---------------------+---------------------------------------------------

19 - 01

TIMI-50 2013 | 1.129 0.898 1.419

Sub-total |

D+L pooled ES | 1.129 0.898 1.419

---------------------+---------------------------------------------------

08 - 05

PRoFESS 2008 | 0.982 0.891 1.081

Sub-total |

D+L pooled ES | 0.982 0.891 1.081

---------------------+---------------------------------------------------

07 - 03

CATHARSIS 2015 | 0.456 0.132 1.578

ECLIPSE 2013 | 1.030 0.064 16.700

Sub-total |

D+L pooled ES | 0.522 0.168 1.622

---------------------+---------------------------------------------------

20 - 03

THALES 2019 | 0.804 0.684 0.945

Sub-total |

D+L pooled ES | 0.804 0.684 0.945

---------------------+---------------------------------------------------

Test(s) of heterogeneity:

Heterogeneity degrees of

statistic freedom P I-squared** Tau-squared

02 - 01 6.13 6 0.409 2.0% 0.0006

12 - 01 0.00 0 . .% 0.0000

13 - 01 0.00 0 . .% 0.0000

12 - 02 0.00 0 . .% 0.0000

13 - 02 0.00 0 . .% 0.0000

13 - 12 0.00 0 . .% 0.0000

05 - 02 0.12 1 0.733 0.0% 0.0000

05 - 01 0.08 2 0.963 0.0% 0.0000

03 - 01 0.01 1 0.915 0.0% 0.0000

04 - 03 1.12 3 0.772 0.0% 0.0000

04 - 02 4.74 1 0.029 78.9% 0.4556

16 - 10 0.00 0 . .% 0.0000

14 - 03 0.00 0 . .% 0.0000

08 - 04 0.00 0 . .% 0.0000

09 - 01 0.00 0 . .% 0.0000

05 - 03 8.14 1 0.004 87.7% 0.2109

09 - 03 0.00 0 . .% 0.0000

09 - 05 0.00 0 . .% 0.0000

16 - 01 0.00 0 . .% 0.0000

16 - 02 6.25 1 0.012 84.0% 0.0939

11 - 02 0.00 0 . .% 0.0000

18 - 08 0.00 0 . .% 0.0000

06 - 03 0.13 2 0.939 0.0% 0.0000

17 - 16 0.00 0 . .% 0.0000

15 - 03 0.00 0 . .% 0.0000

07 - 04 0.00 0 . .% 0.0000

19 - 01 0.00 0 . .% 0.0000

08 - 05 0.00 0 . .% 0.0000

07 - 03 0.27 1 0.600 0.0% 0.0000

20 - 03 0.00 0 . .% 0.0000

** I-squared: the variation in ES attributable to heterogeneity

Significance test(s) of ES=1

02 - 01 z= 1.53 p = 0.125

12 - 01 z= 1.24 p = 0.214

13 - 01 z= 0.97 p = 0.334

12 - 02 z= 1.46 p = 0.145

13 - 02 z= 0.76 p = 0.446

13 - 12 z= 2.20 p = 0.028

05 - 02 z= 0.71 p = 0.481

05 - 01 z= 6.80 p = 0.000

03 - 01 z= 2.59 p = 0.010

04 - 03 z= 5.08 p = 0.000

04 - 02 z= 1.06 p = 0.291

16 - 10 z= 1.81 p = 0.070

14 - 03 z= 0.33 p = 0.740

08 - 04 z= 0.30 p = 0.763

09 - 01 z= 1.99 p = 0.046

05 - 03 z= 0.05 p = 0.962

09 - 03 z= 0.23 p = 0.819

09 - 05 z= 2.95 p = 0.003

16 - 01 z= 3.10 p = 0.002

16 - 02 z= 0.03 p = 0.977

11 - 02 z= 1.11 p = 0.266

18 - 08 z= 0.93 p = 0.354

06 - 03 z= 3.84 p = 0.000

17 - 16 z= 0.61 p = 0.545

15 - 03 z= 2.09 p = 0.037

07 - 04 z= 1.16 p = 0.248

19 - 01 z= 1.04 p = 0.299

08 - 05 z= 0.38 p = 0.706

07 - 03 z= 1.12 p = 0.261

20 - 03 z= 2.65 p = 0.008

## *All-cause mortality*

Legend: 01=Placebo / No treatment; 02=Aspirin>150 mg; 03=Aspirin≤150 mg; 04=Clopidogrel 75 mg + Aspirin; 05=Dipyridamole + Aspirin; 06=Cilostazol 200 mg; 07=Cilostazol 200 mg + Aspirin; 08=Clopidogrel 75 mg; 09=Dipyridamole>200 mg; 10=Indobufen 200 mg; 11=Piracetam 4800 mg; 12=Sulfinpyrazone 800 mg + Aspirin>150 mg; 13=Sulfinpyrazone 800 mg; 14=Terutroban 30 mg; 15=Ticagrelor 180 mg; 16=Ticlopidine≤500 mg; 17=Triflusal 600 mg; 18=Vorapaxar 2.5 mg; 19=Ticagrelor 180 mg + Aspirin≤150 mg.

Study | Odds Ratio [95% Conf. Interval]

---------------------+---------------------------------------------------

02 - 01

CANADIAN CS 1978 | 0.646 0.267 1.564

SWEDISH CS 1987 | 0.995 0.593 1.670

EAFT 1993 | 0.952 0.691 1.312

CAST 1997 | 0.858 0.746 0.988

AICLA 1983 | 1.497 0.558 4.014

DANISH CS 1983 | 1.011 0.341 2.993

UK-TIA 1991 | 0.895 0.705 1.137

MAST-I 1995 | 0.794 0.395 1.597

Sub-total |

D+L pooled ES | 0.883 0.793 0.983

---------------------+---------------------------------------------------

12 - 01

CANADIAN CS 1978 | 0.562 0.225 1.400

Sub-total |

D+L pooled ES | 0.562 0.225 1.400

---------------------+---------------------------------------------------

13 - 01

CANADIAN CS 1978 | 0.808 0.356 1.834

Sub-total |

D+L pooled ES | 0.808 0.356 1.834

---------------------+---------------------------------------------------

12 - 02

CANADIAN CS 1978 | 0.870 0.326 2.320

Sub-total |

D+L pooled ES | 0.870 0.326 2.320

---------------------+---------------------------------------------------

13 - 02

CANADIAN CS 1978 | 1.250 0.510 3.061

Sub-total |

D+L pooled ES | 1.250 0.510 3.061

---------------------+---------------------------------------------------

13 - 12

CANADIAN CS 1978 | 1.438 0.570 3.624

Sub-total |

D+L pooled ES | 1.438 0.570 3.624

---------------------+---------------------------------------------------

05 - 02

PERSANTIN ASPIRIN TR | 1.217 0.775 1.910

AICLA 1983 | 0.775 0.299 2.007

Sub-total |

D+L pooled ES | 1.120 0.745 1.684

---------------------+---------------------------------------------------

05 - 01

ESPS 1987 | 0.663 0.512 0.859

AICLA 1983 | 1.161 0.413 3.262

ESPS2 1996 | 0.905 0.732 1.119

Sub-total |

D+L pooled ES | 0.802 0.616 1.045

---------------------+---------------------------------------------------

03 - 01

SALT 1991 | 0.884 0.615 1.270

ESPS2 1996 | 0.889 0.718 1.100

Sub-total |

D+L pooled ES | 0.888 0.739 1.066

---------------------+---------------------------------------------------

04 - 03

COMPRESS 2016 | 7.128 0.365 139.069

POINT 2018 | 1.514 0.728 3.150

CARESS 2005 | 1.097 0.021 56.302

CHARISMA 2011 | 0.774 0.543 1.103

CHANCE 2013 | 1.001 0.416 2.408

Sub-total |

D+L pooled ES | 0.968 0.667 1.405

---------------------+---------------------------------------------------

04 - 02

SPS3 2012 | 1.491 1.105 2.010

WANG 2015 | 1.007 0.141 7.199

YI 2014 | 1.007 0.141 7.199

Sub-total |

D+L pooled ES | 1.465 1.094 1.962

---------------------+---------------------------------------------------

16 - 10

TISS 1997 | 0.448 0.224 0.894

Sub-total |

D+L pooled ES | 0.448 0.224 0.894

---------------------+---------------------------------------------------

14 - 03

PERFORM 2011 | 1.011 0.899 1.138

Sub-total |

D+L pooled ES | 1.011 0.899 1.138

---------------------+---------------------------------------------------

17 - 02

TAPIRSS 2004 | 0.453 0.169 1.214

TACIP 2003 | 1.203 0.837 1.729

Sub-total |

D+L pooled ES | 0.825 0.325 2.096

---------------------+---------------------------------------------------

08 - 04

MATCH 2004 | 0.999 0.817 1.221

Sub-total |

D+L pooled ES | 0.999 0.817 1.221

---------------------+---------------------------------------------------

09 - 01

ESPS2 1996 | 0.919 0.744 1.135

Sub-total |

D+L pooled ES | 0.919 0.744 1.135

---------------------+---------------------------------------------------

05 - 03

ESPS2 1996 | 1.018 0.819 1.265

ESPRIT 2006 | 0.868 0.651 1.159

JASAP 2011 | 0.388 0.121 1.244

Sub-total |

D+L pooled ES | 0.916 0.717 1.169

---------------------+---------------------------------------------------

09 - 03

ESPS2 1996 | 1.034 0.833 1.283

Sub-total |

D+L pooled ES | 1.034 0.833 1.283

---------------------+---------------------------------------------------

09 - 05

ESPS2 1996 | 1.016 0.819 1.260

Sub-total |

D+L pooled ES | 1.016 0.819 1.260

---------------------+---------------------------------------------------

16 - 01

CATS 1989 | 1.007 0.695 1.457

Sub-total |

D+L pooled ES | 1.007 0.695 1.457

---------------------+---------------------------------------------------

16 - 02

AAASPS 2003 | 1.138 0.736 1.761

TASS 1989 | 0.886 0.713 1.101

Sub-total |

D+L pooled ES | 0.932 0.766 1.135

---------------------+---------------------------------------------------

06 - 01

GOTOH 2000 | 0.900 0.363 2.233

Sub-total |

D+L pooled ES | 0.900 0.363 2.233

---------------------+---------------------------------------------------

11 - 02

GROTEMEYER 2000 | 1.362 0.518 3.582

Sub-total |

D+L pooled ES | 1.362 0.518 3.582

---------------------+---------------------------------------------------

06 - 03

CASISP 2008 | 0.595 0.141 2.508

CSPS2 2010 | 0.998 0.461 2.162

PICASSO 2018 | 0.902 0.536 1.519

Sub-total |

D+L pooled ES | 0.897 0.593 1.357

---------------------+---------------------------------------------------

15 - 03

SOCRATES 2016 | 1.178 0.828 1.675

Sub-total |

D+L pooled ES | 1.178 0.828 1.675

---------------------+---------------------------------------------------

07 - 04

TOSS-2 2011 | 0.483 0.043 5.361

Sub-total |

D+L pooled ES | 0.483 0.043 5.361

---------------------+---------------------------------------------------

18 - 01

TIMI-50 2013 | 1.186 0.877 1.604

Sub-total |

D+L pooled ES | 1.186 0.877 1.604

---------------------+---------------------------------------------------

08 - 05

PRoFESS 2008 | 1.028 0.925 1.142

Sub-total |

D+L pooled ES | 1.028 0.925 1.142

---------------------+---------------------------------------------------

07 - 03

CATHARSIS 2015 | 0.964 0.019 49.170

ECLIPSE 2013 | 1.030 0.020 52.402

Sub-total |

D+L pooled ES | 0.996 0.062 16.052

---------------------+---------------------------------------------------

19 - 03

THALES 2019 | 1.328 0.805 2.191

Sub-total |

D+L pooled ES | 1.328 0.805 2.191

---------------------+---------------------------------------------------

Test(s) of heterogeneity:

Heterogeneity degrees of

statistic freedom P I-squared** Tau-squared

02 - 01 2.31 7 0.940 0.0% 0.0000

12 - 01 0.00 0 . .% 0.0000

13 - 01 0.00 0 . .% 0.0000

12 - 02 0.00 0 . .% 0.0000

13 - 02 0.00 0 . .% 0.0000

13 - 12 0.00 0 . .% 0.0000

05 - 02 0.70 1 0.402 0.0% 0.0000

05 - 01 3.79 2 0.150 47.2% 0.0243

03 - 01 0.00 1 0.980 0.0% 0.0000

04 - 03 4.55 4 0.336 12.2% 0.0276

04 - 02 0.29 2 0.864 0.0% 0.0000

16 - 10 0.00 0 . .% 0.0000

14 - 03 0.00 0 . .% 0.0000

17 - 02 3.32 1 0.068 69.9% 0.3337

08 - 04 0.00 0 . .% 0.0000

09 - 01 0.00 0 . .% 0.0000

05 - 03 3.02 2 0.221 33.7% 0.0161

09 - 03 0.00 0 . .% 0.0000

09 - 05 0.00 0 . .% 0.0000

16 - 01 0.00 0 . .% 0.0000

16 - 02 1.01 1 0.315 1.2% 0.0004

06 - 01 0.00 0 . .% 0.0000

11 - 02 0.00 0 . .% 0.0000

06 - 03 0.39 2 0.824 0.0% 0.0000

15 - 03 0.00 0 . .% 0.0000

07 - 04 0.00 0 . .% 0.0000

18 - 01 0.00 0 . .% 0.0000

08 - 05 0.00 0 . .% 0.0000

07 - 03 0.00 1 0.981 0.0% 0.0000

19 - 03 0.00 0 . .% 0.0000

** I-squared: the variation in ES attributable to heterogeneity

Significance test(s) of ES=1

02 - 01 z= 2.27 p = 0.023

12 - 01 z= 1.24 p = 0.216

13 - 01 z= 0.51 p = 0.610

12 - 02 z= 0.28 p = 0.780

13 - 02 z= 0.49 p = 0.625

13 - 12 z= 0.77 p = 0.442

05 - 02 z= 0.54 p = 0.586

05 - 01 z= 1.64 p = 0.102

03 - 01 z= 1.27 p = 0.203

04 - 03 z= 0.17 p = 0.864

04 - 02 z= 2.56 p = 0.010

16 - 10 z= 2.28 p = 0.023

14 - 03 z= 0.19 p = 0.851

17 - 02 z= 0.40 p = 0.686

08 - 04 z= 0.01 p = 0.989

09 - 01 z= 0.79 p = 0.432

05 - 03 z= 0.71 p = 0.480

09 - 03 z= 0.30 p = 0.764

09 - 05 z= 0.14 p = 0.889

16 - 01 z= 0.03 p = 0.973

16 - 02 z= 0.70 p = 0.485

06 - 01 z= 0.23 p = 0.820

11 - 02 z= 0.63 p = 0.531

06 - 03 z= 0.51 p = 0.608

15 - 03 z= 0.91 p = 0.362

07 - 04 z= 0.59 p = 0.553

18 - 01 z= 1.11 p = 0.267

08 - 05 z= 0.52 p = 0.606

07 - 03 z= 0.00 p = 0.998

19 - 03 z= 1.11 p = 0.266

## *Ischemic stroke*

Legend: 01=Placebo / No treatment; 02=Aspirin>150 mg; 03=Aspirin≤150 mg; 04=Clopidogrel 75 mg + Aspirin; 05=Dipyridamole + Aspirin; 06=Cilostazol 200 mg; 07=Cilostazol 200 mg + Aspirin; 08=Clopidogrel 75 mg; 09=Terutroban 30 mg; 10=Ticagrelor 180 mg; 11=Ticlopidine≤500 mg; 12=Ticlopidine<500 mg + Aspirin≤150 mg; 13=Triflusal 600 mg; 14=Vorapaxar 2.5 mg; 15=Ticagrelor 180 mg + Aspirin≤150 mg.

Study | Odds Ratio [95% Conf. Interval]

---------------------+---------------------------------------------------

02 - 01

SWEDISH CS 1987 | 0.958 0.557 1.649

EAFT 1993 | 0.786 0.544 1.138

CAST 1997 | 0.772 0.629 0.947

AICLA 1983 | 0.524 0.280 0.981

MAST-I 1995 | 3.079 0.124 76.163

Sub-total |

D+L pooled ES | 0.772 0.656 0.910

---------------------+---------------------------------------------------

03 - 01

SALT 1991 | 0.761 0.558 1.039

Sub-total |

D+L pooled ES | 0.761 0.558 1.039

---------------------+---------------------------------------------------

08 - 02

CAPRIE 1996 | 0.913 0.777 1.074

Sub-total |

D+L pooled ES | 0.913 0.777 1.074

---------------------+---------------------------------------------------

04 - 03

COMPRESS 2016 | 0.393 0.075 2.053

POINT 2018 | 0.714 0.557 0.917

CARESS 2005 | 0.113 0.006 2.157

CHARISMA 2011 | 0.792 0.597 1.050

CHANCE 2013 | 0.666 0.552 0.803

Sub-total |

D+L pooled ES | 0.700 0.614 0.799

---------------------+---------------------------------------------------

04 - 02

SPS3 2012 | 0.785 0.597 1.032

WANG 2015 | 0.267 0.098 0.729

YI 2014 | 0.267 0.098 0.729

Sub-total |

D+L pooled ES | 0.428 0.182 1.007

---------------------+---------------------------------------------------

09 - 03

PERFORM 2011 | 1.024 0.923 1.137

Sub-total |

D+L pooled ES | 1.024 0.923 1.137

---------------------+---------------------------------------------------

05 - 01

AICLA 1983 | 0.546 0.295 1.012

Sub-total |

D+L pooled ES | 0.546 0.295 1.012

---------------------+---------------------------------------------------

05 - 02

AICLA 1983 | 1.042 0.520 2.085

Sub-total |

D+L pooled ES | 1.042 0.520 2.085

---------------------+---------------------------------------------------

08 - 04

MATCH 2004 | 1.084 0.922 1.274

Sub-total |

D+L pooled ES | 1.084 0.922 1.274

---------------------+---------------------------------------------------

11 - 02

AAASPS 2003 | 1.274 0.942 1.723

TASS 1989 | 0.743 0.584 0.945

THOGI 1984 | 0.333 0.066 1.676

Sub-total |

D+L pooled ES | 0.882 0.528 1.473

---------------------+---------------------------------------------------

06 - 01

GOTOH 2000 | 0.499 0.315 0.790

Sub-total |

D+L pooled ES | 0.499 0.315 0.790

---------------------+---------------------------------------------------

05 - 03

ESPRIT 2006 | 0.823 0.621 1.091

JASAP 2011 | 1.406 0.882 2.243

Sub-total |

D+L pooled ES | 1.040 0.618 1.751

---------------------+---------------------------------------------------

13 - 08

MAESTRO 2017 | 1.648 0.851 3.191

Sub-total |

D+L pooled ES | 1.648 0.851 3.191

---------------------+---------------------------------------------------

06 - 03

CASISP 2008 | 0.723 0.327 1.596

CSPS2 2010 | 0.807 0.585 1.112

PICASSO 2018 | 0.714 0.469 1.087

Sub-total |

D+L pooled ES | 0.766 0.601 0.977

---------------------+---------------------------------------------------

12 - 11

TOPALS 2003 | 1.048 0.357 3.073

Sub-total |

D+L pooled ES | 1.048 0.357 3.073

---------------------+---------------------------------------------------

10 - 03

SOCRATES 2016 | 0.868 0.754 1.000

Sub-total |

D+L pooled ES | 0.868 0.754 1.000

---------------------+---------------------------------------------------

07 - 04

TOSS-2 2011 | 1.644 0.587 4.602

Sub-total |

D+L pooled ES | 1.644 0.587 4.602

---------------------+---------------------------------------------------

13 - 02

TACIP 2003 | 1.008 0.755 1.346

Sub-total |

D+L pooled ES | 1.008 0.755 1.346

---------------------+---------------------------------------------------

14 - 01

TIMI-50 2013 | 0.983 0.771 1.253

Sub-total |

D+L pooled ES | 0.983 0.771 1.253

---------------------+---------------------------------------------------

08 - 05

PRoFESS 2008 | 1.028 0.928 1.139

Sub-total |

D+L pooled ES | 1.028 0.928 1.139

---------------------+---------------------------------------------------

11 - 08

UCHIYAMA 2008 | 0.974 0.546 1.739

FUKUUCHI 2008 | 0.871 0.431 1.762

Sub-total |

D+L pooled ES | 0.931 0.595 1.457

---------------------+---------------------------------------------------

06 - 02

CAIST 2011 | 0.536 0.177 1.624

Sub-total |

D+L pooled ES | 0.536 0.177 1.624

---------------------+---------------------------------------------------

07 - 03

CATHARSIS 2015 | 0.624 0.169 2.301

ECLIPSE 2013 | 1.030 0.064 16.700

Sub-total |

D+L pooled ES | 0.683 0.210 2.226

---------------------+---------------------------------------------------

15 - 03

THALES 2019 | 0.785 0.667 0.924

Sub-total |

D+L pooled ES | 0.785 0.667 0.924

---------------------+---------------------------------------------------

Test(s) of heterogeneity:

Heterogeneity degrees of

statistic freedom P I-squared** Tau-squared

02 - 01 2.80 4 0.593 0.0% 0.0000

03 - 01 0.00 0 . .% 0.0000

08 - 02 0.00 0 . .% 0.0000

04 - 03 2.97 4 0.563 0.0% 0.0000

04 - 02 7.71 2 0.021 74.1% 0.4156

09 - 03 0.00 0 . .% 0.0000

05 - 01 0.00 0 . .% 0.0000

05 - 02 0.00 0 . .% 0.0000

08 - 04 0.00 0 . .% 0.0000

11 - 02 8.99 2 0.011 77.7% 0.1301

06 - 01 0.00 0 . .% 0.0000

05 - 03 3.71 1 0.054 73.0% 0.1048

13 - 08 0.00 0 . .% 0.0000

06 - 03 0.23 2 0.893 0.0% 0.0000

12 - 11 0.00 0 . .% 0.0000

10 - 03 0.00 0 . .% 0.0000

07 - 04 0.00 0 . .% 0.0000

13 - 02 0.00 0 . .% 0.0000

14 - 01 0.00 0 . .% 0.0000

08 - 05 0.00 0 . .% 0.0000

11 - 08 0.06 1 0.810 0.0% 0.0000

06 - 02 0.00 0 . .% 0.0000

07 - 03 0.10 1 0.750 0.0% 0.0000

15 - 03 0.00 0 . .% 0.0000

** I-squared: the variation in ES attributable to heterogeneity

Significance test(s) of ES=1

02 - 01 z= 3.10 p = 0.002

03 - 01 z= 1.72 p = 0.085

08 - 02 z= 1.10 p = 0.273

04 - 03 z= 5.30 p = 0.000

04 - 02 z= 1.94 p = 0.052

09 - 03 z= 0.45 p = 0.651

05 - 01 z= 1.92 p = 0.054

05 - 02 z= 0.12 p = 0.908

08 - 04 z= 0.97 p = 0.331

11 - 02 z= 0.48 p = 0.632

06 - 01 z= 2.96 p = 0.003

05 - 03 z= 0.15 p = 0.882

13 - 08 z= 1.48 p = 0.138

06 - 03 z= 2.15 p = 0.032

12 - 11 z= 0.09 p = 0.932

10 - 03 z= 1.96 p = 0.050

07 - 04 z= 0.95 p = 0.344

13 - 02 z= 0.05 p = 0.958

14 - 01 z= 0.14 p = 0.889

08 - 05 z= 0.53 p = 0.596

11 - 08 z= 0.31 p = 0.755

06 - 02 z= 1.10 p = 0.270

07 - 03 z= 0.63 p = 0.527

15 - 03 z= 2.91 p = 0.004

## *Ischemic stroke or transient ischemic attack*

Legend: 01=Placebo / No treatment; 02=Aspirin>150 mg; 03=Aspirin≤150 mg; 04=Clopidogrel 75 mg + Aspirin; 05=Dipyridamole + Aspirin; 06=Cilostazol 200 mg; 07=Clopidogrel 75 mg; 08=Satigrel; 09=Ticlopidine≤500 mg; 10=Ticlopidine<500 mg + Aspirin≤150 mg.

Study | Odds Ratio [95% Conf. Interval]

---------------------+---------------------------------------------------

02 - 01

SWEDISH CS 1987 | 0.995 0.614 1.613

Sub-total |

D+L pooled ES | 0.995 0.614 1.613

---------------------+---------------------------------------------------

04 - 02

SPS3 2012 | 0.758 0.594 0.966

Sub-total |

D+L pooled ES | 0.758 0.594 0.966

---------------------+---------------------------------------------------

06 - 01

GOTOH 2000 | 0.502 0.323 0.781

Sub-total |

D+L pooled ES | 0.502 0.323 0.781

---------------------+---------------------------------------------------

10 - 09

TOPALS 2003 | 0.946 0.388 2.308

Sub-total |

D+L pooled ES | 0.946 0.388 2.308

---------------------+---------------------------------------------------

04 - 03

CARESS 2005 | 0.399 0.130 1.224

CHANCE 2013 | 0.681 0.572 0.811

Sub-total |

D+L pooled ES | 0.673 0.566 0.799

---------------------+---------------------------------------------------

08 - 02

JETS-1 1995 | 0.411 0.193 0.877

Sub-total |

D+L pooled ES | 0.411 0.193 0.877

---------------------+---------------------------------------------------

06 - 03

CSPS2 2010 | 0.822 0.611 1.107

Sub-total |

D+L pooled ES | 0.822 0.611 1.107

---------------------+---------------------------------------------------

09 - 07

UCHIYAMA 2008 | 1.057 0.618 1.807

FUKUUCHI 2008 | 0.991 0.519 1.892

Sub-total |

D+L pooled ES | 1.029 0.681 1.556

---------------------+---------------------------------------------------

05 - 03

JASAP 2011 | 1.371 0.874 2.151

Sub-total |

D+L pooled ES | 1.371 0.874 2.151

---------------------+---------------------------------------------------

Test(s) of heterogeneity:

Heterogeneity degrees of

statistic freedom P I-squared** Tau-squared

02 - 01 0.00 0 . .% 0.0000

04 - 02 0.00 0 . .% 0.0000

06 - 01 0.00 0 . .% 0.0000

10 - 09 0.00 0 . .% 0.0000

04 - 03 0.86 1 0.355 0.0% 0.0000

08 - 02 0.00 0 . .% 0.0000

06 - 03 0.00 0 . .% 0.0000

09 - 07 0.02 1 0.881 0.0% 0.0000

05 - 03 0.00 0 . .% 0.0000

** I-squared: the variation in ES attributable to heterogeneity

Significance test(s) of ES=1

02 - 01 z= 0.02 p = 0.985

04 - 02 z= 2.24 p = 0.025

06 - 01 z= 3.06 p = 0.002

10 - 09 z= 0.12 p = 0.904

04 - 03 z= 4.51 p = 0.000

08 - 02 z= 2.30 p = 0.021

06 - 03 z= 1.29 p = 0.196

09 - 07 z= 0.14 p = 0.890

05 - 03 z= 1.38 p = 0.169

## *Cardiovascular event*

Legend: 01=Placebo / No treatment; 02=Aspirin>150 mg; 03=Aspirin≤150 mg; 04=Clopidogrel 75 mg + Aspirin; 05=Dipyridamole + Aspirin; 06=Cilostazol 200 mg; 07=Cilostazol 200 mg + Aspirin; 08=Clopidogrel 75 mg; 09=Dipyridamole>200 mg; 10=Indobufen 200 mg; 11=Piracetam 4800 mg; 12=Sulfinpyrazone 800 mg + Aspirin>150 mg; 13=Sulfinpyrazone 800 mg; 14=Terutroban 30 mg; 15=Ticagrelor 180 mg; 16=Ticlopidine≤500 mg; 17=Ticlopidine<500 mg + Aspirin≤150 mg; 18=Triflusal 600 mg; 19=Vorapaxar 2.5 mg.

Study | Odds Ratio [95% Conf. Interval]

---------------------+---------------------------------------------------

02 - 01

CANADIAN CS 1978 | 0.776 0.311 1.934

EAFT 1993 | 0.844 0.628 1.135

CAST 1997 | 0.892 0.760 1.047

AICLA 1983 | 0.483 0.284 0.821

UK-TIA 1991 | 0.843 0.688 1.034

MAST-I 1995 | 4.161 0.460 37.660

Sub-total |

D+L pooled ES | 0.832 0.714 0.969

---------------------+---------------------------------------------------

12 - 01

CANADIAN CS 1978 | 0.499 0.179 1.387

Sub-total |

D+L pooled ES | 0.499 0.179 1.387

---------------------+---------------------------------------------------

13 - 01

CANADIAN CS 1978 | 0.712 0.286 1.774

Sub-total |

D+L pooled ES | 0.712 0.286 1.774

---------------------+---------------------------------------------------

12 - 02

CANADIAN CS 1978 | 0.643 0.223 1.855

Sub-total |

D+L pooled ES | 0.643 0.223 1.855

---------------------+---------------------------------------------------

13 - 02

CANADIAN CS 1978 | 0.918 0.354 2.382

Sub-total |

D+L pooled ES | 0.918 0.354 2.382

---------------------+---------------------------------------------------

13 - 12

CANADIAN CS 1978 | 1.429 0.496 4.118

Sub-total |

D+L pooled ES | 1.429 0.496 4.118

---------------------+---------------------------------------------------

05 - 02

PERSANTIN ASPIRIN TR | 0.912 0.648 1.283

AICLA 1983 | 0.977 0.540 1.768

Sub-total |

D+L pooled ES | 0.928 0.690 1.247

---------------------+---------------------------------------------------

05 - 01

ESPS 1987 | 0.602 0.490 0.740

AICLA 1983 | 0.472 0.278 0.802

ESPS2 1996 | 0.624 0.515 0.755

Sub-total |

D+L pooled ES | 0.603 0.527 0.691

---------------------+---------------------------------------------------

03 - 01

SALT 1991 | 0.818 0.641 1.044

ESPS2 1996 | 0.841 0.702 1.007

Sub-total |

D+L pooled ES | 0.833 0.720 0.963

---------------------+---------------------------------------------------

08 - 02

CAPRIE 1996 | 0.918 0.797 1.058

Sub-total |

D+L pooled ES | 0.918 0.797 1.058

---------------------+---------------------------------------------------

04 - 03

COMPRESS 2016 | 0.658 0.182 2.377

POINT 2018 | 0.782 0.617 0.991

CARESS 2005 | 0.260 0.028 2.407

CHARISMA 2011 | 0.829 0.671 1.024

CHANCE 2013 | 0.677 0.564 0.813

Sub-total |

D+L pooled ES | 0.746 0.663 0.841

---------------------+---------------------------------------------------

04 - 02

SPS3 2012 | 0.857 0.681 1.078

Sub-total |

D+L pooled ES | 0.857 0.681 1.078

---------------------+---------------------------------------------------

16 - 10

TISS 1997 | 0.489 0.296 0.808

Sub-total |

D+L pooled ES | 0.489 0.296 0.808

---------------------+---------------------------------------------------

14 - 03

PERFORM 2011 | 1.029 0.941 1.126

Sub-total |

D+L pooled ES | 1.029 0.941 1.126

---------------------+---------------------------------------------------

18 - 02

TAPIRSS 2004 | 0.941 0.545 1.624

TACIP 2003 | 1.067 0.826 1.379

Sub-total |

D+L pooled ES | 1.043 0.827 1.316

---------------------+---------------------------------------------------

08 - 04

MATCH 2004 | 1.070 0.932 1.229

Sub-total |

D+L pooled ES | 1.070 0.932 1.229

---------------------+---------------------------------------------------

09 - 01

ESPS2 1996 | 0.857 0.716 1.025

Sub-total |

D+L pooled ES | 0.857 0.716 1.025

---------------------+---------------------------------------------------

05 - 03

ESPS2 1996 | 0.742 0.610 0.903

ESPRIT 2006 | 0.791 0.624 1.002

JASAP 2011 | 1.085 0.753 1.564

Sub-total |

D+L pooled ES | 0.819 0.680 0.987

---------------------+---------------------------------------------------

09 - 03

ESPS2 1996 | 1.019 0.847 1.226

Sub-total |

D+L pooled ES | 1.019 0.847 1.226

---------------------+---------------------------------------------------

09 - 05

ESPS2 1996 | 1.374 1.130 1.670

Sub-total |

D+L pooled ES | 1.374 1.130 1.670

---------------------+---------------------------------------------------

16 - 01

CATS 1989 | 0.744 0.557 0.994

Sub-total |

D+L pooled ES | 0.744 0.557 0.994

---------------------+---------------------------------------------------

16 - 02

AAASPS 2003 | 1.280 0.980 1.672

TASS 1989 | 0.887 0.738 1.067

THOGI 1984 | 0.523 0.216 1.269

Sub-total |

D+L pooled ES | 0.966 0.679 1.376

---------------------+---------------------------------------------------

06 - 01

GOTOH 2000 | 0.646 0.427 0.979

Sub-total |

D+L pooled ES | 0.646 0.427 0.979

---------------------+---------------------------------------------------

11 - 02

GROTEMEYER 2000 | 1.469 0.918 2.350

Sub-total |

D+L pooled ES | 1.469 0.918 2.350

---------------------+---------------------------------------------------

17 - 16

TOPALS 2003 | 1.049 0.403 2.729

Sub-total |

D+L pooled ES | 1.049 0.403 2.729

---------------------+---------------------------------------------------

15 - 03

SOCRATES 2016 | 0.884 0.774 1.010

Sub-total |

D+L pooled ES | 0.884 0.774 1.010

---------------------+---------------------------------------------------

07 - 04

TOSS-2 2011 | 1.486 0.653 3.381

Sub-total |

D+L pooled ES | 1.486 0.653 3.381

---------------------+---------------------------------------------------

19 - 01

TIMI-50 2013 | 1.027 0.841 1.255

Sub-total |

D+L pooled ES | 1.027 0.841 1.255

---------------------+---------------------------------------------------

08 - 05

PRoFESS 2008 | 1.003 0.925 1.089

Sub-total |

D+L pooled ES | 1.003 0.925 1.089

---------------------+---------------------------------------------------

06 - 03

PICASSO 2018 | 0.770 0.545 1.090

Sub-total |

D+L pooled ES | 0.770 0.545 1.090

---------------------+---------------------------------------------------

16 - 08

FUKUUCHI 2008 | 0.871 0.431 1.762

Sub-total |

D+L pooled ES | 0.871 0.431 1.762

---------------------+---------------------------------------------------

06 - 02

CAIST 2011 | 0.646 0.226 1.845

Sub-total |

D+L pooled ES | 0.646 0.226 1.845

---------------------+---------------------------------------------------

07 - 03

CATHARSIS 2015 | 0.402 0.133 1.215

Sub-total |

D+L pooled ES | 0.402 0.133 1.215

---------------------+---------------------------------------------------

Test(s) of heterogeneity:

Heterogeneity degrees of

statistic freedom P I-squared** Tau-squared

02 - 01 6.75 5 0.240 25.9% 0.0089

12 - 01 0.00 0 . .% 0.0000

13 - 01 0.00 0 . .% 0.0000

12 - 02 0.00 0 . .% 0.0000

13 - 02 0.00 0 . .% 0.0000

13 - 12 0.00 0 . .% 0.0000

05 - 02 0.04 1 0.842 0.0% 0.0000

05 - 01 0.94 2 0.625 0.0% 0.0000

03 - 01 0.03 1 0.860 0.0% 0.0000

08 - 02 0.00 0 . .% 0.0000

04 - 03 3.08 4 0.544 0.0% 0.0000

04 - 02 0.00 0 . .% 0.0000

16 - 10 0.00 0 . .% 0.0000

14 - 03 0.00 0 . .% 0.0000

18 - 02 0.17 1 0.681 0.0% 0.0000

08 - 04 0.00 0 . .% 0.0000

09 - 01 0.00 0 . .% 0.0000

05 - 03 3.25 2 0.197 38.5% 0.0106

09 - 03 0.00 0 . .% 0.0000

09 - 05 0.00 0 . .% 0.0000

16 - 01 0.00 0 . .% 0.0000

16 - 02 6.87 2 0.032 70.9% 0.0608

06 - 01 0.00 0 . .% 0.0000

11 - 02 0.00 0 . .% 0.0000

17 - 16 0.00 0 . .% 0.0000

15 - 03 0.00 0 . .% 0.0000

07 - 04 0.00 0 . .% 0.0000

19 - 01 0.00 0 . .% 0.0000

08 - 05 0.00 0 . .% 0.0000

06 - 03 0.00 0 . .% 0.0000

16 - 08 0.00 0 . .% 0.0000

06 - 02 0.00 0 . .% 0.0000

07 - 03 0.00 0 . .% 0.0000

** I-squared: the variation in ES attributable to heterogeneity)

Significance test(s) of ES=1

02 - 01 z= 2.36 p = 0.018

12 - 01 z= 1.33 p = 0.183

13 - 01 z= 0.73 p = 0.466

12 - 02 z= 0.82 p = 0.414

13 - 02 z= 0.18 p = 0.861

13 - 12 z= 0.66 p = 0.509

05 - 02 z= 0.50 p = 0.619

05 - 01 z= 7.31 p = 0.000

03 - 01 z= 2.47 p = 0.013

08 - 02 z= 1.18 p = 0.236

04 - 03 z= 4.83 p = 0.000

04 - 02 z= 1.32 p = 0.188

16 - 10 z= 2.79 p = 0.005

14 - 03 z= 0.63 p = 0.527

18 - 02 z= 0.36 p = 0.721

08 - 04 z= 0.96 p = 0.335

09 - 01 z= 1.69 p = 0.091

05 - 03 z= 2.10 p = 0.036

09 - 03 z= 0.20 p = 0.843

09 - 05 z= 3.18 p = 0.001

16 - 01 z= 2.00 p = 0.045

16 - 02 z= 0.19 p = 0.850

06 - 01 z= 2.06 p = 0.039

11 - 02 z= 1.60 p = 0.109

17 - 16 z= 0.10 p = 0.922

15 - 03 z= 1.81 p = 0.070

07 - 04 z= 0.94 p = 0.345

19 - 01 z= 0.26 p = 0.794

08 - 05 z= 0.08 p = 0.935

06 - 03 z= 1.47 p = 0.140

16 - 08 z= 0.38 p = 0.702

06 - 02 z= 0.82 p = 0.414

07 - 03 z= 1.62 p = 0.106

## *Hemorrhagic stroke*

Legend: 01=Placebo / No treatment; 02=Aspirin>150 mg; 03=Aspirin≤150 mg; 04=Clopidogrel 75 mg + Aspirin; 05=Dipyridamole + Aspirin; 06=Cilostazol 200 mg; 07=Cilostazol 200 mg + Aspirin; 08=Clopidogrel 75 mg; 09=Terutroban 30 mg; 10=Ticagrelor 180 mg; 11=Ticlopidine≤500 mg; 12=Ticlopidine<500 mg + Aspirin≤150 mg; 13=Triflusal 600 mg; 14=Vorapaxar 2.5 mg; 15=Ticagrelor 180 mg + Aspirin≤150 mg.

Study | ES [95% Conf. Interval]

---------------------+---------------------------------------------------

05 - 02

PERSANTIN ASPIRIN TR | 0.491 0.089 2.695

Sub-total |

D+L pooled ES | 0.491 0.089 2.695

---------------------+---------------------------------------------------

02 - 01

SWEDISH CS 1987 | 1.500 0.249 9.054

EAFT 1993 | 2.814 0.114 69.292

CAST 1997 | 1.237 0.940 1.629

MAST-I 1995 | 3.100 0.319 30.135

Sub-total |

D+L pooled ES | 1.266 0.968 1.656

---------------------+---------------------------------------------------

03 - 01

SALT 1991 | 3.408 0.934 12.439

Sub-total |

D+L pooled ES | 3.408 0.934 12.439

---------------------+---------------------------------------------------

04 - 03

COMPRESS 2016 | 3.018 0.122 74.618

POINT 2018 | 1.680 0.401 7.036

CARESS 2005 | 1.097 0.021 56.302

CHARISMA 2011 | 1.115 0.452 2.749

CHANCE 2013 | 1.001 0.375 2.671

Sub-total |

D+L pooled ES | 1.187 0.661 2.133

---------------------+---------------------------------------------------

04 - 02

SPS3 2012 | 0.526 0.222 1.244

WANG 2015 | 1.007 0.063 16.179

YI 2014 | 0.912 0.381 2.183

Sub-total |

D+L pooled ES | 0.702 0.386 1.278

---------------------+---------------------------------------------------

09 - 03

PERFORM 2011 | 1.155 0.812 1.643

Sub-total |

D+L pooled ES | 1.155 0.812 1.643

---------------------+---------------------------------------------------

08 - 04

MATCH 2004 | 0.622 0.376 1.027

Sub-total |

D+L pooled ES | 0.622 0.376 1.027

---------------------+---------------------------------------------------

11 - 01

CATS 1989 | 5.048 0.242 105.395

Sub-total |

D+L pooled ES | 5.048 0.242 105.395

---------------------+---------------------------------------------------

13 - 08

MAESTRO 2017 | 0.399 0.077 2.069

Sub-total |

D+L pooled ES | 0.399 0.077 2.069

---------------------+---------------------------------------------------

11 - 02

TASS 1989 | 1.007 0.352 2.878

Sub-total |

D+L pooled ES | 1.007 0.352 2.878

---------------------+---------------------------------------------------

06 - 03

CASISP 2008 | 0.140 0.017 1.144

PICASSO 2018 | 0.495 0.221 1.110

Sub-total |

D+L pooled ES | 0.388 0.146 1.031

---------------------+---------------------------------------------------

12 - 11

TOPALS 2003 | 5.307 0.252 111.575

Sub-total |

D+L pooled ES | 5.307 0.252 111.575

---------------------+---------------------------------------------------

10 - 03

SOCRATES 2016 | 0.557 0.187 1.663

Sub-total |

D+L pooled ES | 0.557 0.187 1.663

---------------------+---------------------------------------------------

07 - 04

TOSS-2 2011 | 2.922 0.118 72.111

Sub-total |

D+L pooled ES | 2.922 0.118 72.111

---------------------+---------------------------------------------------

14 - 01

TIMI-50 2013 | 2.847 1.575 5.147

Sub-total |

D+L pooled ES | 2.847 1.575 5.147

---------------------+---------------------------------------------------

08 - 05

PRoFESS 2008 | 0.611 0.436 0.855

Sub-total |

D+L pooled ES | 0.611 0.436 0.855

---------------------+---------------------------------------------------

05 - 03

JASAP 2011 | 1.479 0.600 3.642

Sub-total |

D+L pooled ES | 1.479 0.600 3.642

---------------------+---------------------------------------------------

11 - 08

FUKUUCHI 2008 | 0.329 0.034 3.175

Sub-total |

D+L pooled ES | 0.329 0.034 3.175

---------------------+---------------------------------------------------

07 - 03

CATHARSIS 2015 | 0.317 0.013 7.906

ECLIPSE 2013 | 1.030 0.020 52.402

Sub-total |

D+L pooled ES | 0.509 0.042 6.128

---------------------+---------------------------------------------------

15 - 03

THALES 2019 | 4.980 1.091 22.739

Sub-total |

D+L pooled ES | 4.980 1.091 22.739

---------------------+---------------------------------------------------

Test(s) of heterogeneity:

Heterogeneity degrees of

statistic freedom P I-squared** Tau-squared

05 - 02 0.00 0 . .% 0.0000

02 - 01 0.90 3 0.827 0.0% 0.0000

03 - 01 0.00 0 . .% 0.0000

04 - 03 0.69 4 0.953 0.0% 0.0000

04 - 02 0.84 2 0.656 0.0% 0.0000

09 - 03 0.00 0 . .% 0.0000

08 - 04 0.00 0 . .% 0.0000

11 - 01 0.00 0 . .% 0.0000

13 - 08 0.00 0 . .% 0.0000

11 - 02 0.00 0 . .% 0.0000

06 - 03 1.21 1 0.271 17.4% 0.1387

12 - 11 0.00 0 . .% 0.0000

10 - 03 0.00 0 . .% 0.0000

07 - 04 0.00 0 . .% 0.0000

14 - 01 0.00 0 . .% 0.0000

08 - 05 0.00 0 . .% 0.0000

05 - 03 0.00 0 . .% 0.0000

11 - 08 0.00 0 . .% 0.0000

07 - 03 0.21 1 0.650 0.0% 0.0000

15 - 03 0.00 0 . .% 0.0000

** I-squared: the variation in ES attributable to heterogeneity)

Significance test(s) of ES=1

05 - 02 z= 0.82 p = 0.413

02 - 01 z= 1.72 p = 0.085

03 - 01 z= 1.86 p = 0.063

04 - 03 z= 0.57 p = 0.566

04 - 02 z= 1.16 p = 0.247

09 - 03 z= 0.80 p = 0.424

08 - 04 z= 1.86 p = 0.063

11 - 01 z= 1.04 p = 0.296

13 - 08 z= 1.09 p = 0.274

11 - 02 z= 0.01 p = 0.989

06 - 03 z= 1.90 p = 0.058

12 - 11 z= 1.07 p = 0.283

10 - 03 z= 1.05 p = 0.294

07 - 04 z= 0.66 p = 0.512

14 - 01 z= 3.46 p = 0.001

08 - 05 z= 2.87 p = 0.004

05 - 03 z= 0.85 p = 0.395

11 - 08 z= 0.96 p = 0.337

07 - 03 z= 0.53 p = 0.595

15 - 03 z= 2.07 p = 0.038

-------------------------------------------------------------------------

## *Intracranial haemorrhage*

Legend: 01=Placebo / No treatment; 02=Aspirin>150 mg; 03=Aspirin≤150 mg; 04=Clopidogrel 75 mg + Aspirin; 05=Dipyridamole + Aspirin; 06=Cilostazol 200 mg; 07=Cilostazol 200 mg + Aspirin; 08=Clopidogrel 75 mg; 09=Satigrel; 10=Terutroban 30 mg; 11=Ticagrelor 180 mg; 12=Ticlopidine≤500 mg; 13=Ticlopidine<500 mg + Aspirin≤150 mg; 14=Triflusal 600 mg; 15=Vorapaxar 2.5 mg; 16=Ticagrelor 180 mg + Aspirin≤150 mg.

Study | Odds Ratio [95% Conf. Interval]

---------------------+---------------------------------------------------

05 - 02

PERSANTIN ASPIRIN TR | 0.392 0.076 2.031

AICLA 1983 | 0.488 0.044 5.420

Sub-total |

D+L pooled ES | 0.420 0.108 1.634

---------------------+---------------------------------------------------

03 - 01

SALT 1991 | 3.408 0.934 12.439

Sub-total |

D+L pooled ES | 3.408 0.934 12.439

---------------------+---------------------------------------------------

04 - 03

COMPRESS 2016 | 5.060 0.241 106.208

POINT 2018 | 1.816 0.608 5.425

CARESS 2005 | 1.097 0.021 56.302

CHARISMA 2011 | 1.186 0.530 2.654

Sub-total |

D+L pooled ES | 1.447 0.774 2.707

---------------------+---------------------------------------------------

04 - 02

SPS3 2012 | 0.672 0.347 1.301

WANG 2015 | 1.007 0.063 16.179

YI 2014 | 1.007 0.063 16.179

Sub-total |

D+L pooled ES | 0.700 0.375 1.310

---------------------+---------------------------------------------------

10 - 03

PERFORM 2011 | 1.208 0.948 1.540

Sub-total |

D+L pooled ES | 1.208 0.948 1.540

---------------------+---------------------------------------------------

02 - 01

AICLA 1983 | 1.031 0.144 7.389

Sub-total |

D+L pooled ES | 1.031 0.144 7.389

---------------------+---------------------------------------------------

05 - 01

AICLA 1983 | 0.502 0.045 5.586

Sub-total |

D+L pooled ES | 0.502 0.045 5.586

---------------------+---------------------------------------------------

08 - 04

MATCH 2004 | 0.578 0.394 0.848

Sub-total |

D+L pooled ES | 0.578 0.394 0.848

---------------------+---------------------------------------------------

12 - 01

CATS 1989 | 1.006 0.141 7.166

Sub-total |

D+L pooled ES | 1.006 0.141 7.166

---------------------+---------------------------------------------------

06 - 01

GOTOH 2000 | 0.569 0.166 1.956

Sub-total |

D+L pooled ES | 0.569 0.166 1.956

---------------------+---------------------------------------------------

05 - 03

ESPRIT 2006 | 0.573 0.281 1.170

JASAP 2011 | 0.980 0.451 2.130

Sub-total |

D+L pooled ES | 0.732 0.433 1.239

---------------------+---------------------------------------------------

14 - 08

MAESTRO 2017 | 0.426 0.109 1.661

Sub-total |

D+L pooled ES | 0.426 0.109 1.661

---------------------+---------------------------------------------------

13 - 12

TOPALS 2003 | 5.307 0.252 111.575

Sub-total |

D+L pooled ES | 5.307 0.252 111.575

---------------------+---------------------------------------------------

11 - 03

SOCRATES 2016 | 0.668 0.322 1.388

Sub-total |

D+L pooled ES | 0.668 0.322 1.388

---------------------+---------------------------------------------------

09 - 02

JETS-1 1995 | 0.176 0.007 4.376

Sub-total |

D+L pooled ES | 0.176 0.007 4.376

---------------------+---------------------------------------------------

14 - 02

TACIP 2003 | 0.632 0.244 1.637

Sub-total |

D+L pooled ES | 0.632 0.244 1.637

---------------------+---------------------------------------------------

15 - 01

TIMI-50 2013 | 2.542 1.467 4.404

Sub-total |

D+L pooled ES | 2.542 1.467 4.404

---------------------+---------------------------------------------------

08 - 05

PRoFESS 2008 | 0.700 0.543 0.901

Sub-total |

D+L pooled ES | 0.700 0.543 0.901

---------------------+---------------------------------------------------

06 - 03

CSPS2 2010 | 0.317 0.155 0.649

Sub-total |

D+L pooled ES | 0.317 0.155 0.649

---------------------+---------------------------------------------------

12 - 08

FUKUUCHI 2008 | 0.247 0.027 2.212

Sub-total |

D+L pooled ES | 0.247 0.027 2.212

---------------------+---------------------------------------------------

07 - 03

CATHARSIS 2015 | 0.188 0.009 3.978

Sub-total |

D+L pooled ES | 0.188 0.009 3.978

---------------------+---------------------------------------------------

16 - 03

THALES 2019 | 3.324 1.334 8.282

Sub-total |

D+L pooled ES | 3.324 1.334 8.282

---------------------+---------------------------------------------------

Test(s) of heterogeneity:

Heterogeneity degrees of

statistic freedom P I-squared** Tau-squared

05 - 02 0.02 1 0.883 0.0% 0.0000

03 - 01 0.00 0 . .% 0.0000

04 - 03 1.07 3 0.785 0.0% 0.0000

04 - 02 0.15 2 0.930 0.0% 0.0000

10 - 03 0.00 0 . .% 0.0000

02 - 01 0.00 0 . .% 0.0000

05 - 01 0.00 0 . .% 0.0000

08 - 04 0.00 0 . .% 0.0000

12 - 01 0.00 0 . .% 0.0000

06 - 01 0.00 0 . .% 0.0000

05 - 03 0.99 1 0.319 0.0% 0.0000

14 - 08 0.00 0 . .% 0.0000

13 - 12 0.00 0 . .% 0.0000

11 - 03 0.00 0 . .% 0.0000

09 - 02 0.00 0 . .% 0.0000

14 - 02 0.00 0 . .% 0.0000

15 - 01 0.00 0 . .% 0.0000

08 - 05 0.00 0 . .% 0.0000

06 - 03 0.00 0 . .% 0.0000

12 - 08 0.00 0 . .% 0.0000

07 - 03 0.00 0 . .% 0.0000

16 - 03 0.00 0 . .% 0.0000

** I-squared: the variation in ES attributable to heterogeneity)

Significance test(s) of ES=1

05 - 02 z= 1.25 p = 0.211

03 - 01 z= 1.86 p = 0.063

04 - 03 z= 1.16 p = 0.247

04 - 02 z= 1.12 p = 0.265

10 - 03 z= 1.53 p = 0.126

02 - 01 z= 0.03 p = 0.976

05 - 01 z= 0.56 p = 0.575

08 - 04 z= 2.80 p = 0.005

12 - 01 z= 0.01 p = 0.995

06 - 01 z= 0.89 p = 0.371

05 - 03 z= 1.16 p = 0.245

14 - 08 z= 1.23 p = 0.219

13 - 12 z= 1.07 p = 0.283

11 - 03 z= 1.08 p = 0.280

09 - 02 z= 1.06 p = 0.289

14 - 02 z= 0.94 p = 0.345

15 - 01 z= 3.33 p = 0.001

08 - 05 z= 2.76 p = 0.006

06 - 03 z= 3.14 p = 0.002

12 - 08 z= 1.25 p = 0.211

07 - 03 z= 1.07 p = 0.283

16 - 03 z= 2.58 p = 0.010

## *Major bleeding*

Legend: 01=Placebo / No treatment; 02=Aspirin>150 mg; 03=Aspirin≤150 mg; 04=Clopidogrel 75 mg + Aspirin; 05=Dipyridamole + Aspirin; 06=Cilostazol 200 mg; 07=Cilostazol 200 mg + Aspirin; 08=Clopidogrel 75 mg; 09=Dipyridamole>200 mg; 10=Piracetam 4800 mg; 11=Sulfinpyrazone 800 mg + Aspirin>150 mg; 12=Sulfinpyrazone 800 mg; 13=Terutroban 30 mg; 14=Ticagrelor 180 mg; 15=Ticlopidine≤500 mg; 16=Ticlopidine<500 mg + Aspirin≤150 mg; 17=Triflusal 600 mg; 18=Vorapaxar 2.5 mg; 19=Ticagrelor 180 mg + Aspirin≤150 mg.

Study | Odds Ratio [95% Conf. Interval]

---------------------+---------------------------------------------------

02 - 01

CANADIAN CS 1978 | 0.190 0.009 4.000

SWEDISH CS 1987 | 1.500 0.249 9.054

EAFT 1993 | 1.410 0.395 5.034

CAST 1997 | 1.336 1.080 1.653

AICLA 1983 | 5.372 1.162 24.838

UK-TIA 1991 | 6.573 0.858 50.332

MAST-I 1995 | 4.161 0.460 37.660

Sub-total |

D+L pooled ES | 1.752 1.009 3.042

---------------------+---------------------------------------------------

11 - 01

CANADIAN CS 1978 | 2.544 0.581 11.149

Sub-total |

D+L pooled ES | 2.544 0.581 11.149

---------------------+---------------------------------------------------

12 - 01

CANADIAN CS 1978 | 1.623 0.340 7.748

Sub-total |

D+L pooled ES | 1.623 0.340 7.748

---------------------+---------------------------------------------------

11 - 02

CANADIAN CS 1978 | 13.370 0.746 239.564

Sub-total |

D+L pooled ES | 13.370 0.746 239.564

---------------------+---------------------------------------------------

12 - 02

CANADIAN CS 1978 | 8.528 0.455 159.801

Sub-total |

D+L pooled ES | 8.528 0.455 159.801

---------------------+---------------------------------------------------

12 - 11

CANADIAN CS 1978 | 0.638 0.188 2.168

Sub-total |

D+L pooled ES | 0.638 0.188 2.168

---------------------+---------------------------------------------------

05 - 02

PERSANTIN ASPIRIN TR | 0.631 0.325 1.226

AICLA 1983 | 0.094 0.012 0.738

Sub-total |

D+L pooled ES | 0.315 0.052 1.907

---------------------+---------------------------------------------------

03 - 01

SALT 1991 | 2.287 1.034 5.058

ESPS2 1996 | 2.880 1.214 6.829

Sub-total |

D+L pooled ES | 2.542 1.417 4.560

---------------------+---------------------------------------------------

04 - 03

COMPRESS 2016 | 3.689 0.755 18.010

POINT 2018 | 2.329 1.106 4.903

CARESS 2005 | 1.097 0.021 56.302

CHARISMA 2011 | 1.113 0.711 1.743

CHANCE 2013 | 1.001 0.250 4.006

Sub-total |

D+L pooled ES | 1.457 0.955 2.224

---------------------+---------------------------------------------------

04 - 02

SPS3 2012 | 1.921 1.378 2.679

WANG 2015 | 1.522 0.425 5.450

YI 2014 | 1.007 0.063 16.179

Sub-total |

D+L pooled ES | 1.877 1.364 2.584

---------------------+---------------------------------------------------

13 - 03

PERFORM 2011 | 1.003 0.827 1.217

Sub-total |

D+L pooled ES | 1.003 0.827 1.217

---------------------+---------------------------------------------------

05 - 01

AICLA 1983 | 0.502 0.045 5.586

ESPS2 1996 | 3.902 1.695 8.986

Sub-total |

D+L pooled ES | 1.936 0.288 13.019

---------------------+---------------------------------------------------

17 - 02

TAPIRSS 2004 | 0.140 0.017 1.149

TACIP 2003 | 0.465 0.271 0.797

Sub-total |

D+L pooled ES | 0.400 0.184 0.870

---------------------+---------------------------------------------------

08 - 04

MATCH 2004 | 0.407 0.307 0.538

Sub-total |

D+L pooled ES | 0.407 0.307 0.538

---------------------+---------------------------------------------------

09 - 01

ESPS2 1996 | 0.854 0.286 2.547

Sub-total |

D+L pooled ES | 0.854 0.286 2.547

---------------------+---------------------------------------------------

05 - 03

ESPS2 1996 | 1.355 0.757 2.426

ESPRIT 2006 | 0.658 0.426 1.015

JASAP 2011 | 1.059 0.602 1.865

Sub-total |

D+L pooled ES | 0.946 0.610 1.468

---------------------+---------------------------------------------------

09 - 03

ESPS2 1996 | 0.297 0.119 0.740

Sub-total |

D+L pooled ES | 0.297 0.119 0.740

---------------------+---------------------------------------------------

09 - 05

ESPS2 1996 | 0.219 0.090 0.531

Sub-total |

D+L pooled ES | 0.219 0.090 0.531

---------------------+---------------------------------------------------

15 - 01

CATS 1989 | 1.344 0.299 6.033

Sub-total |

D+L pooled ES | 1.344 0.299 6.033

---------------------+---------------------------------------------------

15 - 02

AAASPS 2003 | 0.524 0.242 1.133

Sub-total |

D+L pooled ES | 0.524 0.242 1.133

---------------------+---------------------------------------------------

06 - 01

GOTOH 2000 | 0.570 0.166 1.960

Sub-total |

D+L pooled ES | 0.570 0.166 1.960

---------------------+---------------------------------------------------

10 - 02

GROTEMEYER 2000 | 0.147 0.018 1.180

Sub-total |

D+L pooled ES | 0.147 0.018 1.180

---------------------+---------------------------------------------------

17 - 08

MAESTRO 2017 | 0.426 0.109 1.661

Sub-total |

D+L pooled ES | 0.426 0.109 1.661

---------------------+---------------------------------------------------

16 - 15

TOPALS 2003 | 1.048 0.329 3.334

Sub-total |

D+L pooled ES | 1.048 0.329 3.334

---------------------+---------------------------------------------------

14 - 03

SOCRATES 2016 | 0.818 0.508 1.315

Sub-total |

D+L pooled ES | 0.818 0.508 1.315

---------------------+---------------------------------------------------

07 - 04

TOSS-2 2011 | 0.317 0.063 1.589

Sub-total |

D+L pooled ES | 0.317 0.063 1.589

---------------------+---------------------------------------------------

18 - 01

TIMI-50 2013 | 2.114 1.319 3.386

Sub-total |

D+L pooled ES | 2.114 1.319 3.386

---------------------+---------------------------------------------------

08 - 05

PRoFESS 2008 | 0.869 0.753 1.003

Sub-total |

D+L pooled ES | 0.869 0.753 1.003

---------------------+---------------------------------------------------

06 - 03

CSPS2 2010 | 0.392 0.240 0.641

Sub-total |

D+L pooled ES | 0.392 0.240 0.641

---------------------+---------------------------------------------------

15 - 08

FUKUUCHI 2008 | 0.616 0.200 1.895

Sub-total |

D+L pooled ES | 0.616 0.200 1.895

---------------------+---------------------------------------------------

06 - 02

CAIST 2011 | 0.388 0.074 2.019

Sub-total |

D+L pooled ES | 0.388 0.074 2.019

---------------------+---------------------------------------------------

07 - 03

CATHARSIS 2015 | 1.300 0.282 5.999

ECLIPSE 2013 | 1.030 0.020 52.402

Sub-total |

D+L pooled ES | 1.260 0.303 5.242

---------------------+---------------------------------------------------

19 - 03

THALES 2019 | 3.993 1.743 9.150

Sub-total |

D+L pooled ES | 3.993 1.743 9.150

---------------------+---------------------------------------------------

Test(s) of heterogeneity:

Heterogeneity degrees of

statistic freedom P I-squared** Tau-squared

02 - 01 7.96 6 0.241 24.6% 0.1406

11 - 01 0.00 0 . .% 0.0000

12 - 01 0.00 0 . .% 0.0000

11 - 02 0.00 0 . .% 0.0000

12 - 02 0.00 0 . .% 0.0000

12 - 11 0.00 0 . .% 0.0000

05 - 02 2.98 1 0.085 66.4% 1.2098

03 - 01 0.15 1 0.700 0.0% 0.0000

04 - 03 4.47 4 0.346 10.5% 0.0299

04 - 02 0.32 2 0.854 0.0% 0.0000

13 - 03 0.00 0 . .% 0.0000

05 - 01 2.48 1 0.115 59.8% 1.2552

17 - 02 1.17 1 0.279 14.5% 0.1041

08 - 04 0.00 0 . .% 0.0000

09 - 01 0.00 0 . .% 0.0000

05 - 03 4.22 2 0.121 52.6% 0.0793

09 - 03 0.00 0 . .% 0.0000

09 - 05 0.00 0 . .% 0.0000

15 - 01 0.00 0 . .% 0.0000

15 - 02 0.00 0 . .% 0.0000

06 - 01 0.00 0 . .% 0.0000

10 - 02 0.00 0 . .% 0.0000

17 - 08 0.00 0 . .% 0.0000

16 - 15 0.00 0 . .% 0.0000

14 - 03 0.00 0 . .% 0.0000

07 - 04 0.00 0 . .% 0.0000

18 - 01 0.00 0 . .% 0.0000

08 - 05 0.00 0 . .% 0.0000

06 - 03 0.00 0 . .% 0.0000

15 - 08 0.00 0 . .% 0.0000

06 - 02 0.00 0 . .% 0.0000

07 - 03 0.01 1 0.914 0.0% 0.0000

19 - 03 0.00 0 . .% 0.0000

** I-squared: the variation in ES attributable to heterogeneity)

Significance test(s) of ES=1

02 - 01 z= 1.99 p = 0.046

11 - 01 z= 1.24 p = 0.215

12 - 01 z= 0.61 p = 0.544

11 - 02 z= 1.76 p = 0.078

12 - 02 z= 1.43 p = 0.152

12 - 11 z= 0.72 p = 0.471

05 - 02 z= 1.26 p = 0.209

03 - 01 z= 3.13 p = 0.002

04 - 03 z= 1.75 p = 0.081

04 - 02 z= 3.86 p = 0.000

13 - 03 z= 0.04 p = 0.972

05 - 01 z= 0.68 p = 0.497

17 - 02 z= 2.31 p = 0.021

08 - 04 z= 6.28 p = 0.000

09 - 01 z= 0.28 p = 0.777

05 - 03 z= 0.25 p = 0.806

09 - 03 z= 2.60 p = 0.009

09 - 05 z= 3.36 p = 0.001

15 - 01 z= 0.39 p = 0.700

15 - 02 z= 1.64 p = 0.101

06 - 01 z= 0.89 p = 0.373

10 - 02 z= 1.80 p = 0.071

17 - 08 z= 1.23 p = 0.219

16 - 15 z= 0.08 p = 0.937

14 - 03 z= 0.83 p = 0.406

07 - 04 z= 1.40 p = 0.163

18 - 01 z= 3.11 p = 0.002

08 - 05 z= 1.92 p = 0.054

06 - 03 z= 3.74 p = 0.000

15 - 08 z= 0.84 p = 0.398

06 - 02 z= 1.13 p = 0.261

07 - 03 z= 0.32 p = 0.750

19 - 03 z= 3.27 p = 0.001

# **Supplementary 7. Assessment of the confidence in the network estimates by outcome**

Legend for treatments: 1=Placebo/No treatment; 2=Aspirin>150 mg; 3=Aspirin≤150 mg; 4=Clopidogrel 75 mg + Aspirin; 5=Dipyridamole + Aspirin; 6=Cilostazol 200 mg; 7=Cilostazol 200 mg + Aspirin; 8=Clopidogrel 75 mg; 9=Dipyridamole>200 mg; 10=Indobufen 200 mg; 11= Picotamide 600 mg; 12=Piracetam 4800 mg; 13=Satigrel; 14=Sulfinpyrazone 800 mg + Aspirin>150 mg; 15=Sulfinpyrazone 800 mg; 16=Terutroban 30 mg; 17=Ticagrelor 180 mg; 18=Ticlopidine≤500 mg; 19=Ticlopidine<500 mg + Aspirin≤150 mg; 20=Triflusal 600 mg; 21=Vorapaxar 1-2.5 mg + Aspirin≤150 mg; 22=Vorapaxar 2.5 mg; 23=Ticagrelor 180 mg + Aspirin≤150 mg

**Confidence in the estimates for each drug by outcome (a: versus placebo/no treatment; b: versus aspirin ≤150 mg/day)**

## *All strokes*

Risk of bias bar chart


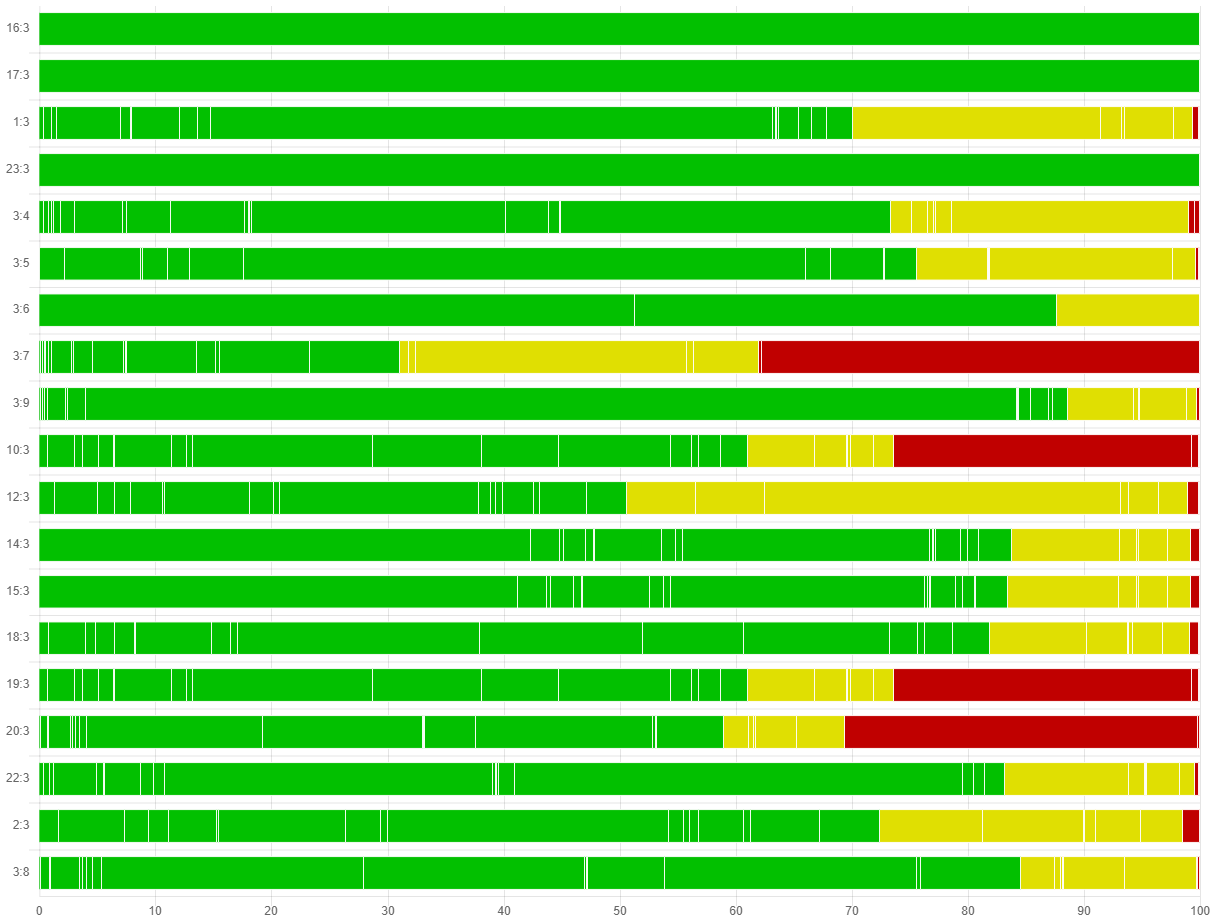


b) versus aspirin ≤150 mg/day


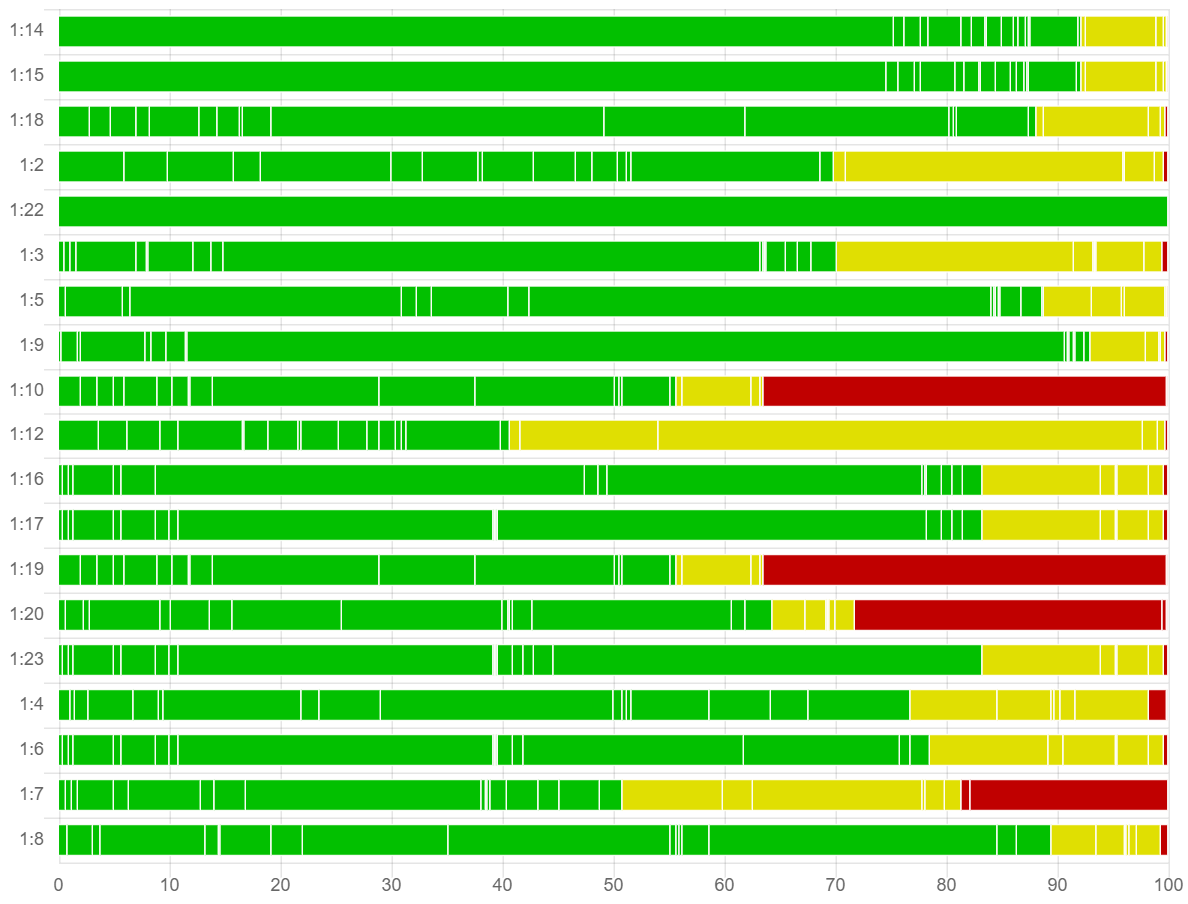


a) versus placebo/no treatment

Forest plot with network estimates and relative prediction intervals


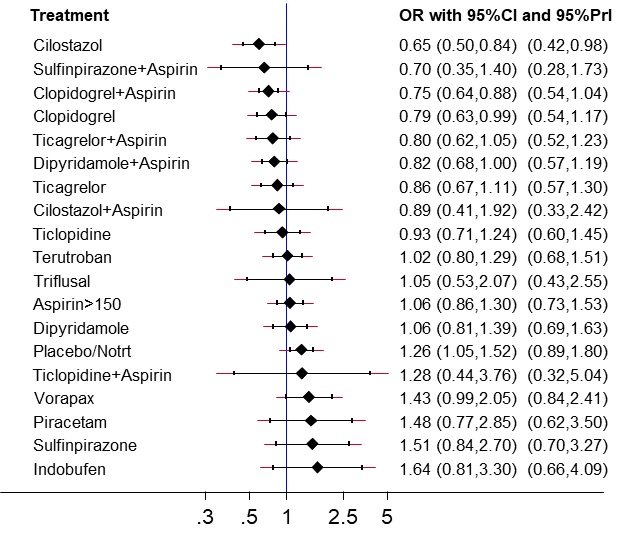


b) versus aspirin ≤150 mg/day


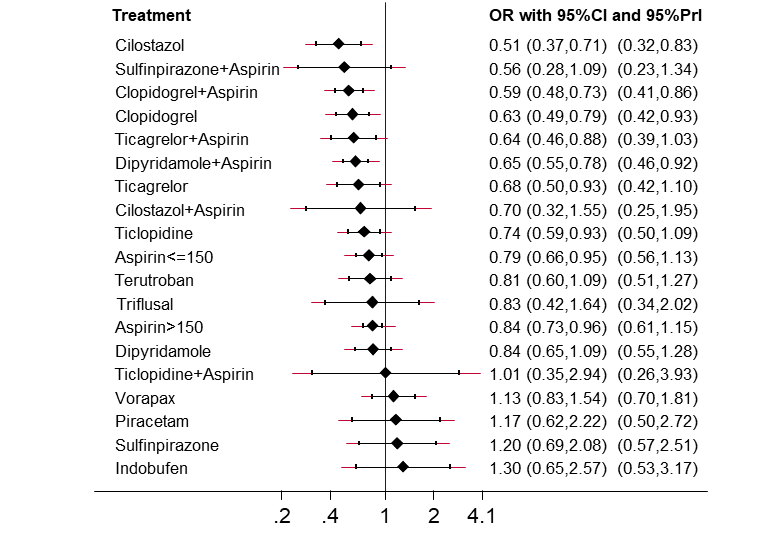


a) versus placebo/no treatment

Rating of the confidence in the network estimates


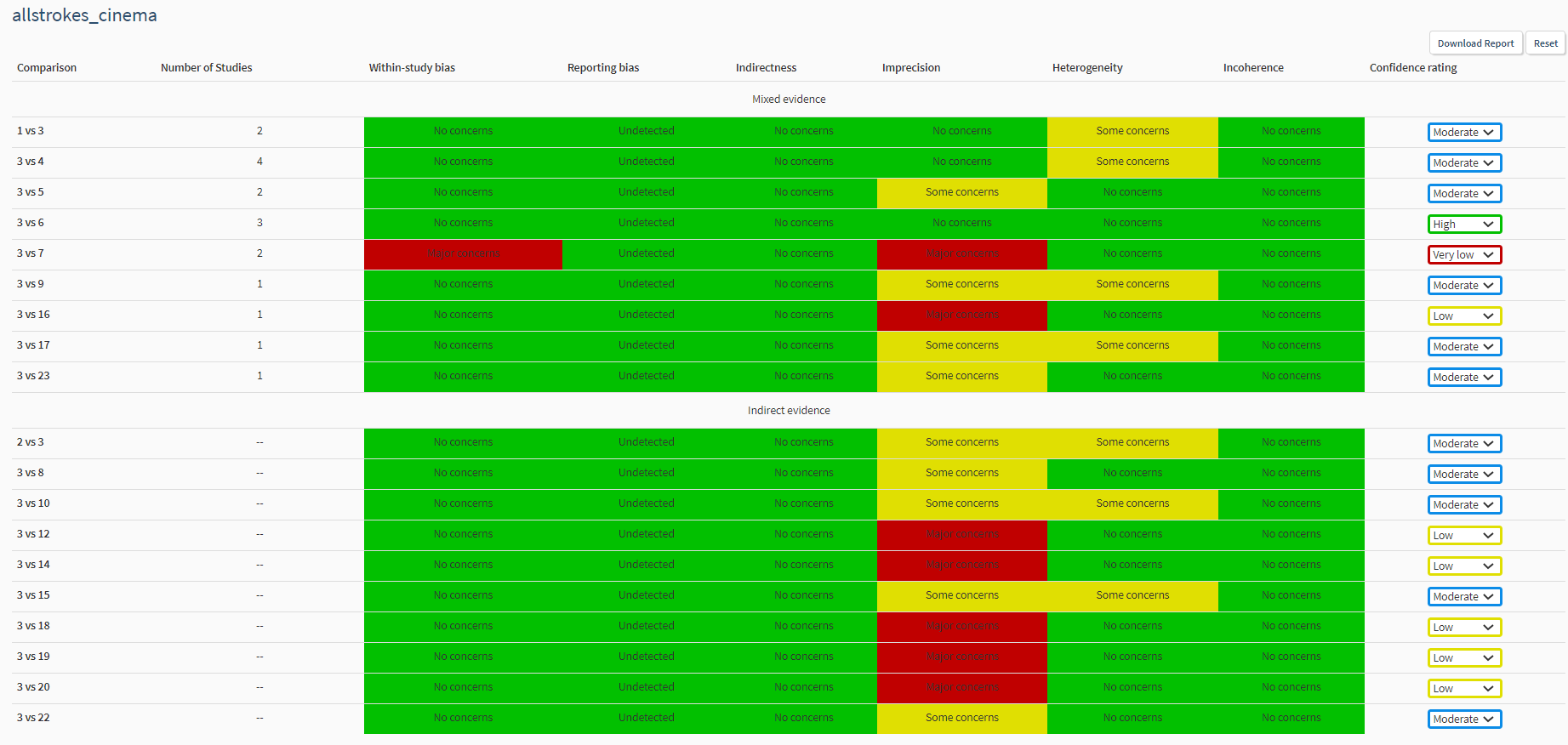
 b) versus aspirin ≤150 mg/day


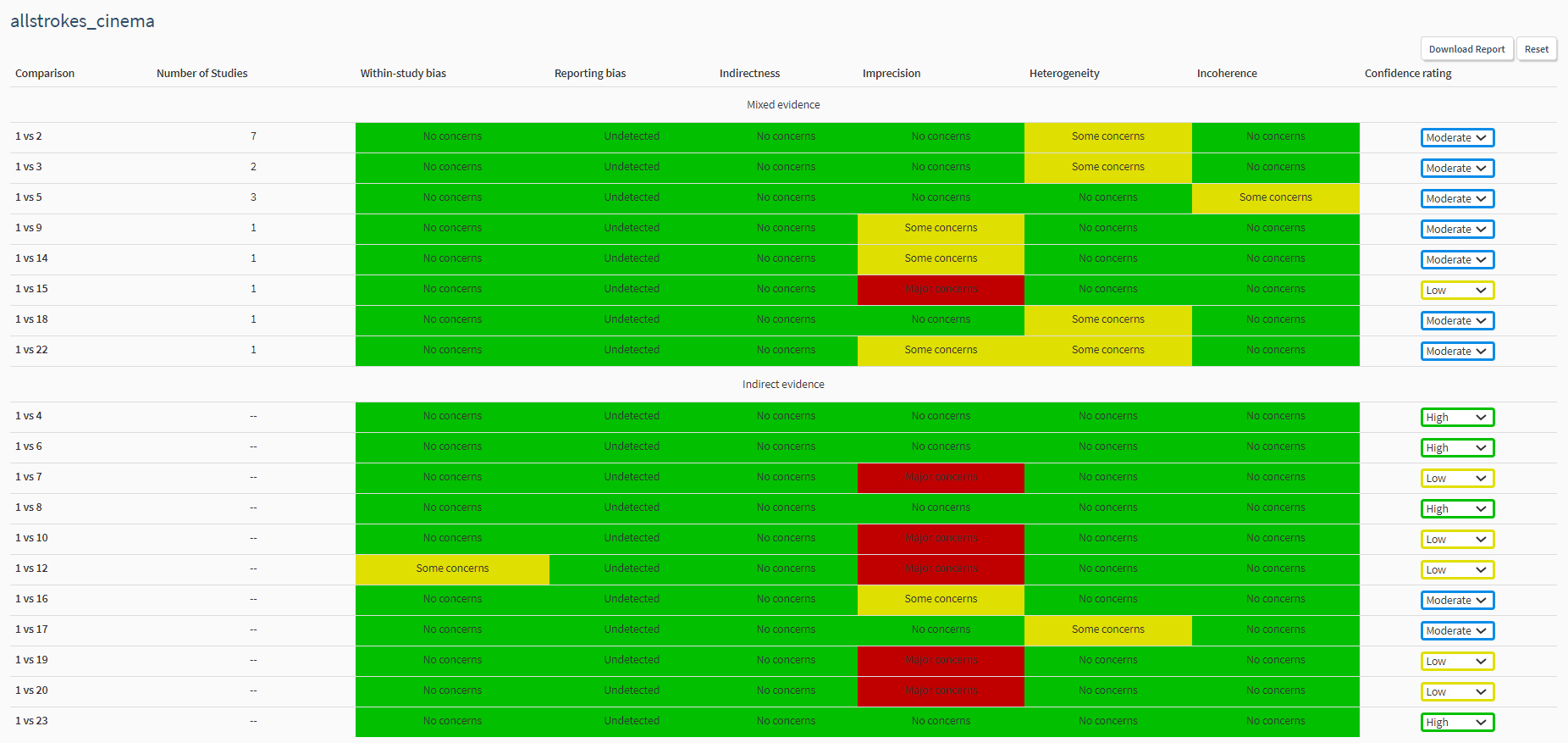
a) versus placebo/no treatment

## *All-cause mortality*

Risk of bias bar chart


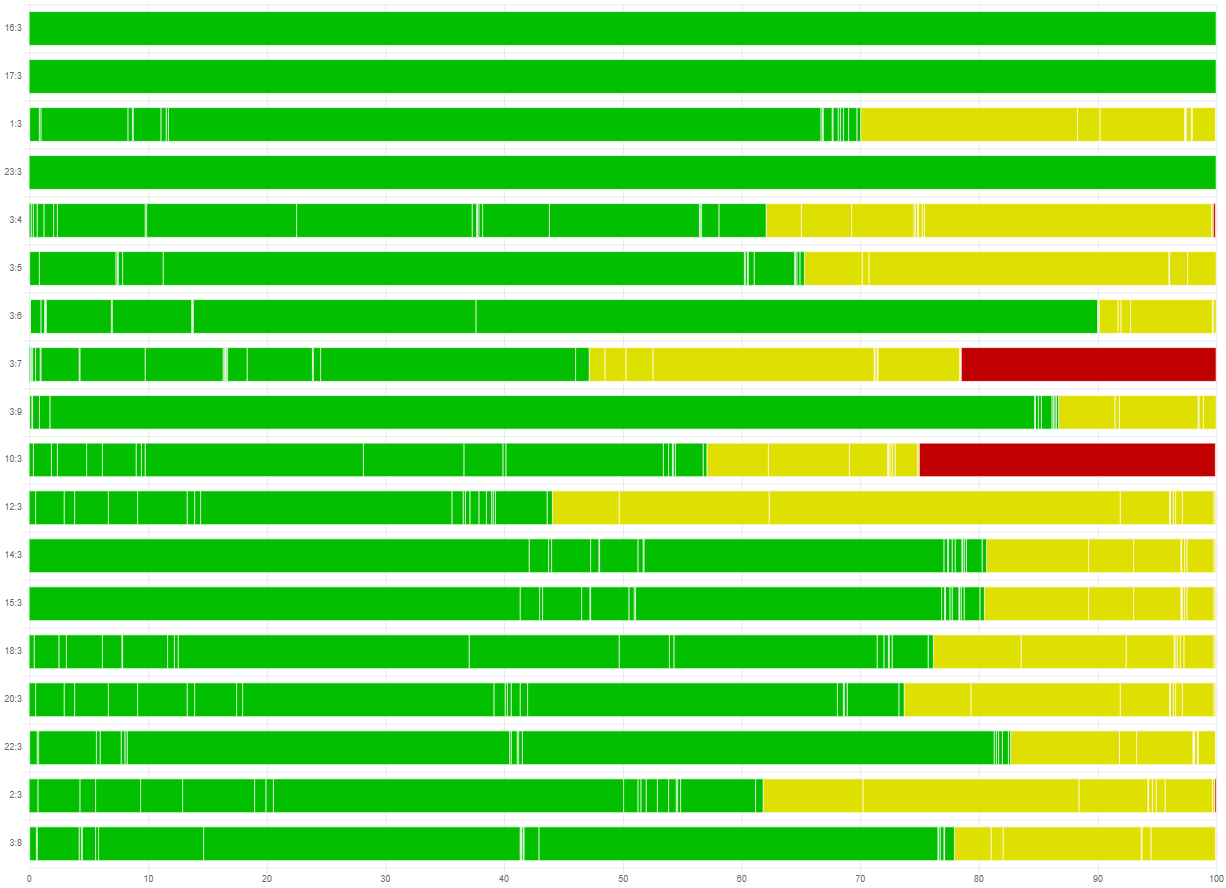


b) versus aspirin ≤150 mg/day


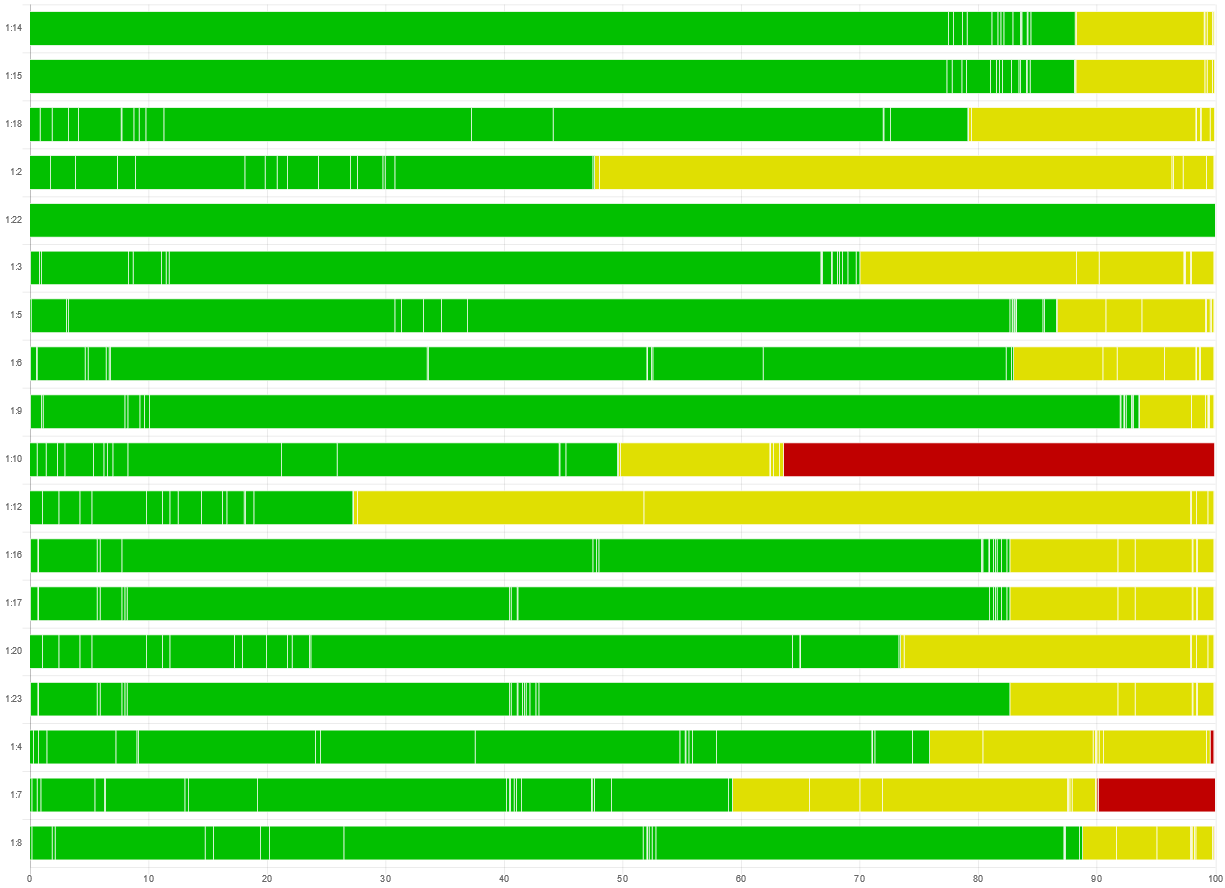


a) versus placebo/no treatment

Forest plot with network estimates and relative prediction intervals


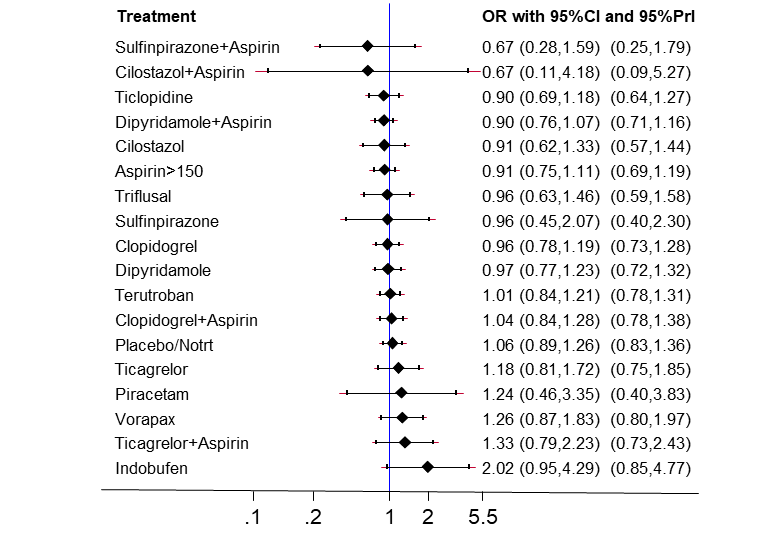


b) versus aspirin ≤150 mg/day


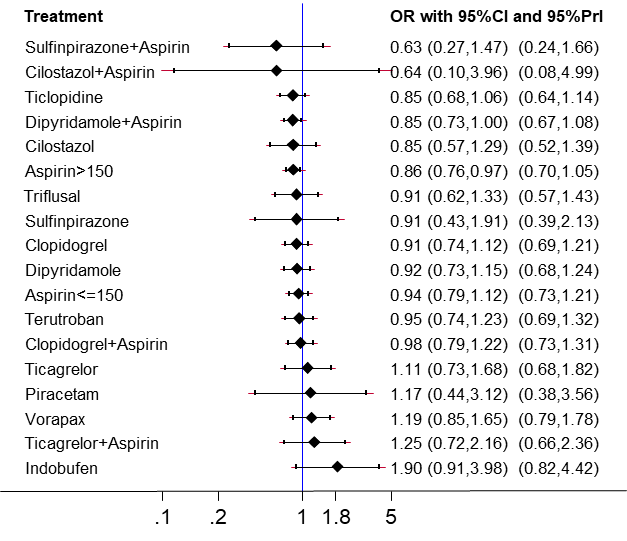


a) versus placebo/no treatment

Rating of the confidence in the network estimates

**
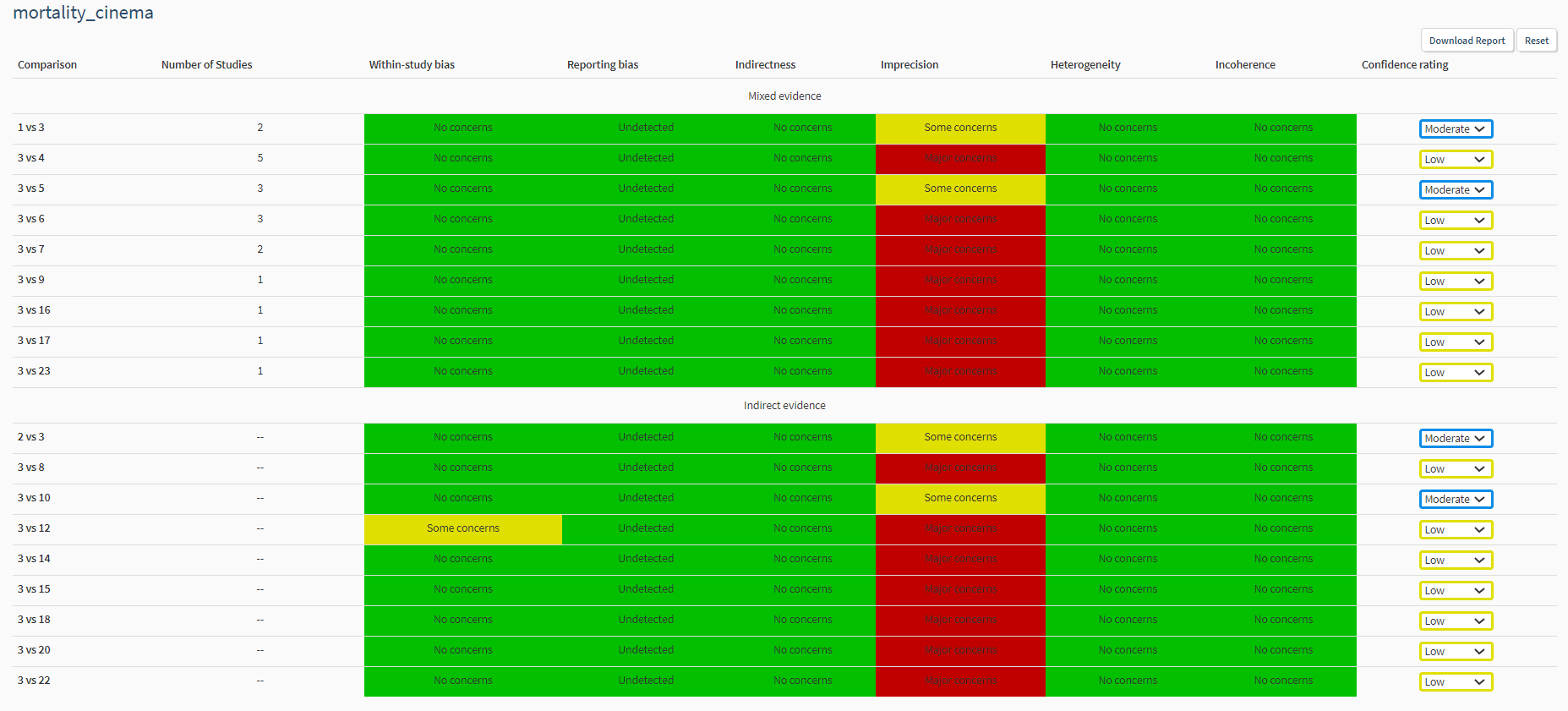
** b) versus aspirin ≤150 mg/day

**
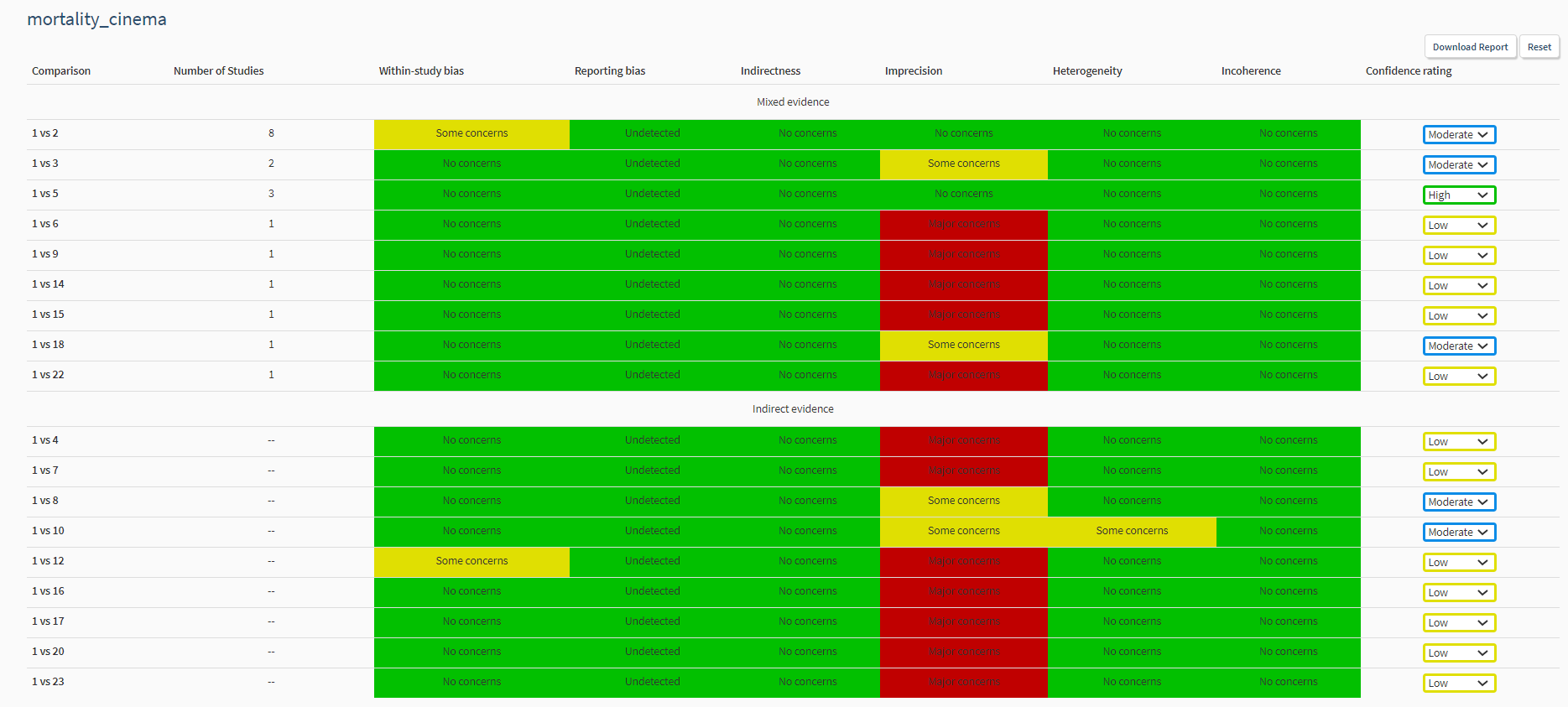
** a) versus placebo/no treatment

## *Ischemic stroke*

Risk of bias bar chart


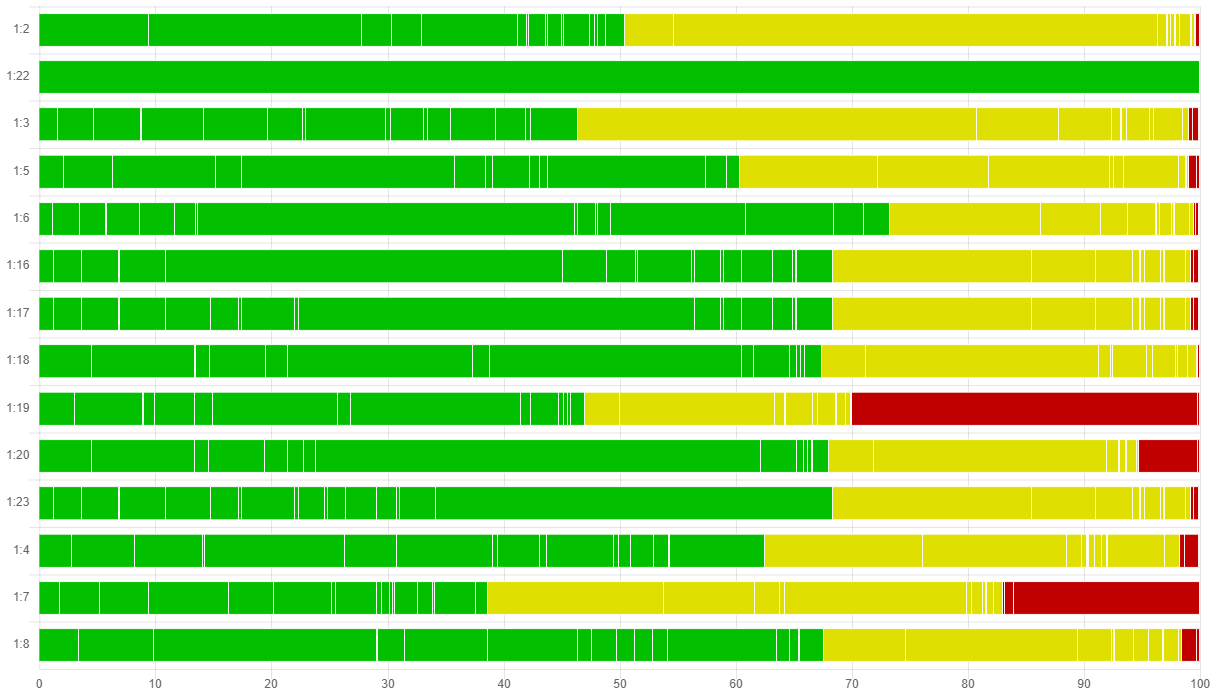


a) versus placebo/no treatment


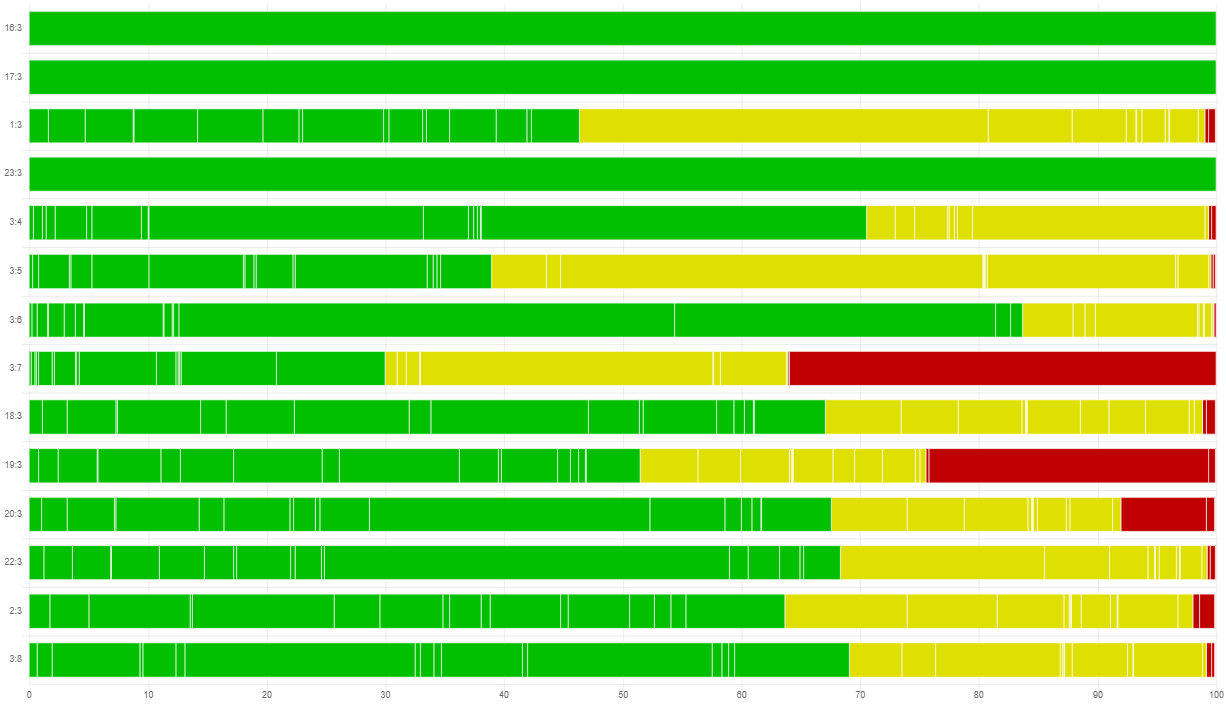


b) versus aspirin ≤150 mg/day

Forest plot with network estimates and relative prediction intervals


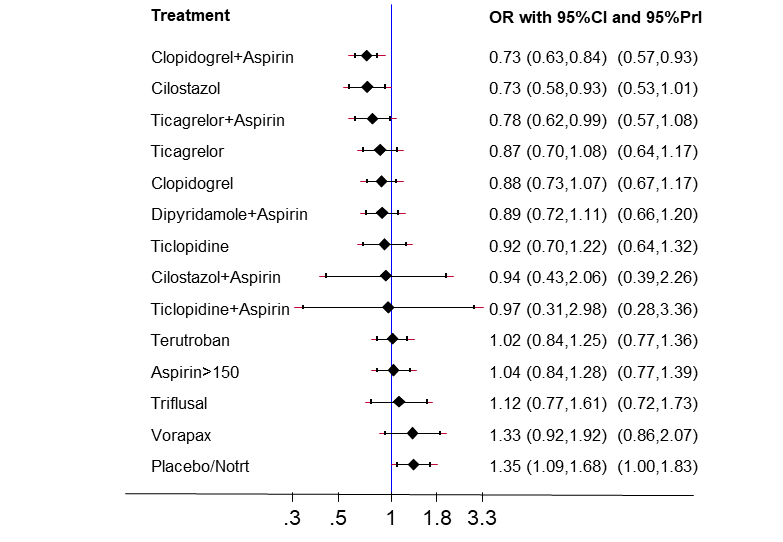


b) versus aspirin ≤150 mg/day


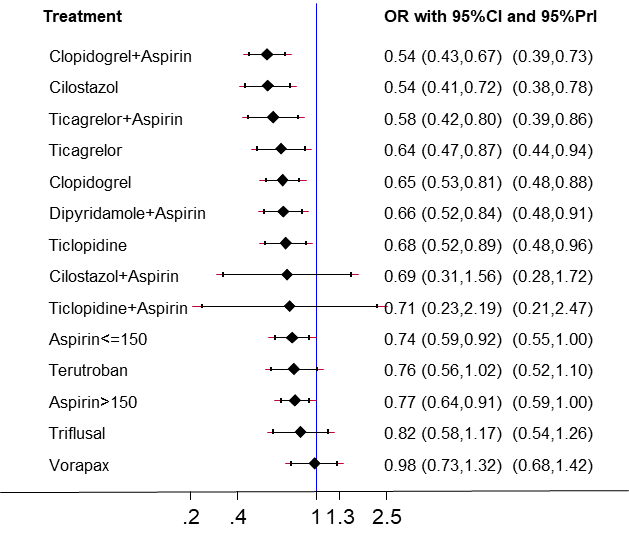


a) versus placebo/no treatment

Rating of the confidence in the network estimates

**
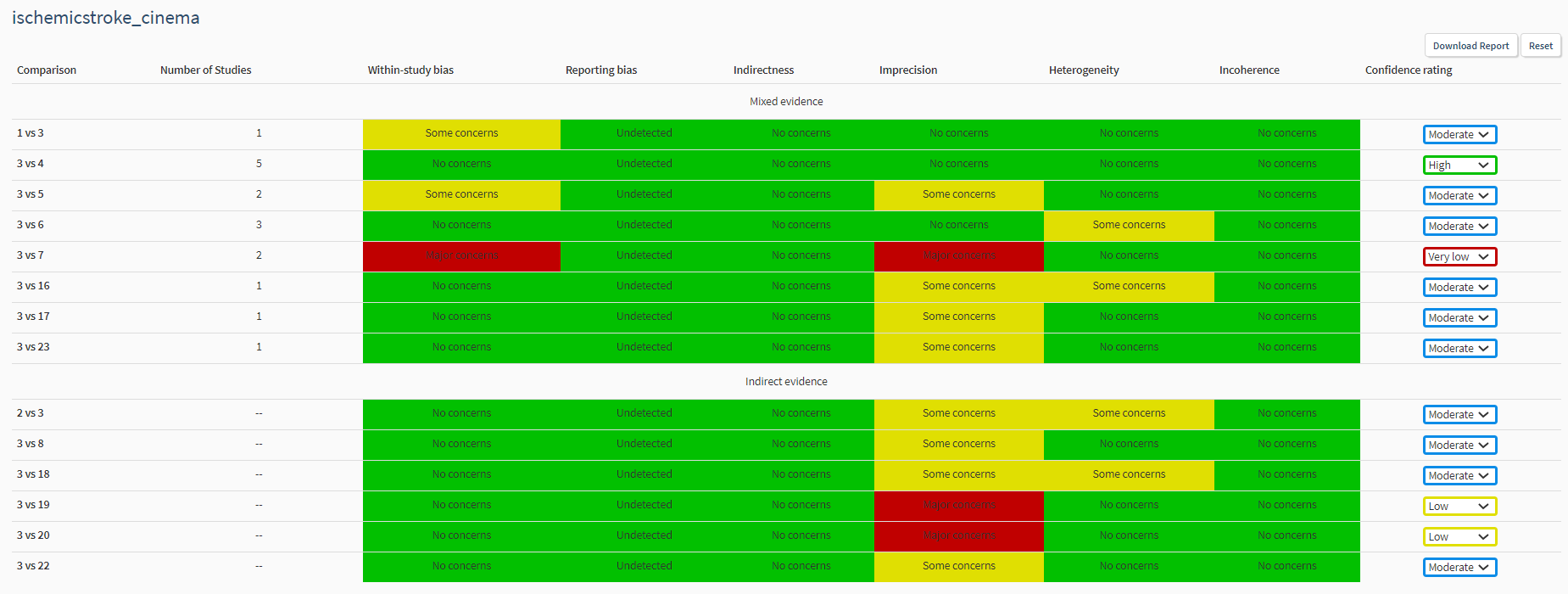
**b) versus aspirin ≤150 mg/day


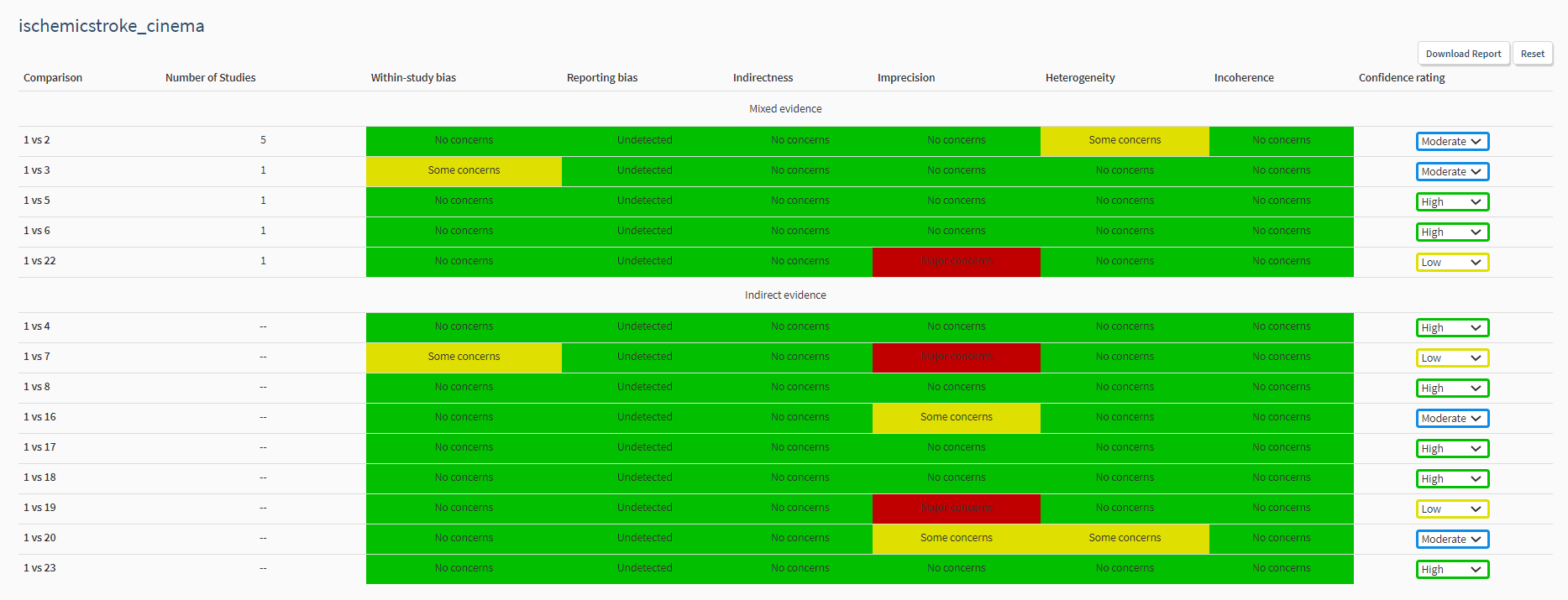
a) versus placebo/no treatment

## *Cardiovascular event*

Risk of bias bar chart


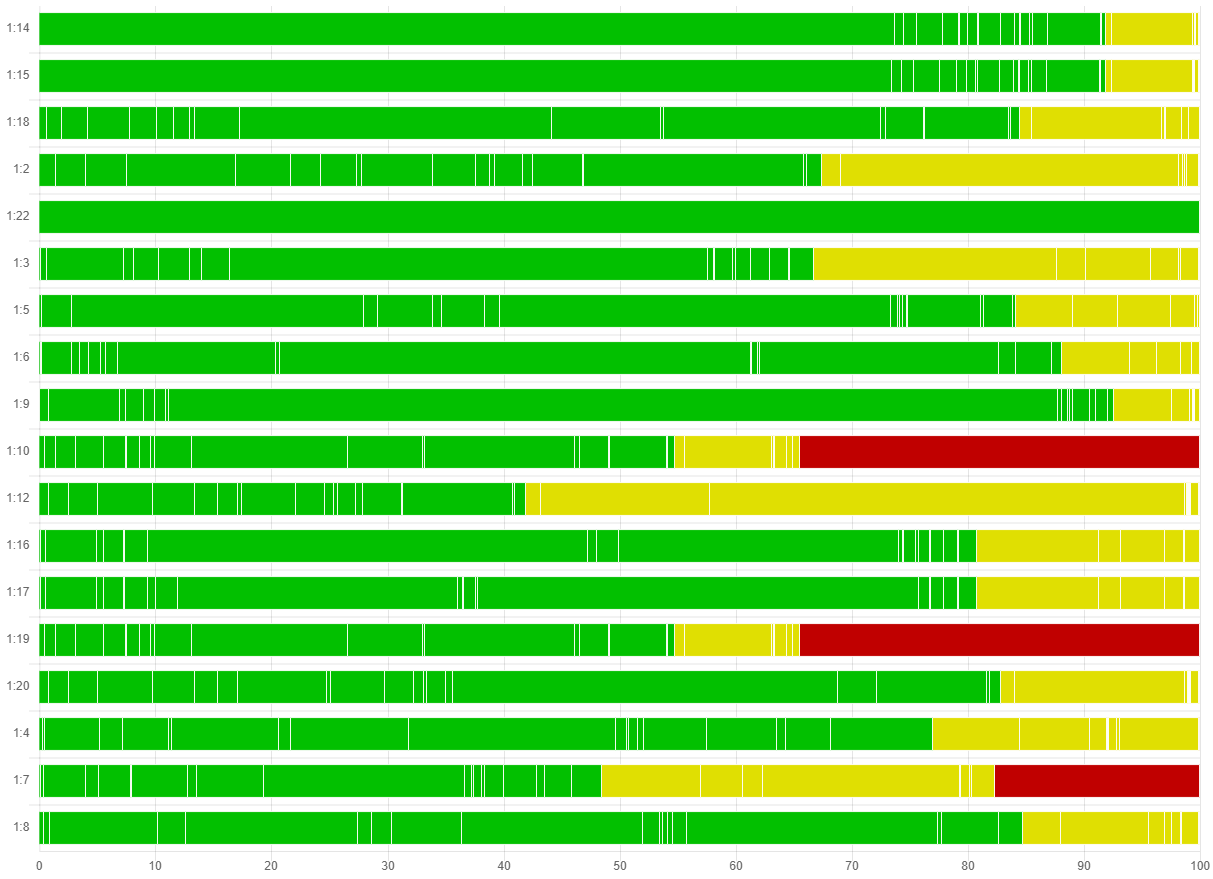
a) versus placebo/no treatment


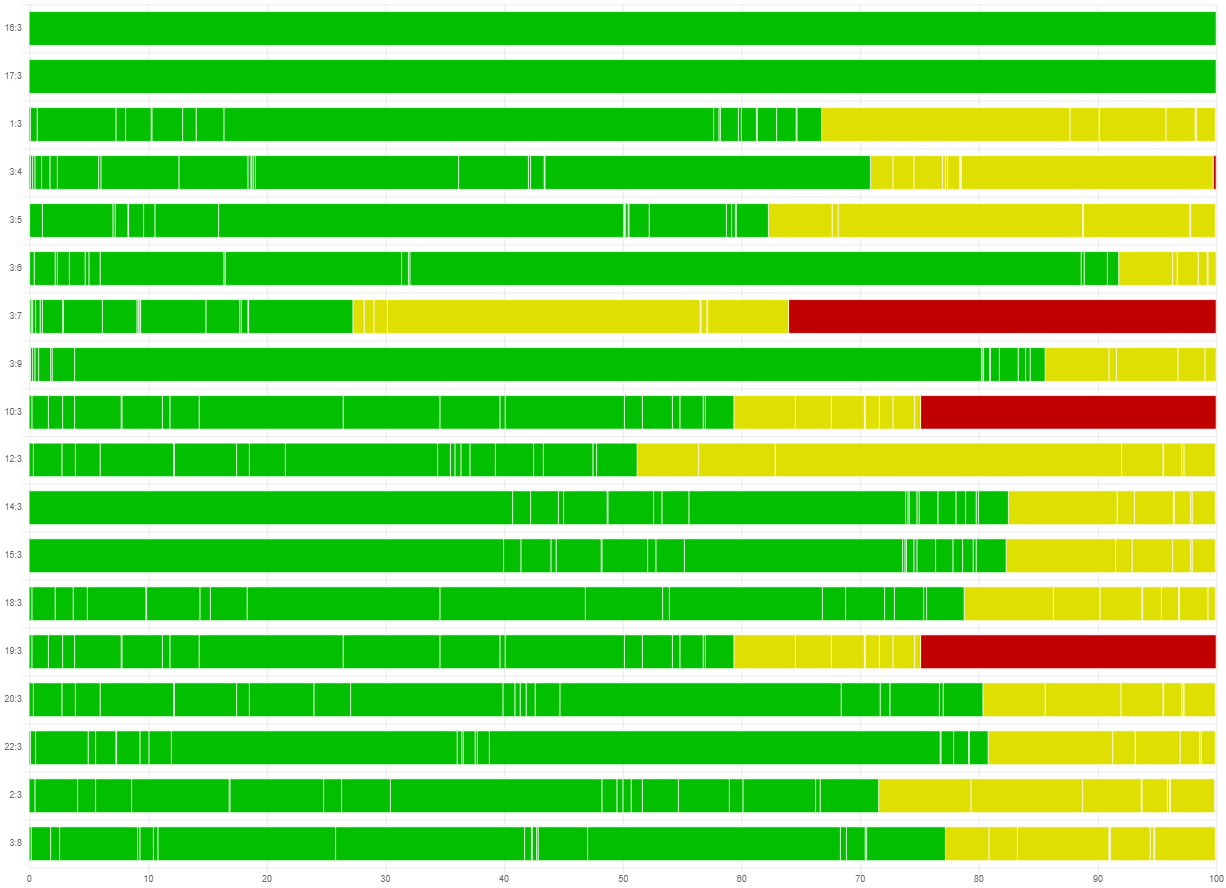
b) versus aspirin ≤150 mg/day

Forest plot with network estimates and relative prediction intervals


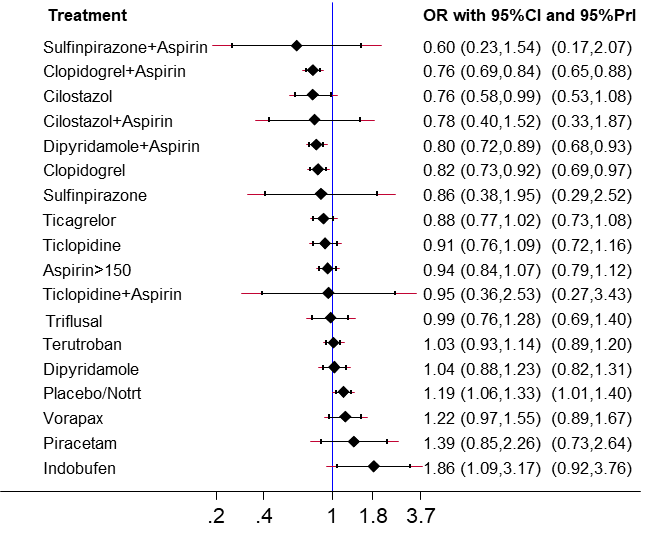


b) versus aspirin ≤150 mg/day


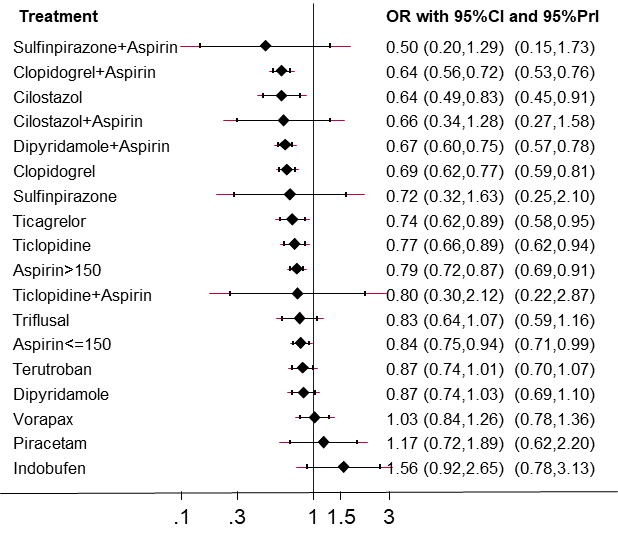


a) versus placebo/no treatment

Rating of the confidence in the network estimates


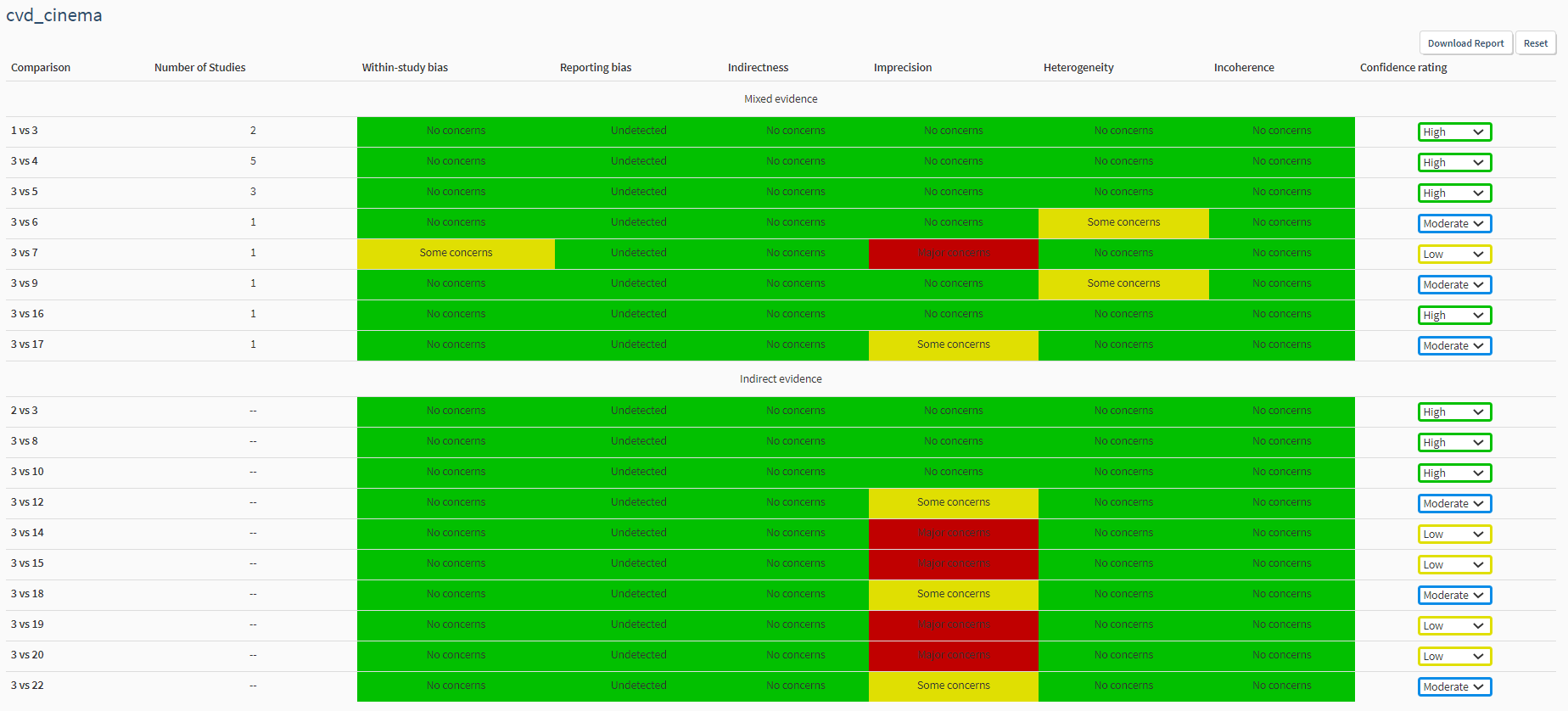


b) versus aspirin ≤150 mg/day


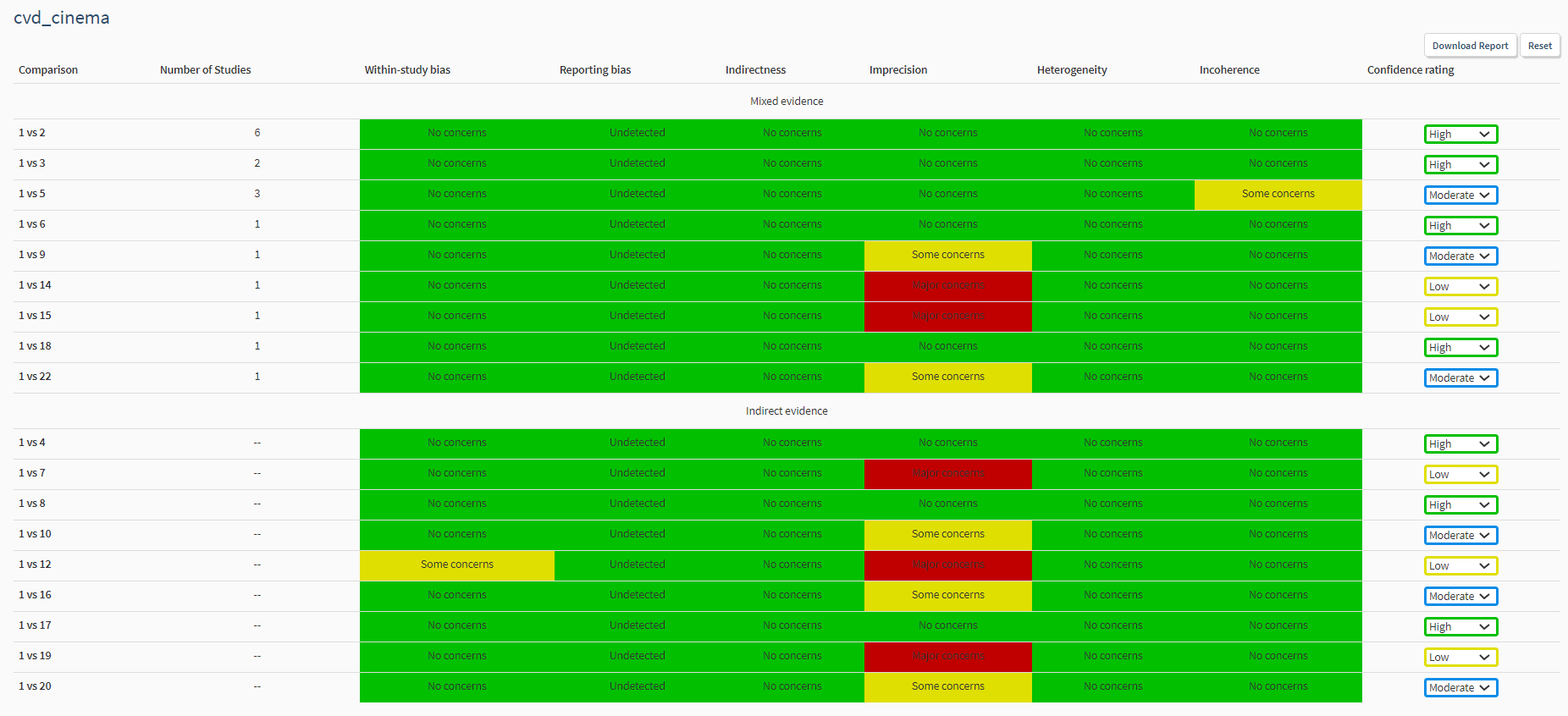


a) versus placebo/no treatment

## *Hemorrhagic stroke*

Risk of bias bar chart


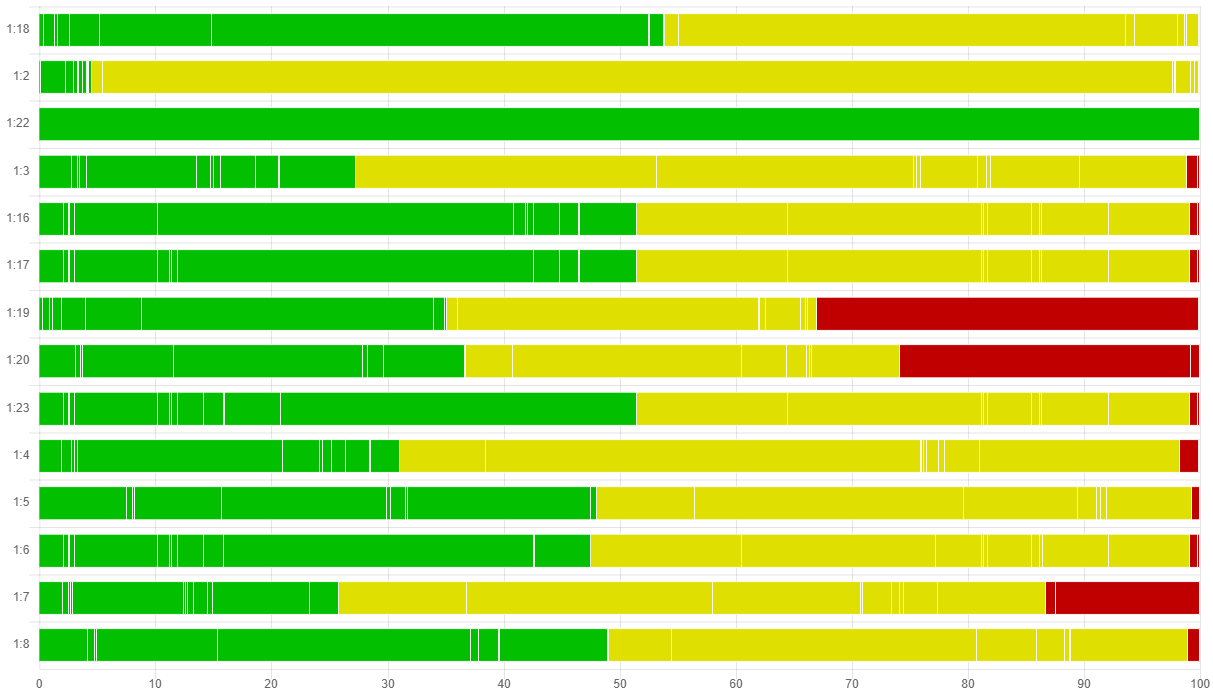


a) versus placebo/no treatment


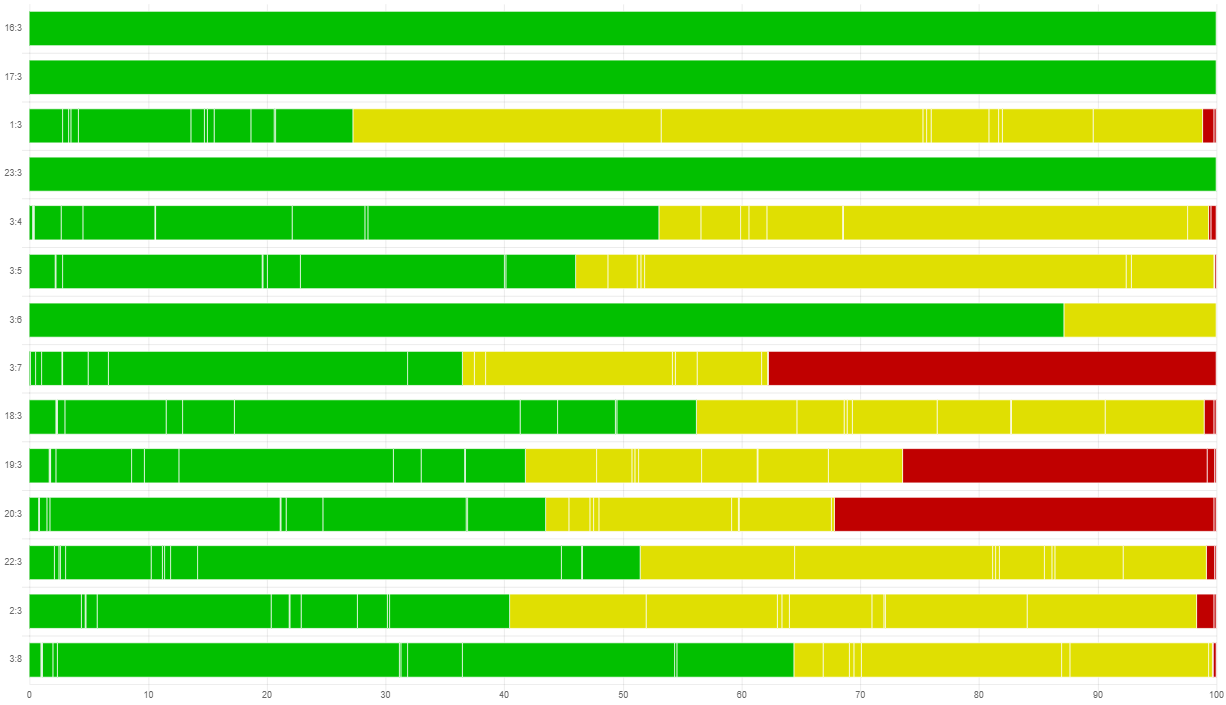


b) versus aspirin ≤150 mg/day

Forest plot with network estimates and relative prediction intervals


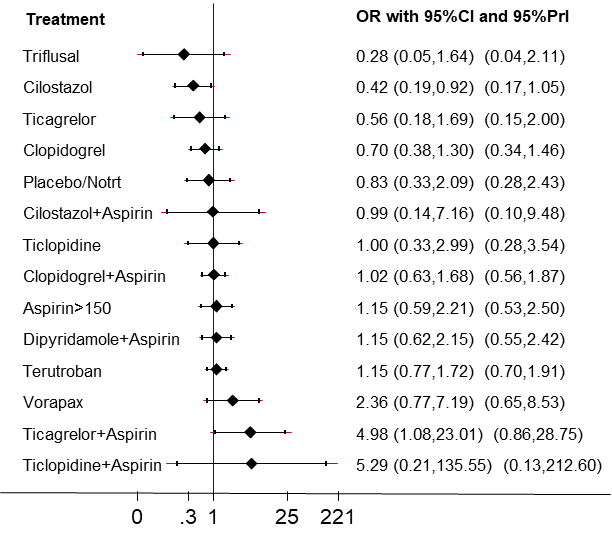


b) versus aspirin ≤150 mg/day


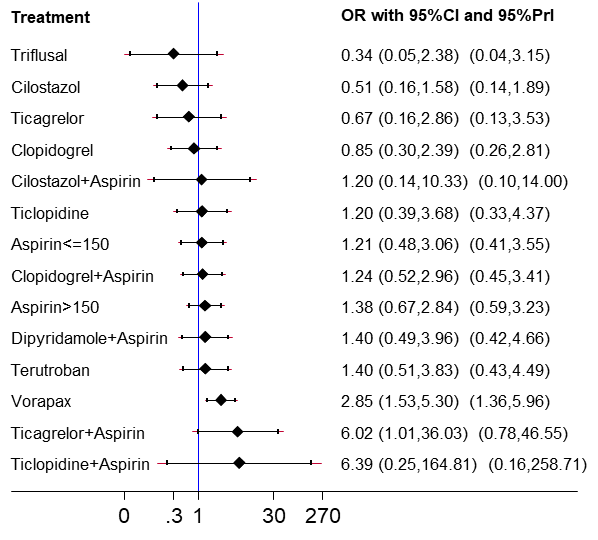


a) versus placebo/no treatment

Rating of the confidence in the network estimates


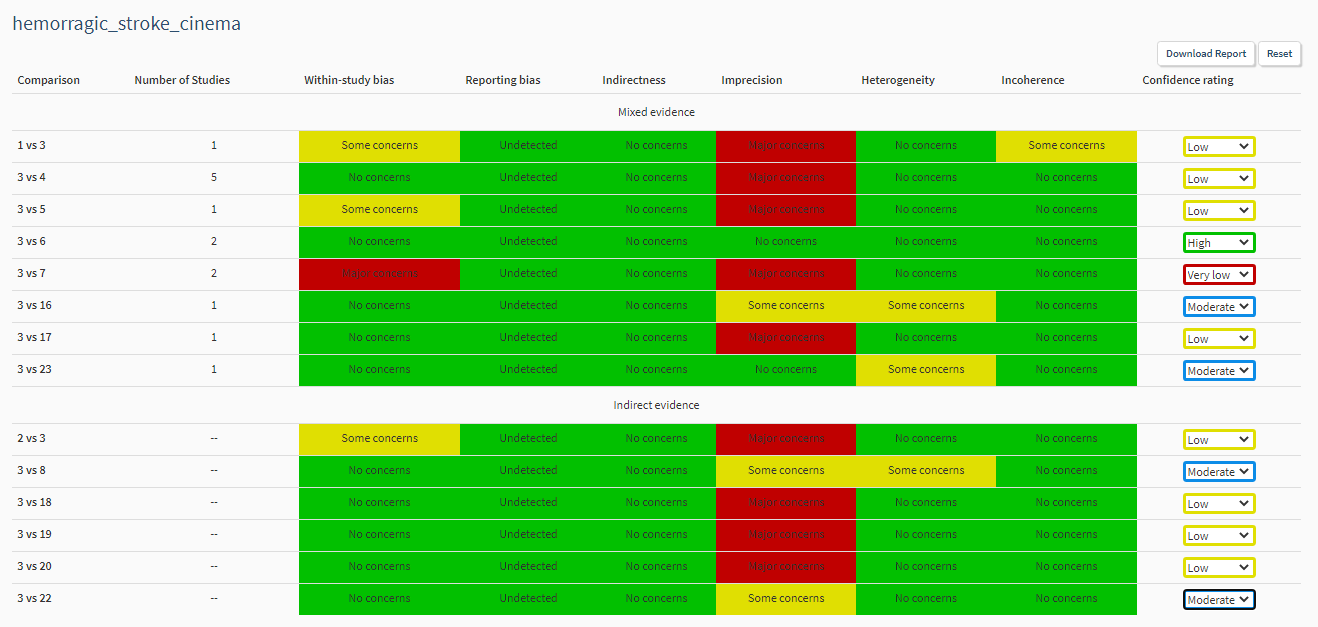


b) versus aspirin ≤150 mg/day


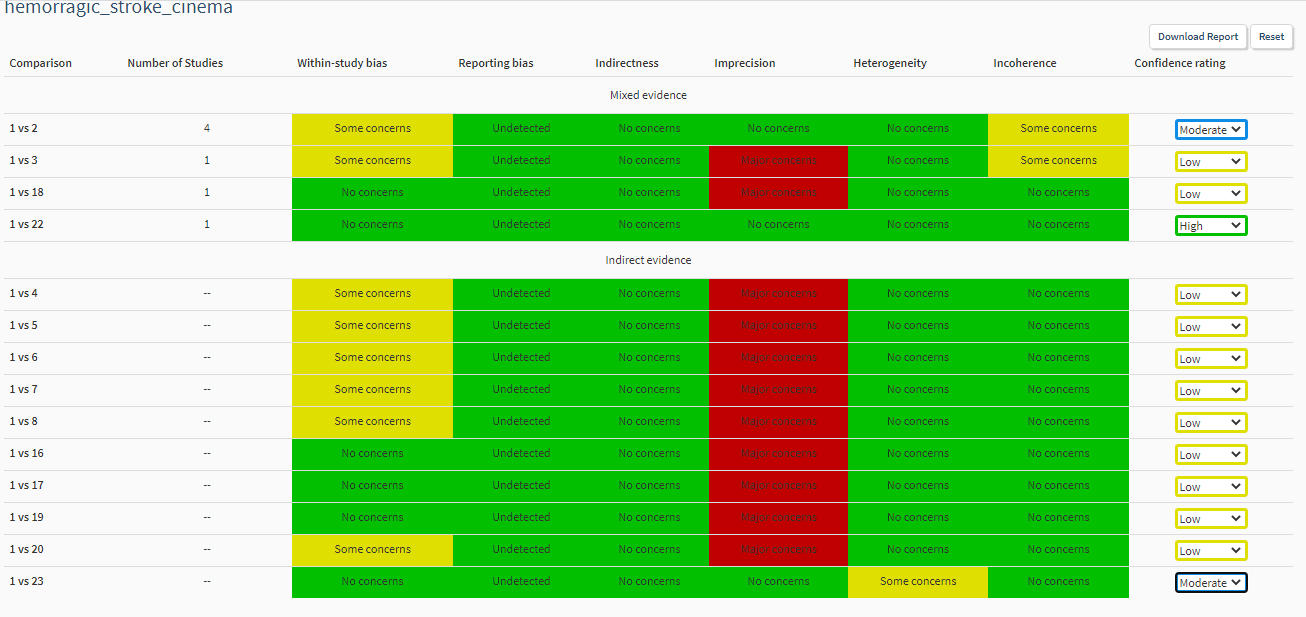


a) versus placebo/no treatment

## *Intracranial haemorrhage*

Risk of bias bar chart


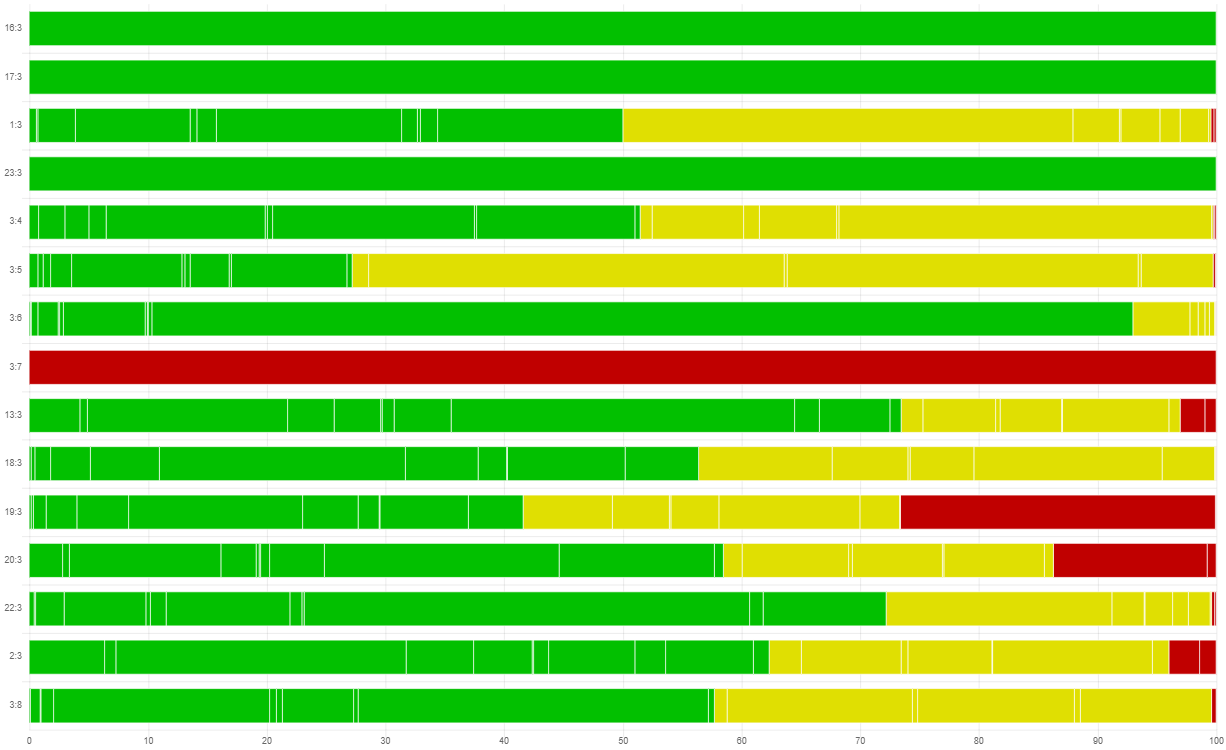


b) versus aspirin ≤150 mg/day


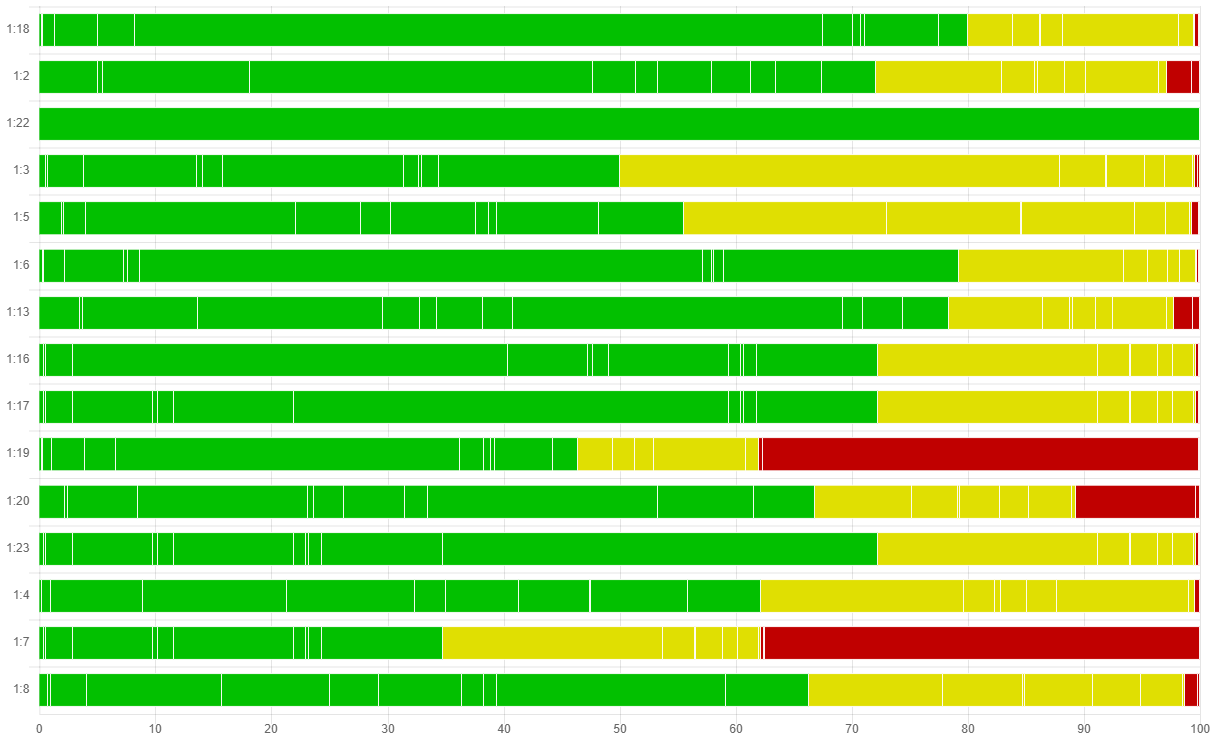


a) versus placebo/no treatment

Forest plot with network estimates and relative prediction intervals


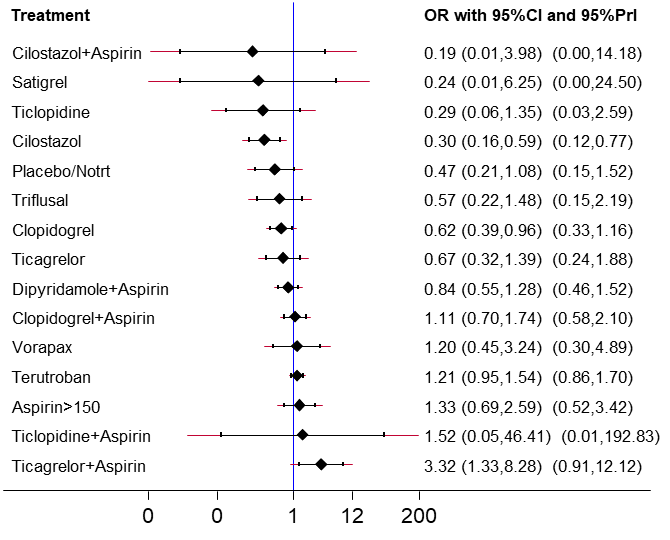
b) versus aspirin ≤150 mg/day


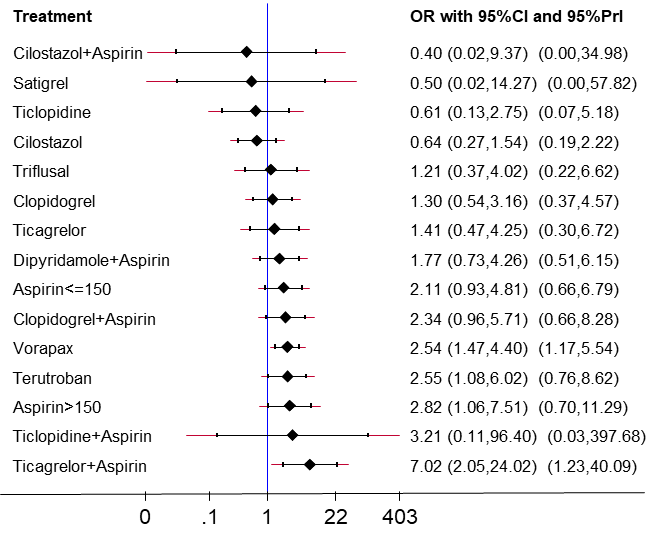


a) versus placebo/no treatment

Rating of the confidence in the network estimates


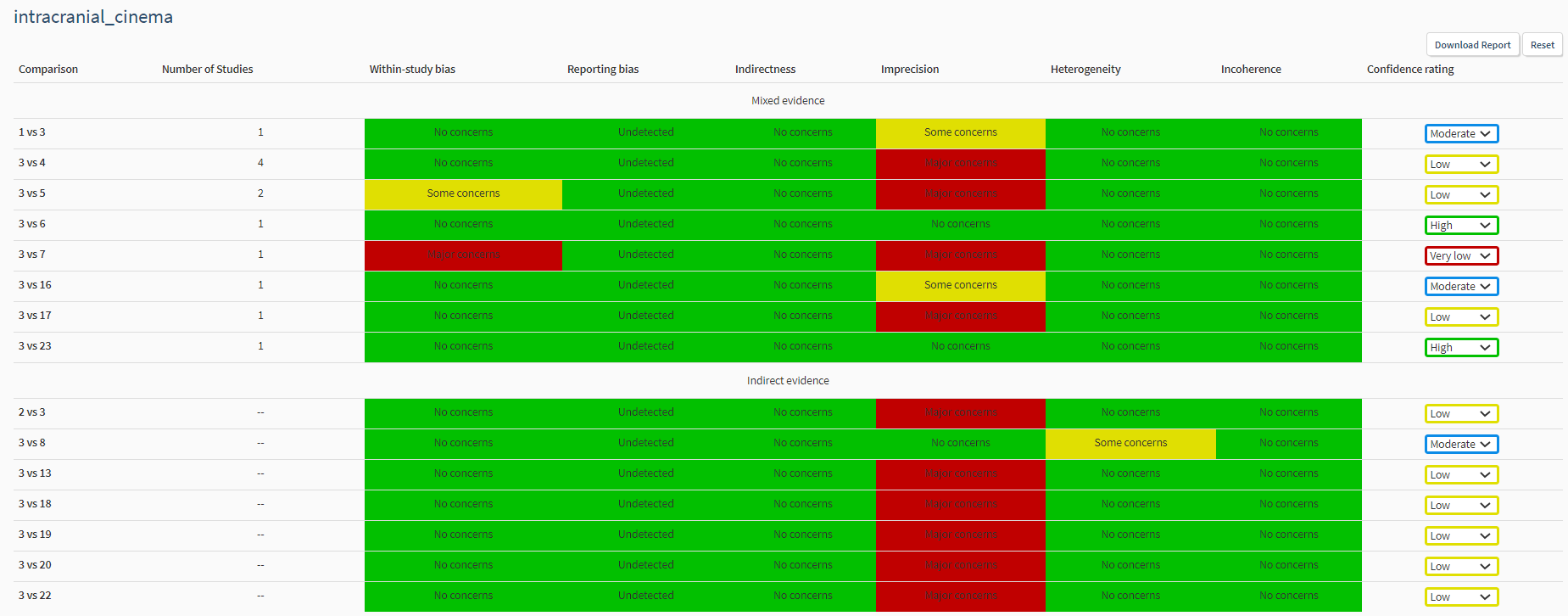


b) versus aspirin ≤150 mg/day


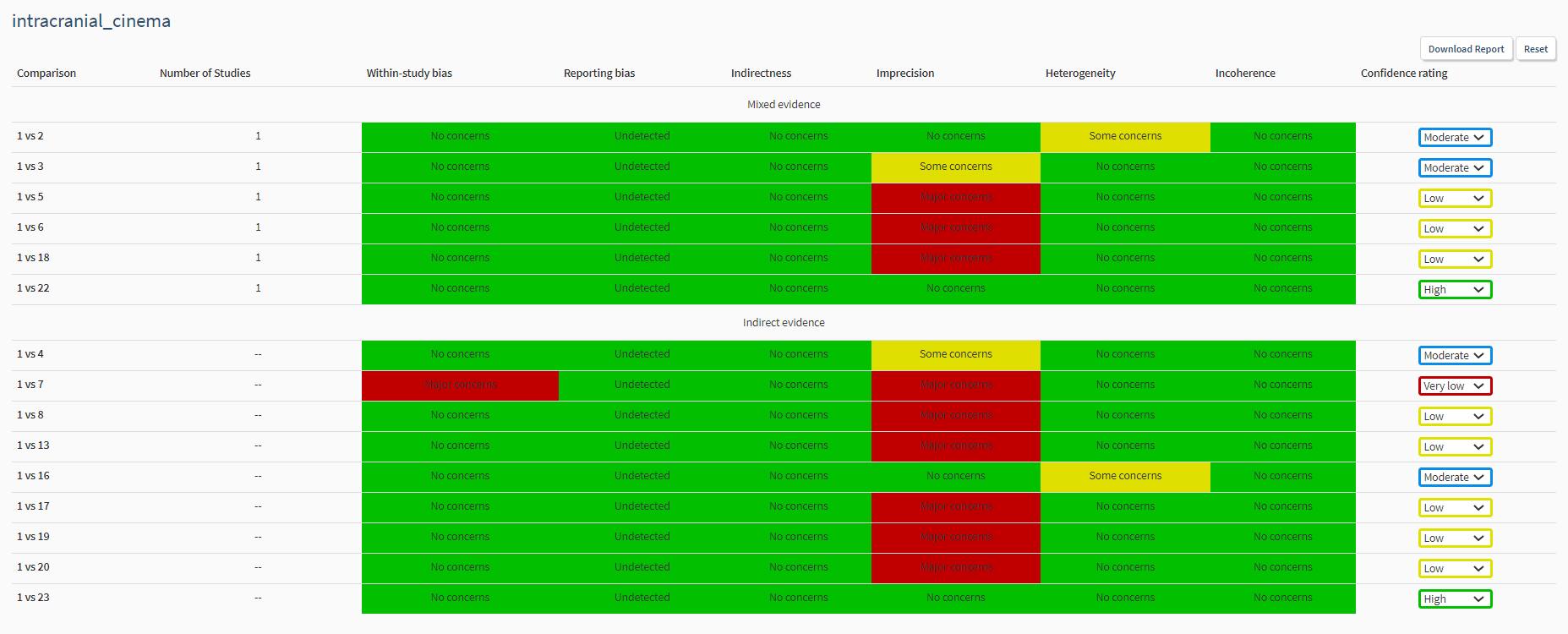


a) versus placebo/no treatment

## *Major bleeding*

Risk of bias bar chart


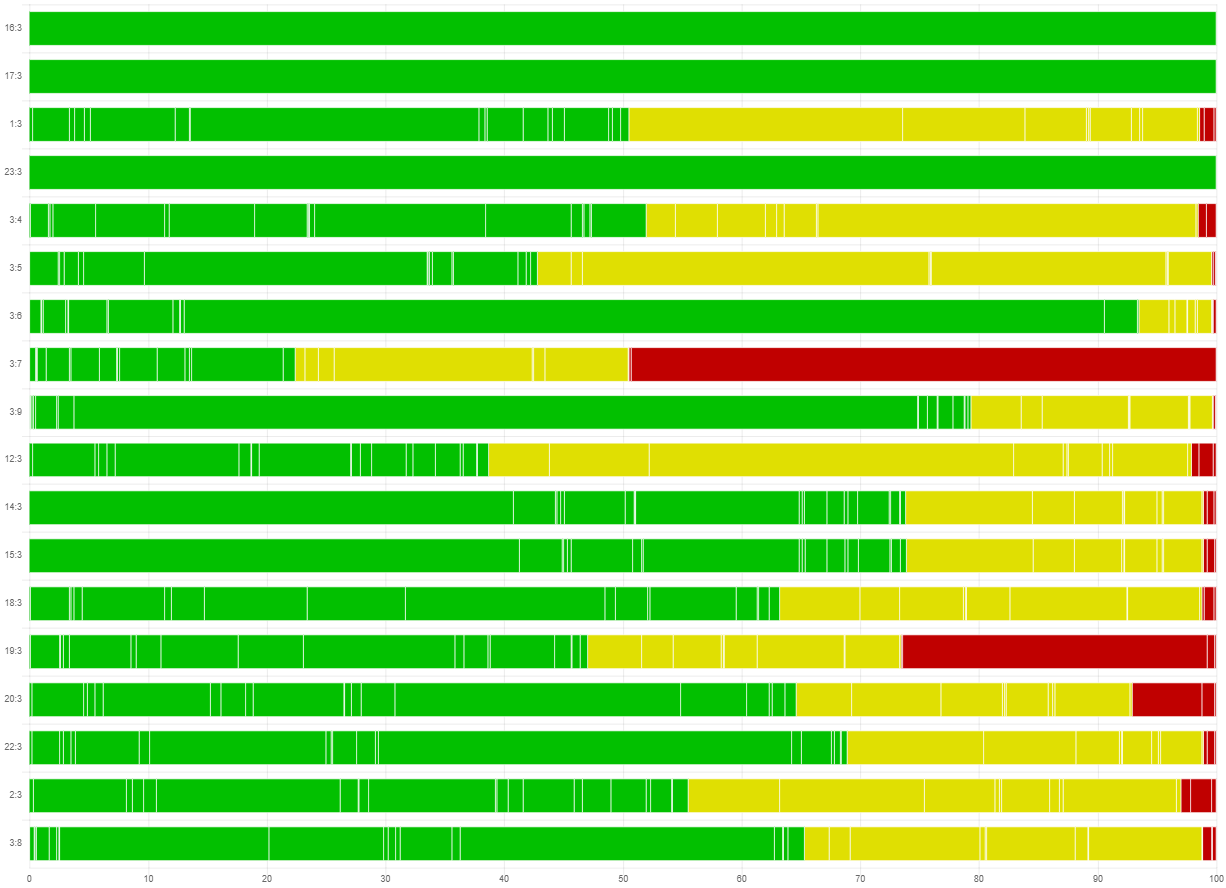


b) versus aspirin ≤150 mg/day


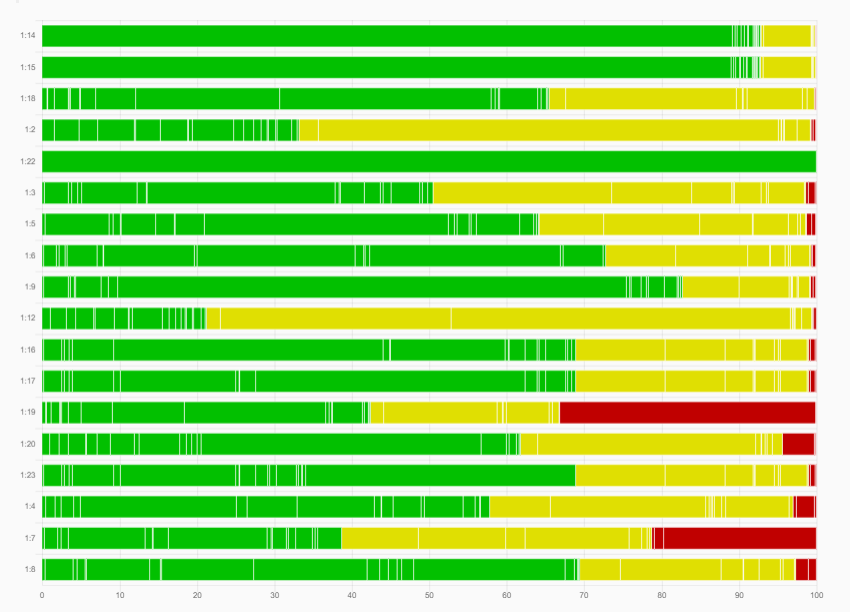


a) versus placebo/no treatment

Forest plot with network estimates and relative prediction intervals


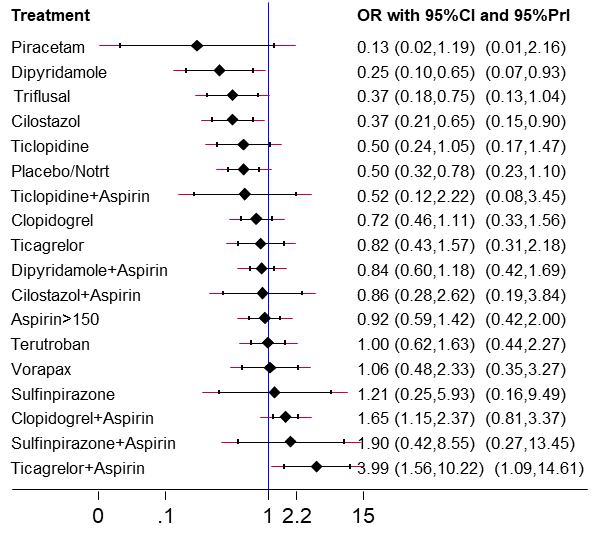


b) versus aspirin ≤150 mg/day


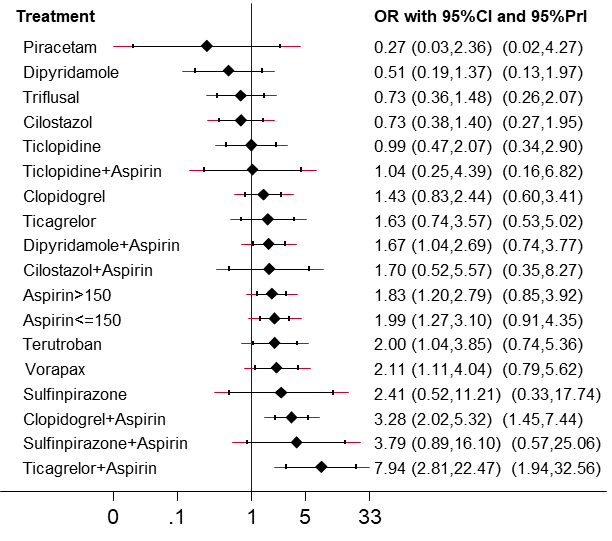


a) versus placebo/no treatment


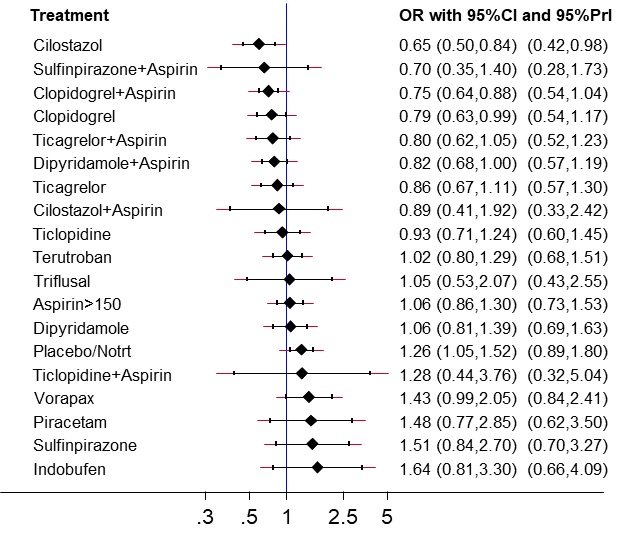


b) versus aspirin ≤150 mg/day

Rating of the confidence in the network estimates

**
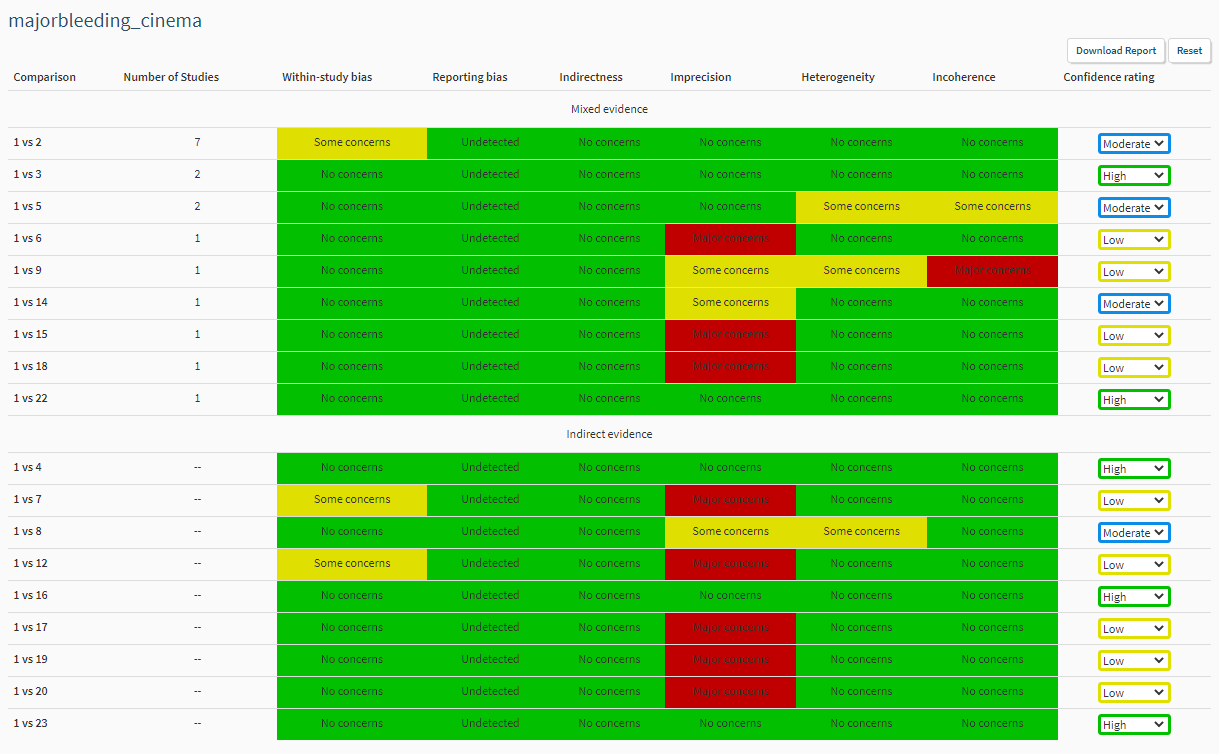
**

a) versus placebo/no treatment

/

**
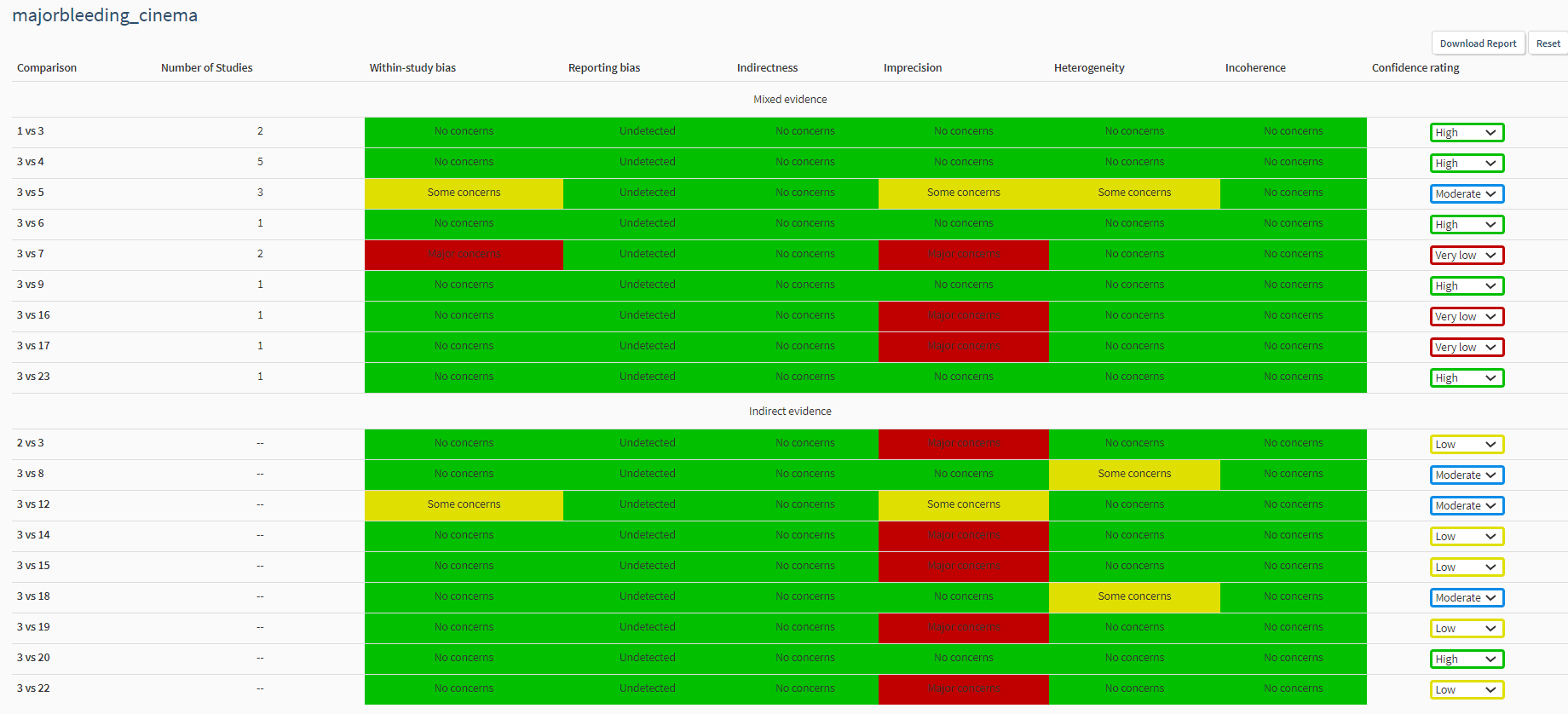
**

b) versus aspirin ≤150 mg/day

# **Supplementary 8. SUCRA, probability to be the best and mean rank by outcome**

Legend: Notrt means No treatment

## *All strokes*

+----------------------------------------------------+

| Treatment | SUCRA | PrBest | MeanRank |

|------------------------+-------+--------+----------|

| Placebo/Notrt | 22.0 | 0.0 | 15.8 |

| Aspirin>150 mg | 40.3 | 0.0 | 12.3 |

| Aspirin<=150 mg | 46.8 | 0.0 | 11.1 |

| Clopidogrel+Aspirin | 83.1 | 2.2 | 4.2 |

| Dipyridamole+Aspirin | 71.9 | 0.4 | 6.3 |

| Cilostazol | 92.2 | 33.3 | 2.5 |

| Cilostazol+Aspirin | 59.3 | 14.0 | 8.7 |

| Clopidogrel | 76.4 | 1.6 | 5.5 |

| Dipyridamole | 40.6 | 0.0 | 12.3 |

| Indobufen | 14.5 | 0.2 | 17.2 |

| Piracetam | 19.4 | 0.3 | 16.3 |

| Sulfinpyrazone+Aspirin | 79.0 | 32.3 | 5.0 |

| Sulfinpyrazone | 16.6 | 0.0 | 16.8 |

| Terutroban | 45.6 | 0.0 | 11.3 |

| Ticagrelor | 65.6 | 1.2 | 7.5 |

| Ticlopidine | 57.1 | 0.2 | 9.1 |

| Ticlopidine+Aspirin | 34.4 | 6.7 | 13.5 |

| Triflusal | 45.5 | 4.2 | 11.4 |

| Vorapaxar | 15.8 | 0.0 | 17.0 |

| Ticagrelor+Aspirin | 73.8 | 3.3 | 6.0 |

+----------------------------------------------------+

## *All-cause mortality*

+----------------------------------------------------+

| Treatment | SUCRA | PrBest | MeanRank |

|------------------------+-------+--------+----------|

| Placebo/Notrt | 38.2 | 0.0 | 12.1 |

| Aspirin>150 mg | 70.1 | 0.4 | 6.4 |

| Aspirin<=150 mg | 50.7 | 0.0 | 9.9 |

| Clopidogrel+Aspirin | 42.9 | 0.0 | 11.3 |

| Dipyridamole+Aspirin | 71.6 | 0.9 | 6.1 |

| Cilostazol | 64.8 | 4.8 | 7.3 |

| Cilostazol+Aspirin | 66.7 | 42.8 | 7.0 |

| Clopidogrel | 57.7 | 0.3 | 8.6 |

| Dipyridamole | 55.8 | 0.5 | 9.0 |

| Indobufen | 6.6 | 0.1 | 17.8 |

| Piracetam | 36.7 | 5.5 | 12.4 |

| Sulfinpyrazone+Aspirin | 80.3 | 32.7 | 4.5 |

| Sulfinpyrazone | 55.3 | 6.5 | 9.1 |

| Terutroban | 49.4 | 0.2 | 10.1 |

| Ticagrelor | 30.6 | 0.3 | 13.5 |

| Ticlopidine | 68.9 | 1.9 | 6.6 |

| Triflusal | 57.7 | 2.5 | 8.6 |

| Vorapaxar | 22.8 | 0.0 | 14.9 |

| Ticagrelor+Aspirin | 23.1 | 0.5 | 14.8 |

+----------------------------------------------------+

## *Ischemic stroke*

+--------------------------------------------------+

| Treatment | SUCRA | PrBest | MeanRank |

|----------------------+-------+--------+----------|

| Placebo/Notrt | 8.0 | 0.0 | 13.9 |

| Aspirin>150 mg | 33.5 | 0.0 | 10.3 |

| Aspirin<=150 mg | 39.1 | 0.0 | 9.5 |

| Clopidogrel+Aspirin | 89.2 | 21.3 | 2.5 |

| Dipyridamole+Aspirin | 59.9 | 0.6 | 6.6 |

| Cilostazol | 85.6 | 22.9 | 3.0 |

| Cilostazol+Aspirin | 51.6 | 17.4 | 7.8 |

| Clopidogrel | 61.9 | 0.1 | 6.3 |

| Terutroban | 36.7 | 0.0 | 9.9 |

| Ticagrelor | 63.9 | 2.1 | 6.1 |

| Ticlopidine | 54.5 | 0.8 | 7.4 |

| Ticlopidine+Aspirin | 49.2 | 24.4 | 8.1 |

| Triflusal | 27.5 | 0.2 | 11.2 |

| Vorapaxar | 11.8 | 0.0 | 13.3 |

| Ticagrelor+Aspirin | 77.6 | 10.1 | 4.1 |

+--------------------------------------------------+

## *Cardiovascular event*

+----------------------------------------------------+

| Treatment | SUCRA | PrBest | MeanRank |

|------------------------+-------+--------+----------|

| Placebo/Notrt | 17.7 | 0.0 | 15.8 |

| Aspirin>150 mg | 50.3 | 0.0 | 10.0 |

| Aspirin<=150 mg | 40.5 | 0.0 | 11.7 |

| Clopidogrel+Aspirin | 84.8 | 4.1 | 3.7 |

| Dipyridamole+Aspirin | 77.6 | 0.6 | 5.0 |

| Cilostazol | 80.1 | 7.5 | 4.6 |

| Cilostazol+Aspirin | 68.5 | 17.1 | 6.7 |

| Clopidogrel | 72.9 | 0.1 | 5.9 |

| Dipyridamole | 34.9 | 0.0 | 12.7 |

| Indobufen | 3.5 | 0.0 | 18.4 |

| Piracetam | 14.1 | 0.0 | 16.5 |

| Sulfinpyrazone+Aspirin | 81.5 | 46.7 | 4.3 |

| Sulfinpyrazone | 58.5 | 10.7 | 8.5 |

| Terutroban | 35.5 | 0.0 | 12.6 |

| Ticagrelor | 61.5 | 0.2 | 7.9 |

| Ticlopidine | 56.5 | 0.0 | 8.8 |

| Ticlopidine+Aspirin | 50.0 | 12.8 | 10.0 |

| Triflusal | 44.1 | 0.2 | 11.1 |

| Vorapaxar | 17.4 | 0.0 | 15.9 |

+----------------------------------------------------+

## *Hemorrhagic stroke*

+--------------------------------------------------+

| Treatment | SUCRA | PrBest | MeanRank |

|----------------------+-------+--------+----------|

| Placebo/Notrt | 61.6 | 1.5 | 6.4 |

| Aspirin>150mg/day | 41.6 | 0.0 | 9.2 |

| Aspirin<=150mg/day | 50.5 | 0.0 | 7.9 |

| Clopidogrel+Aspirin | 48.5 | 0.0 | 8.2 |

| Dipyridamole+Aspirin | 40.0 | 0.0 | 9.4 |

| Cilostazol | 87.2 | 19.6 | 2.8 |

| Cilostazol+Aspirin | 50.9 | 8.5 | 7.9 |

| Clopidogrel | 72.4 | 0.8 | 4.9 |

| Terutroban | 40.0 | 0.0 | 9.4 |

| Ticagrelor | 75.2 | 11.3 | 4.5 |

| Ticlopidine | 51.2 | 1.0 | 7.8 |

| Ticlopidine+Aspirin | 17.8 | 3.0 | 12.5 |

| Triflusal | 88.9 | 54.3 | 2.6 |

| Vorapaxar | 16.5 | 0.0 | 12.7 |

| Ticagrelor+Aspirin | 7.7 | 0.0 | 13.9 |

+--------------------------------------------------+

## *Intracranial haemorrhage*

+--------------------------------------------------+

| Treatment | SUCRA | PrBest | MeanRank |

|----------------------+-------+--------+----------|

| Placebo/Notrt | 71.4 | 0.8 | 5.3 |

| Aspirin>150 mg | 21.4 | 0.0 | 12.8 |

| Aspirin<=150 mg | 37.1 | 0.0 | 10.4 |

| Clopidogrel+Aspirin | 29.9 | 0.0 | 11.5 |

| Dipyridamole+Aspirin | 47.1 | 0.0 | 8.9 |

| Cilostazol | 84.8 | 7.4 | 3.3 |

| Cilostazol+Aspirin | 78.8 | 38.5 | 4.2 |

| Clopidogrel | 63.7 | 0.0 | 6.4 |

| Satigrel | 73.5 | 33.0 | 5.0 |

| Terutroban | 24.1 | 0.0 | 12.4 |

| Ticagrelor | 57.1 | 0.3 | 7.4 |

| Ticlopidine | 80.7 | 13.7 | 3.9 |

| Ticlopidine+Aspirin | 33.8 | 5.3 | 10.9 |

| Triflusal | 63.4 | 0.9 | 6.5 |

| Vorapaxar | 29.3 | 0.0 | 11.6 |

| Ticagrelor+Aspirin | 4.1 | 0.0 | 15.4 |

+--------------------------------------------------+

## *Major bleeding*

+----------------------------------------------------+

| Treatment | SUCRA | PrBest | MeanRank |

|------------------------+-------+--------+----------|

| Placebo/Notrt | 72.2 | 0.1 | 6.0 |

| Aspirin>150 mg | 38.7 | 0.0 | 12.0 |

| Aspirin<=150 mg | 33.1 | 0.0 | 13.0 |

| Clopidogrel+Aspirin | 12.9 | 0.0 | 16.7 |

| Dipyridamole+Aspirin | 44.9 | 0.0 | 10.9 |

| Cilostazol | 82.9 | 3.3 | 4.1 |

| Cilostazol+Aspirin | 44.5 | 0.5 | 11.0 |

| Clopidogrel | 54.7 | 0.0 | 9.2 |

| Dipyridamole | 90.0 | 20.9 | 2.8 |

| Piracetam | 91.0 | 64.5 | 2.6 |

| Sulfinpyrazone+Aspirin | 17.7 | 0.1 | 15.8 |

| Sulfinpyrazone | 32.7 | 0.7 | 13.1 |

| Terutroban | 34.8 | 0.0 | 12.7 |

| Ticagrelor | 46.4 | 0.0 | 10.7 |

| Ticlopidine | 71.0 | 0.6 | 6.2 |

| Ticlopidine+Aspirin | 64.6 | 5.5 | 7.4 |

| Triflusal | 82.5 | 3.7 | 4.2 |

| Vorapaxar | 33.1 | 0.0 | 13.0 |

| Ticagrelor+Aspirin | 2.4 | 0.0 | 18.6 |

+----------------------------------------------------+

# **Supplementary 9.** **Results from the assessment of incoherence by using global and local (side-split method) approaches for primary and secondary outcomes**

## *All strokes*

Design-by-treatment interaction test

P-value = 0.092

Side-splitting model

Legend: 01=Placebo / No treatment; 02=Aspirin>150 mg; 03=Aspirin≤150 mg; 04=Clopidogrel 75 mg + Aspirin; 05=Dipyridamole + Aspirin; 06=Cilostazol 200 mg; 07=Cilostazol 200 mg + Aspirin; 08=Clopidogrel 75 mg; 09=Dipyridamole>200 mg; 10=Indobufen 200 mg; 11=Piracetam 4800 mg; 12=Sulfinpyrazone 800 mg + Aspirin>150 mg; 13=Sulfinpyrazone 800 mg; 14=Terutroban 30 mg; 15=Ticagrelor 180 mg; 16=Ticlopidine≤500 mg; 17=Ticlopidine<500 mg + Aspirin≤150 mg; 18=Triflusal 600 mg; 19=Vorapaxar 2.5 mg; 20=Ticagrelor 180 mg + Aspirin≤150 mg.

Side Direct Indirect Difference

Coef. Std. Err. Coef. Std. Err. Coef. Std. Err. P>|z|

01 02 * -.0921363 .0625563 -.4071492 .1128243 .315013 .129003 0.015

01 03 * -.2180151 .1255995 -.2763202 .1702102 .0583051 .2117996 0.783

01 05 * -.5450663 .1005869 -.1869755 .1499086 -.3580908 .1806864 0.047

01 09 * -.2005694 .1576387 -.071763 .3025859 -.1288064 .3390768 0.704

01 12 * -.4603534 .3871015 -.9756916 .6405918 .5153382 .720419 0.474

01 13 * .3065 .3364517 -.2088396 .6113179 .5153395 .7204183 0.474

01 16 -.5772492 .2127662 -.1882003 .1387728 -.3890489 .2540223 0.126

01 19 . . . . . . .

02 04 -.2275626 .1614962 -.42615 .1326122 .1985874 .2072954 0.338

02 05 -.118018 .1940644 -.2978568 .1165204 .1798388 .2262472 0.427

02 11 * .3334973 .3188411 .3607595 224.264 -.0272622 224.2643 1.000

02 12 * -.530766 .3809137 -.0154266 .6516239 -.5153394 .7204185 0.474

02 13 * .2360873 .329314 .7514267 .6228692 -.5153394 .7204185 0.474

02 16 -.0428815 .1188571 -.4319272 .2244421 .3890457 .2540217 0.126

03 04 -.3244795 .091806 -.1796177 .1580942 -.1448619 .183217 0.429

03 05 * -.0931052 .1422014 -.3009019 .1468593 .2077967 .2021447 0.304

03 06 * -.4370749 .1333687 .4230187 145.5078 -.8600936 145.5078 0.995

03 07 -.648031 .5860313 .3190335 .5335728 -.9670645 .7925336 0.222

03 09 * .023982 .1651709 .2348672 .3382324 -.2108852 .376713 0.576

03 14 * .0169963 .1203154 .4675506 223.6691 -.4505542 223.6692 0.998

03 15 * -.1494121 .130234 .4626862 256.4915 -.6120983 256.4915 0.998

03 20 * -.2182615 .1365251 .4579383 263.6327 -.6761997 263.6327 0.998

04 07 .5970457 .5276411 -.3700086 .5913593 .9670543 .7925348 0.222

04 08 .0241925 .1531486 .1143089 .1981539 -.0901164 .2504385 0.719

05 08 -.0185503 .1395187 -.1086622 .2079757 .0901119 .2504383 0.719

05 09 * .3296755 .1635501 -.0052864 .3095323 .3349619 .3546444 0.345

08 18 * .2840453 .3251663 .9204079 269.1141 -.6363626 269.1144 0.998

10 16 * -.561827 .3281459 -.5346913 382.964 -.0271357 382.9642 1.000

12 13 . . . . . . .

16 17 * .3143274 .5306438 .5897171 267.4167 -.2753897 267.4173 0.999

* Warning: all the evidence about these contrasts comes from the trials which directly compare them.

## *All-cause mortality*

Design-by-treatment interaction test

P-value = 0.292

Side-splitting model

Legend: 01=Placebo / No treatment; 02=Aspirin>150 mg; 03=Aspirin≤150 mg; 04=Clopidogrel 75 mg + Aspirin; 05=Dipyridamole + Aspirin; 06=Cilostazol 200 mg; 07=Cilostazol 200 mg + Aspirin; 08=Clopidogrel 75 mg; 09=Dipyridamole>200 mg; 10=Indobufen 200 mg; 11=Piracetam 4800 mg; 12=Sulfinpyrazone 800 mg + Aspirin>150 mg; 13=Sulfinpyrazone 800 mg; 14=Terutroban 30 mg; 15=Ticagrelor 180 mg; 16=Ticlopidine≤500 mg; 17=Triflusal 600 mg; 18=Vorapaxar 2.5 mg; 19=Ticagrelor 180 mg + Aspirin≤150 mg.

Side Direct Indirect Difference

Coef. Std. Err. Coef. Std. Err. Coef. Std. Err. P>|z|

01 02 * -.1161833 .0678946 -.3163801 .136127 .2001968 .1513381 0.186

01 03 * -.1183699 .1126886 .0531124 .1523753 -.1714823 .1895761 0.366

01 05 * -.2207263 .0917148 -.0227688 .1375948 -.1979574 .1645622 0.229

01 06 -.1053605 .4693224 -.1698778 .2351047 .0645173 .5249169 0.902

01 09 * -.0848709 .140107 -.0821507 .2818809 -.0027202 .3136358 0.993

01 12 * -.5764796 .4715681 -.001134 .85011 -.5753456 .9221117 0.533

01 13 * -.2135741 .4247449 .3617716 .8250574 -.5753457 .9221126 0.533

01 16 .0064865 .1993831 -.2329817 .1295788 .2394682 .2377904 0.314

01 18 . . . . . . .

02 04 .3818267 .1490769 -.059802 .1224723 .4416286 .1929336 0.022

02 05 .1022769 .2179272 -.0301633 .1036281 .1324402 .2405976 0.582

02 11 * .3088383 .4984912 .3143986 369.8705 -.0055603 369.8709 1.000

02 12 * -.1397619 .5059998 -.7151077 .7884868 .5753457 .9221125 0.533

02 13 * .2231436 .462674 -.3522022 .7614086 .5753457 .9221126 0.533

02 16 -.0620295 .1140647 .1774393 .2088134 -.2394687 .2377898 0.314

02 17 * .0556169 .186377 .3737049 202.9274 -.318088 202.9275 0.999

03 04 -.0767518 .163088 .132455 .1475797 -.2092068 .2159169 0.333

03 05 * -.0725341 .1058091 -.1719824 .1568414 .0994483 .1888134 0.598

03 06 -.10938 .2171561 -.044862 .4779515 -.0645181 .5249162 0.902

03 07 -.0035682 1.418978 -.6914323 1.234995 .6878642 1.881146 0.715

03 09 * .0331172 .1328895 -.2879156 .279379 .3210327 .3099388 0.300

03 14 * .0112965 .0927339 .1210501 261.4816 -.1097536 261.4817 1.000

03 15 * .1638073 .1929832 .0598392 606.8868 .1039681 606.8869 1.000

03 19 * .2838474 .2648948 .1061178 849.8326 .1777296 849.8327 1.000

04 07 -.7283931 1.230367 -.040524 1.422988 -.6878691 1.881142 0.715

04 08 -.0013894 .126655 -.2031516 .1670371 .2017623 .2096256 0.336

05 08 .027732 .0917804 .2294965 .1884648 -.2017645 .209625 0.336

05 09 * .0154037 .133467 .2988909 .2593457 -.2834872 .2919711 0.332

10 16 * -.8033051 .3595749 -.1910754 418.1288 -.6122296 418.1291 0.999

12 13 . . . . . . .

* Warning: all the evidence about these contrasts comes from the trials which directly compare them.

## *Ischemic stroke*

Design-by-treatment interaction test

P-value = 0.200

Side-splitting model

Legend: 01=Placebo / No treatment; 02=Aspirin>150 mg; 03=Aspirin≤150 mg; 04=Clopidogrel 75 mg + Aspirin; 05=Dipyridamole + Aspirin; 06=Cilostazol 200 mg; 07=Cilostazol 200 mg + Aspirin; 08=Clopidogrel 75 mg; 09=Terutroban 30 mg; 10=Ticagrelor 180 mg; 11=Ticlopidine≤500 mg; 12=Ticlopidine<500 mg + Aspirin≤150 mg; 13=Triflusal 600 mg; 14=Vorapaxar 2.5 mg; 15=Ticagrelor 180 mg + Aspirin≤150 mg.

Side Direct Indirect Difference

Coef. Std. Err. Coef. Std. Err. Coef. Std. Err. P>|z|

01 02 -.2597601 .1041697 -.2804537 .2038709 .0206935 .2298211 0.928

01 03 -.2728179 .1880737 -.3302225 .1474457 .0574046 .2389811 0.810

01 05 -.6391881 .3259181 -.3763484 .1386183 -.2628397 .3531218 0.457

01 06 -.6949276 .2506579 -.5744286 .1792318 -.120499 .3081451 0.696

01 14 . . . . . . .

02 04 -.4357921 .1700334 -.3244258 .1314287 -.1113663 .2038538 0.585

02 05 .034502 .3652623 -.1678587 .1154181 .2023607 .3833177 0.598

02 06 -.6238285 .572117 -.3272235 .1522567 -.2966051 .5920302 0.616

02 08 -.0905502 .1265614 -.2276562 .1226931 .137106 .1762707 0.437

02 11 -.0891407 .1174928 -.2511997 .2578664 .162059 .2843112 0.569

02 13 .0077514 .1706353 .3572181 .3584897 -.3494668 .3970279 0.379

03 04 -.3527755 .0811147 -.2092234 .1537362 -.1435521 .1747887 0.411

03 05 -.0298126 .1444159 -.1974605 .1519032 .1676479 .2055648 0.415

03 06 -.2692866 .1362139 -.4493037 .2549289 .1800171 .2895315 0.534

03 07 -.3798606 .6069566 .1819127 .5372389 -.5617733 .8105717 0.488

03 09 * .0240006 .1007741 .6280236 199.9111 -.604023 199.9111 0.998

03 10 * -.1414591 .1119138 .4632533 202.8916 -.6047123 202.8916 0.998

03 15 * -.2421917 .1193445 .3627783 209.7211 -.60497 209.7211 0.998

04 07 .4972076 .5320823 -.0645346 .6114987 .5617421 .8105783 0.488

04 08 .0802632 .1120311 .3248976 .126354 -.2446344 .1688677 0.147

05 08 .0276811 .1101255 -.1074248 .1699233 .1351059 .2024884 0.505

08 11 -.0719412 .2384874 .0901289 .1545176 -.1620701 .2843132 0.569

08 13 .4995326 .3478706 .1500712 .1913567 .3494615 .397028 0.379

11 12 * .0468836 .5555938 .8125416 200.1181 -.765658 200.1174 0.997

* Warning: all the evidence about these contrasts comes from the trials which directly compare them.

## *Cardiovascular event*

Design-by-treatment interaction test

P-value = 0.424

Side-splitting model

Legend: 01=Placebo / No treatment; 02=Aspirin>150 mg; 03=Aspirin≤150 mg; 04=Clopidogrel 75 mg + Aspirin; 05=Dipyridamole + Aspirin; 06=Cilostazol 200 mg; 07=Cilostazol 200 mg + Aspirin; 08=Clopidogrel 75 mg; 09=Dipyridamole>200 mg; 10=Indobufen 200 mg; 11=Piracetam 4800 mg; 12=Sulfinpyrazone 800 mg + Aspirin>150 mg; 13=Sulfinpyrazone 800 mg; 14=Terutroban 30 mg; 15=Ticagrelor 180 mg; 16=Ticlopidine≤500 mg; 17=Ticlopidine<500 mg + Aspirin≤150 mg; 18=Triflusal 600 mg; 19=Vorapaxar 2.5 mg.

Side Direct Indirect Difference

Coef. Std. Err. Coef. Std. Err. Coef. Std. Err. P>|z|

01 02 * -.1657426 .0571844 -.3435112 .0764129 .1777686 .095427 0.062

01 03 * -.1847315 .0781792 -.1620472 .0896147 -.0226843 .1188049 0.849

01 05 * -.5071074 .0690385 -.2646671 .0757037 -.2424403 .1026388 0.018

01 06 -.4366851 .2140202 -.4589594 .1789701 .0222743 .2789891 0.936

01 09 * -.1548229 .0969977 -.0600204 .1885273 -.0948025 .2100711 0.652

01 12 * -.695748 .52272 -.6477365 .8705842 -.0480115 .9386039 0.959

01 13 * -.339073 .466198 -.2910615 .8378671 -.0480115 .9386039 0.959

01 16 -.2959207 .1513354 -.2566954 .0925568 -.0392254 .1773955 0.825

01 19 . . . . . . .

02 04 -.1546123 .1221105 -.2443079 .0757404 .0896956 .1436927 0.532

02 05 -.074821 .1532723 -.1860964 .0635733 .1112755 .1660366 0.503

02 06 -.4370721 .536194 -.2032753 .145734 -.2337968 .5556457 0.674

02 08 -.0854174 .0721192 -.1994604 .0702167 .114043 .1006556 0.257

02 11 * .3847286 .2411327 .8449986 199.4988 -.4602701 199.4987 0.998

02 12 * -.4418328 .5413254 -.4898442 .8357787 .0480115 .9386039 0.959

02 13 * -.0851578 .4869678 -.1331693 .8016428 .0480115 .9386044 0.959

02 16 -.0168884 .0806359 -.104145 .1471876 .0872567 .1678884 0.603

02 18 * .041747 .1204089 .5020179 137.0766 -.4602708 137.0765 0.997

03 04 -.291266 .0648798 -.2381573 .0988311 -.0531088 .117918 0.652

03 05 * -.2150626 .0766008 -.2435833 .0921268 .0285207 .1217744 0.815

03 06 -.2608069 .1792949 -.296038 .2068568 .0352311 .2737451 0.898

03 07 -.9110542 .5647205 .1256818 .4235268 -1.036736 .7058905 0.142

03 09 * .0186225 .1014814 .1140303 .1923166 -.0954078 .2177679 0.661

03 14 * .028947 .0525507 .375647 200.787 -.3467 200.787 0.999

03 15 * -.1228265 .0725852 .2071964 198.3316 -.3300229 198.3316 0.999

04 07 .3961973 .4202555 -.6405232 .5671591 1.036721 .7058904 0.142

04 08 .0679062 .0829926 .0931395 .0905661 -.0252333 .1228413 0.837

05 08 .0033963 .0443537 .0981288 .0826348 -.0947325 .0937856 0.312

05 09 * .3174046 .1019669 .1212657 .1751505 .1961389 .2081887 0.346

08 16 -.1376761 .3602669 .1189821 .0865185 -.2566581 .37051 0.488

10 16 * -.7143807 .2572437 .1794241 200.4165 -.8938048 200.417 0.996

12 13 . . . . . . .

16 17 * .047628 .4886427 .582574 199.7868 -.534946 199.7862 0.998

* Warning: all the evidence about these contrasts comes from the trials which directly compare them.

## *Hemorrhagic stroke*

Design-by-treatment interaction test

P-value = 0.462

Side-splitting model

Legend: 01=Placebo / No treatment; 02=Aspirin>150 mg; 03=Aspirin≤150 mg; 04=Clopidogrel 75 mg + Aspirin; 05=Dipyridamole + Aspirin; 06=Cilostazol 200 mg; 07=Cilostazol 200 mg + Aspirin; 08=Clopidogrel 75 mg; 09=Terutroban 30 mg; 10=Ticagrelor 180 mg; 11=Ticlopidine≤500 mg; 12=Ticlopidine<500 mg + Aspirin≤150 mg; 13=Triflusal 600 mg; 14=Vorapaxar 2.5 mg; 15=Ticagrelor 180 mg + Aspirin≤150 mg.

Side Direct Indirect Difference

Coef. Std. Err. Coef. Std. Err. Coef. Std. Err. P>|z|

01 02 .2359134 .137087 1.756207 .6921748 -1.520294 .7056194 0.031

01 03 1.226245 .6605326 -.2342707 .4010278 1.460516 .7727396 0.059

01 11 1.618944 1.55042 .0025051 .506795 1.616439 1.631148 0.322

01 14 . . . . . . .

02 04 -.3532492 .3053511 .6256315 .5320723 -.9788806 .6134657 0.111

02 05 -.7112468 .8839004 .1723748 .4359921 -.8836216 .9855804 0.370

02 11 .0072013 .5542601 -.6004948 .9672049 .6076962 1.11476 0.586

03 04 .1801473 .319261 -.3237781 .4910107 .5039253 .5891841 0.392

03 05 .3913027 .4923247 -.0493245 .4569974 .4406272 .6717368 0.512

03 06 * -.8714481 .3997253 -.490414 374.8189 -.3810341 374.8188 0.999

03 07 -.6752721 1.271835 1.123462 1.658675 -1.798735 2.090117 0.389

03 09 * .1439337 .2042541 -.370989 809.6212 .5149227 809.6212 0.999

03 10 * -.585208 .5663473 -.4686075 1897.926 -.1166004 1897.927 1.000

03 15 * 1.605439 .780835 -.9802198 1926.397 2.585659 1926.398 0.999

04 07 1.072353 1.63895 -.7264097 1.2971 1.798762 2.090127 0.389

04 08 -.4753129 .3256944 -.0688544 .5327603 -.4064585 .6244281 0.515

05 08 -.4929708 .2928565 -.4708169 .6479367 -.022154 .7110464 0.975

08 11 -1.110818 1.157704 .7689517 .6119259 -1.87977 1.309478 0.151

08 13 * -.9188647 .8452726 .13021 672.9367 -1.049075 672.9368 0.999

11 12 * 1.668935 1.556992 -.9593425 606.1473 2.628277 606.1519 0.997

* Warning: all the evidence about these contrasts comes from the trials which directly compare them.

## *Intracranial haemorrhage*

Design-by-treatment interaction test

P-value = 1.000

Side-splitting model

Legend: 01=Placebo / No treatment; 02=Aspirin>150 mg; 03=Aspirin≤150 mg; 04=Clopidogrel 75 mg + Aspirin; 05=Dipyridamole + Aspirin; 06=Cilostazol 200 mg; 07=Cilostazol 200 mg + Aspirin; 08=Clopidogrel 75 mg; 09=Satigrel; 10=Terutroban 30 mg; 11=Ticagrelor 180 mg; 12=Ticlopidine≤500 mg; 13=Ticlopidine<500 mg + Aspirin≤150 mg; 14=Triflusal 600 mg; 15=Vorapaxar 2.5 mg; 16=Ticagrelor 180 mg + Aspirin≤150 mg.

Side Direct Indirect Difference

Coef. Std. Err. Coef. Std. Err. Coef. Std. Err. P>|z|

01 02 .0301034 1.005014 1.366709 .5760656 -1.336606 1.15841 0.249

01 03 1.226245 .6605326 .422659 .5447622 .8035865 .8561946 0.348

01 05 -.8315796 1.204062 .8262665 .4938988 -1.657846 1.322606 0.210

01 06 -.5634037 .6297976 -.319795 .6313349 -.2436086 .891756 0.785

01 12 .0057197 1.001905 -1.250424 1.215781 1.256143 1.575417 0.425

01 15 . . . . . . .

02 04 -.3560243 .3192755 .2895476 .5363642 -.6455719 .6241982 0.301

02 05 -.7627463 .6890497 -.3902818 .3487029 -.3724645 .7765053 0.631

02 09 * -1.736099 1.638945 -2.260114 679.344 .5240153 679.344 0.999

02 14 -.4586869 .4854699 -1.792658 .7644333 1.333971 .9055602 0.141

03 04 .3695415 .3195431 -.1929602 .3344188 .5625016 .4625405 0.224

03 05 -.3113686 .2680261 .0684087 .3669206 -.3797774 .4543883 0.403

03 06 -1.148886 .3657574 -1.3925 .8132969 .2436136 .8917568 0.785

03 07 * -1.671186 1.55715 -2.08278 473.2339 .4115939 473.2331 0.999

03 10 * .1891966 .1237989 -1.507602 522.6318 1.696799 522.6318 0.997

03 11 * -.403187 .3730853 -1.537235 1315.46 1.134048 1315.46 0.999

03 16 * 1.201061 .4658655 -1.835304 1301.272 3.036366 1301.272 0.998

04 08 -.5483486 .2174598 -.6985112 .4171524 .1501626 .470431 0.750

05 08 -.3570979 .1292697 .1999969 .4033668 -.5570947 .4235746 0.188

08 12 -1.400256 1.119594 -.1441231 1.108359 -1.256133 1.57542 0.425

08 14 -.8524652 .6937999 .481503 .5819632 -1.333968 .9055602 0.141

12 13 * 1.668935 1.553989 .4178629 606.0841 1.251072 606.0887 0.998

* Warning: all the evidence about these contrasts comes from the trials which directly compare them.

## *Major bleeding*

Design-by-treatment interaction test

P-value = 0.085

Side-splitting model

Legend: 01=Placebo / No treatment; 02=Aspirin>150 mg; 03=Aspirin≤150 mg; 04=Clopidogrel 75 mg + Aspirin; 05=Dipyridamole + Aspirin; 06=Cilostazol 200 mg; 07=Cilostazol 200 mg + Aspirin; 08=Clopidogrel 75 mg; 09=Dipyridamole>200 mg; 10=Piracetam 4800 mg; 11=Sulfinpyrazone 800 mg + Aspirin>150 mg; 12=Sulfinpyrazone 800 mg; 13=Terutroban 30 mg; 14=Ticagrelor 180 mg; 15=Ticlopidine≤500 mg; 16=Ticlopidine<500 mg + Aspirin≤150 mg; 17=Triflusal 600 mg; 18=Vorapaxar 2.5 mg; 19=Ticagrelor 180 mg + Aspirin≤150 mg.

Side Direct Indirect Difference

Coef. Std. Err. Coef. Std. Err. Coef. Std. Err. P>|z|

01 02 * .4526523 .2252794 .9300946 .3406432 -.4774423 .3931099 0.225

01 03 * .8236462 .3438548 .5928899 .3016755 .2307563 .4488105 0.607

01 05 * 1.268312 .3749669 .1757498 .1793255 1.092562 .4069571 0.007

01 06 -.5615151 .6733328 -.2292709 .3917891 -.3322443 .7790223 0.670

01 09 * -.1577981 .5850584 -1.822072 .821365 1.664273 .9845401 0.091

01 11 * .9339279 .7904863 5.547475 2.994825 -4.613547 3.175919 0.146

01 12 * .484246 .8322768 5.097794 3.006125 -4.613548 3.175918 0.146

01 15 .2953303 .7989693 -.0882485 .4223261 .3835788 .9037208 0.671

01 18 . . . . . . .

02 04 .5938116 .2751551 .5512038 .3144555 .0426078 .4151292 0.918

02 05 -.7275257 .3620691 .2055665 .236223 -.9330923 .431326 0.031

02 06 -.9473348 .8734139 -.9197643 .3739992 -.0275704 .9501195 0.977

02 10 * -1.920262 1.087783 -2.130281 504.4939 .2100197 504.4934 1.000

02 11 * 2.593021 1.491481 -2.020522 2.042062 4.613544 3.175917 0.146

02 12 * 2.143339 1.514045 -2.470209 2.058601 4.613549 3.175917 0.146

02 15 -.6463481 .4595225 -.5745095 .5281238 -.0718387 .700054 0.918

02 17 -.8841055 .3468456 -1.123936 .7756859 .2398308 .847625 0.777

03 04 .4027456 .2441009 .6617141 .3013911 -.2589685 .3815199 0.497

03 05 * -.0586502 .201261 -.4746297 .3244502 .4159795 .3811838 0.275

03 06 -.9353365 .3444179 -1.194151 .574851 .2588148 .6701323 0.699

03 07 .2295986 .7533989 -.6706824 .8721378 .9002811 1.152544 0.435

03 09 * -1.215569 .5158486 -1.83492 .7609831 .6193515 .782225 0.428

03 13 * .003455 .2464195 -1.384424 424.8963 1.387879 424.8964 0.997

03 14 * -.2014664 .3314873 -1.384228 887.3576 1.182762 887.3576 0.999

03 19 * 1.384655 .4795216 -1.824043 1166.618 3.208698 1166.618 0.998

04 07 -1.147616 .8521677 -.2473653 .7759948 -.9002511 1.152543 0.435

04 08 -.9000984 .2936814 -.7408712 .3335379 -.1592272 .4444055 0.720

05 08 -.1404291 .2704545 -.1723535 .3616965 .0319245 .4516305 0.944

05 09 * -1.519364 .4624312 .0397016 .6755591 -1.559065 .6410789 0.015

08 15 -.4840635 .620746 -.2956264 .4703104 -.1884372 .7787923 0.809

08 17 -.8524657 .7329298 -.6126359 .4257772 -.2398298 .8476274 0.777

11 12 . . . . . . .

15 16 * .04652 .6323381 .0180461 307.0145 .0284739 307.0151 1.000

* Warning: all the evidence about these contrasts comes from the trials which directly compare them.

# **Supplementary 10. Comparison-adjusted funnel plot for a network of interventions by outcome**

In the horizontal axis it is reported the difference of each study estimate from the pooled effect from the simple pairwise meta-analysis for the respective comparison. Studies that lie symmetrically around the zero suggest absence of small-study effects (lie symmetrically around the zero line).^1^

## *All strokes*

## *All-cause mortality*

## *Ischemic stroke*

## *Cardiovascular event*

## *Hemorrhagic stroke*

## *Intracranial haemorrhage*

## *Major bleeding*

# *Reference*

^1^Chaimani A, Salanti G. Using network meta-analysis to evaluate the existence of small-study effects in a network of interventions. *Research Synthesis Methods* 2012;3: 161-176.
